# Design of an abiotic unimolecular three-helix bundle†

**DOI:** 10.1039/d4sc07336c

**Published:** 2024-12-04

**Authors:** Shuhe Wang, Johannes Sigl, Lars Allmendinger, Victor Maurizot, Ivan Huc

**Affiliations:** a Department Pharmazie, Ludwig-Maximilians-Universität München Butenandtstraße 5–13 Munich D-81377 Germany ivan.huc@cup.lmu.de; b Université de Bordeaux, CNRS, Bordeaux Institut National Polytechnique, CBMN (UMR 5248), Institut Européen de Chimie Biologie 2 Rue Escarpit Pessac 33600 France

## Abstract

Starting from the solid state structure of *C*_3_-symmetrical homochiral parallel trimolecular bundle of three aromatic helices held together by intermolecular hydrogen bonds, we have used simple rational principles and molecular modelling to design a similar heterochiral structure where one helix had an opposite orientation and handedness. A rigid and a flexible linker to connect these helices and transform the bundle into a unimolecular object were designed and synthesized. Model sequences with two helices and one linker were then prepared. Their conformations were investigated in solution by nuclear magnetic resonance and circular dichroism, in the solid state by X-ray crystallography, and by molecular dynamics simulations, overall supporting the initial design. A final 6.9 kDa unimolecular three-helix bundle was then prepared using a fragment condensation approach. Solution studies support the formation of the targetted tertiary fold in the case of the rigid linker, thereby validating the overall approach.

## Abstract 2

A unimolecular three-helix bundle aromatic foldamer in which each helix interacts with the two others was designed by stepwise transformation of a parallel trimolecular three-helix bundle.

## Graphical abstract

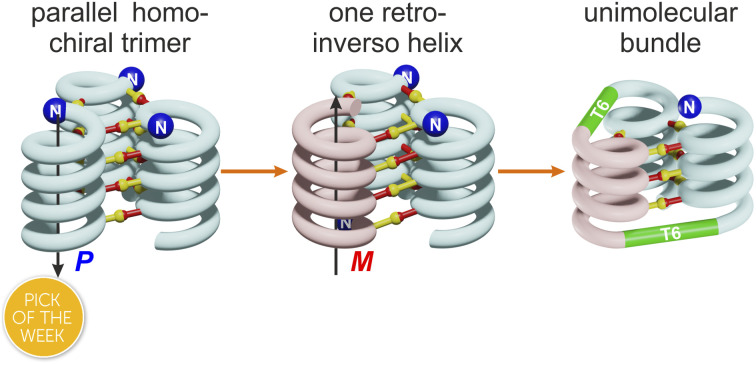

## Introduction

Abiotic foldamers are defined as artificial folded architectures chemically remote from proteins and nucleic acids, the biopolymers from which they are inspired. Foldamers with aryl rings in their main chain constitute the most developed class of abiotic foldamers.^1–7^ Interest for such compounds stems from the expectation that using distinct chemical backbones might give access to distinct shapes and functions, possibly beyond the reach of biopolymers. For example, many aromatic foldamers fold in organic solvents. The relative rigidity associated with the introduction of aryl rings in the main chain facilitates the prediction of the preferred conformations of such foldamers. Over the years, various types of aromatic monomers have been produced and sequences have been synthesized that can fold into helices^8–11^ or sheets.^12,13^ Helices possessing a sizable cavity can be used for endomolecular recognition,^14–21^ while the decoration of aromatic helices with proteinogenic and water solubilizing side chains can be used to recognize large surface areas of proteins^22^ and interfere with *e.g.* amyloid proteins or DNA binding proteins.^23–26^ Such helices have also been shown to aggregate in solution to form multistranded helices^9,27–29^ or stacked structures.^30,31^

**Fig. 1:**
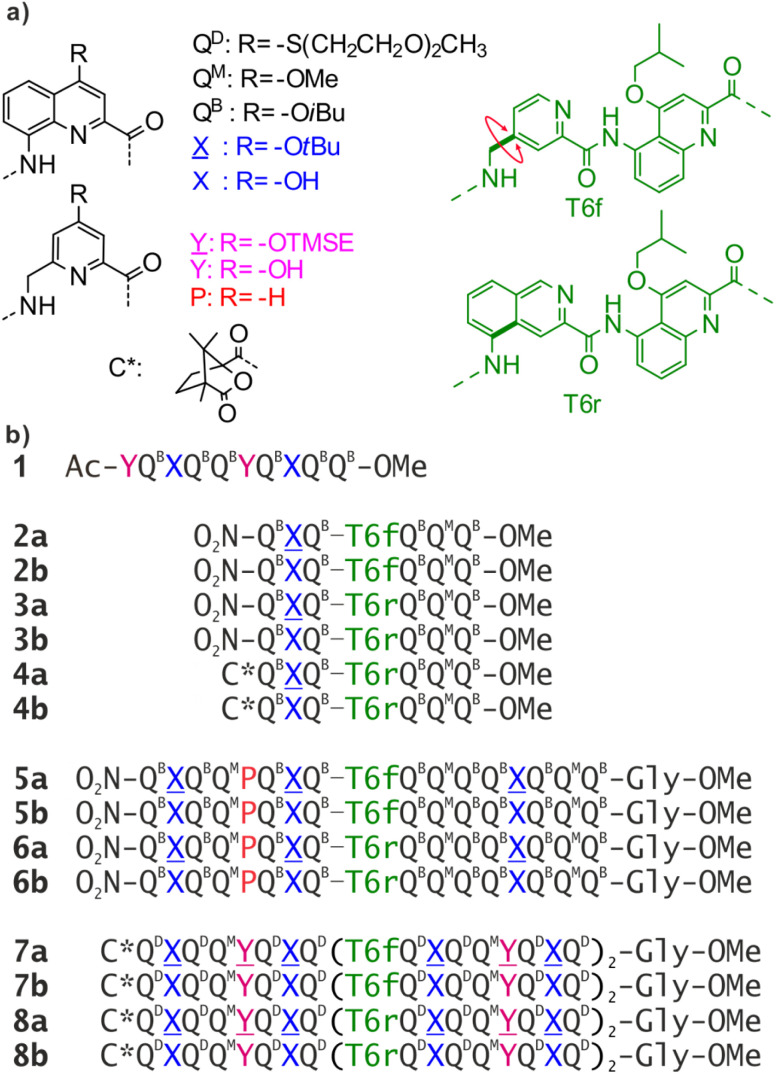
(a) Structure of Q^D^, Q^B^, Q^M^, X, Y, P, T6f, and T6r monomers as well as N-terminal chiral C* group. X̲ and Y̲ are the hydroxy protected precursors of X and Y, respectively. TMSE = 2-trimethylsilylethyl. (b) Oligoamide foldamer sequences. The Gly at the C terminus stands for glycine. In sequences ending with an 8-nitro group, this group replaces the terminal amine.

In peptides, sophisticated functions are rarely associated with an isolated α-helix or β-strand. Instead, most protein functions emerge at the level of tertiary structures that are complex objects consisting of several helices or sheets. While the design of artificial proteins has been thriving,^32–35^ tertiary structures based on abiotic backbones are still in their infancy. Our own efforts have consisted in assembling secondary motifs based on oligoamides of 8-amino-2-quinoline carboxylic acid bearing different solubilising side chains in position 4 (Q^D^, Q^B^, Q^M^ in Fig. 1a). Oligomers of these δ-amino acids fold into extremely stable 2.5 aromatic helices in all types of solvents (Fig. S1†).^36,37^ Such helices are easily produced by automated solid phase synthesis,^38^ and constitute convenient building blocks to be assembled into larger structures.^39^

In α-peptides, the bundling of several α-helices by multimolecular self-assembly or by folding of a single sequence containing multiple α-helical segments separated by loops is one of the best understood motifs. Such structures can be designed reliably,^40–42^ and have inspired the development of β-peptidic^43–45^ and urea-based^46–48^ foldamer helix bundles as well as bundles of 3_10_ helices.^49^ We have followed the same path and used helix–helix interactions to produce the first abiotic tertiary structures.^50^ Monomer X, an analogue of Q, was developed for this purpose (Fig. 1a). The 4-hydroxy group of X protrudes from the aromatic helix and may hydrogen bond to the amide carbonyl group of another helix in chlorinated solvents. Similarly, P and Y constitute analogues of Q and X, respectively, in which the quinoline benzene ring has been trimmed to avoid possible steric clashes within helix bundles.

**Fig. 2:**
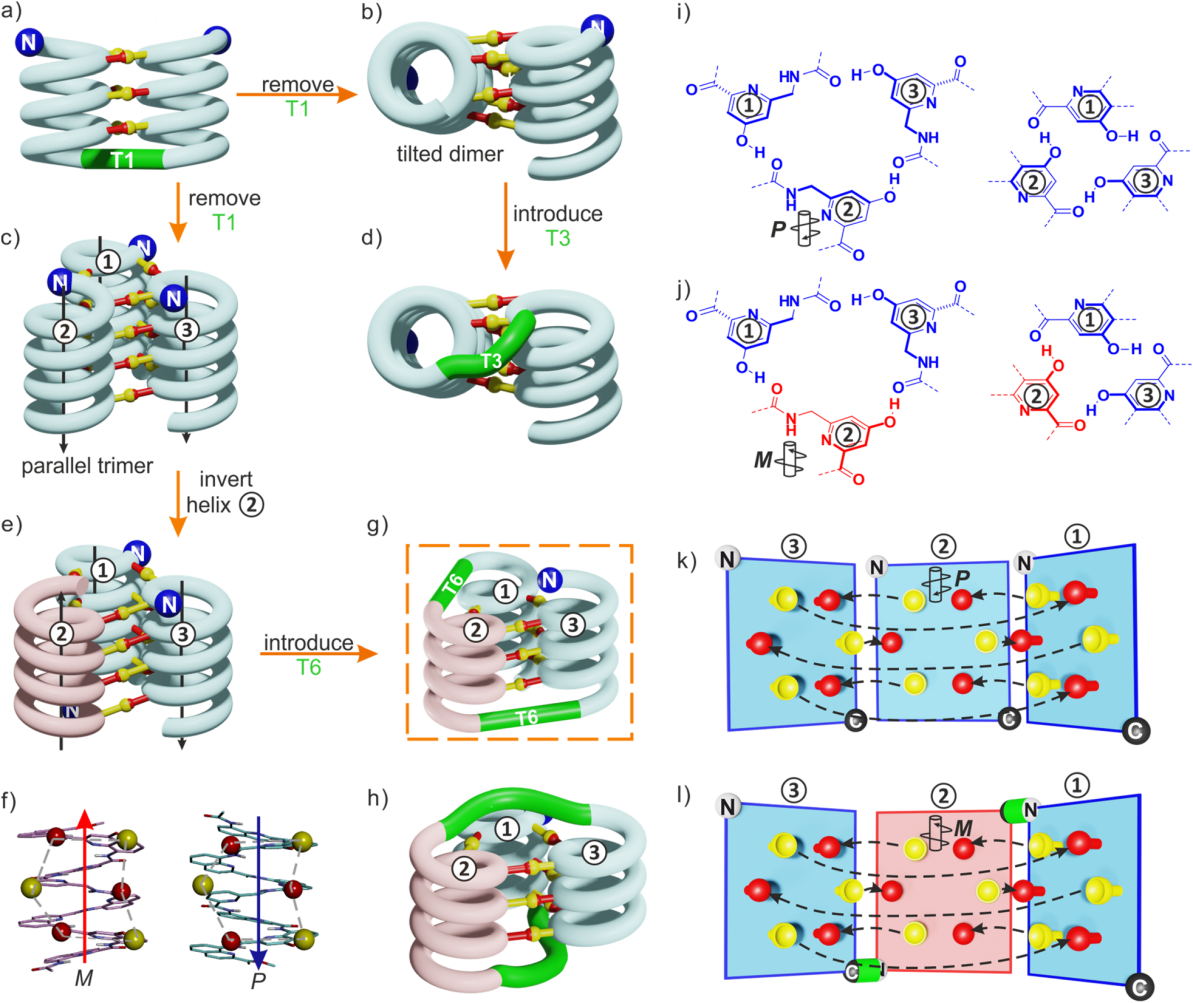
(a) Schematic representation of a homochiral helix-T1-helix structure. After removing the T1 turn unit, single helices may assemble in tilted dimers (b) or in a parallel trimer (c). As shown in (c), the three helices are parallel in the trimer (black arrows) and the N termini (blue balls) are on the same side of the structure. (d) An oligoethylene glycol-based T3 turn unit was introduced to stabilize an intramolecular tilted arrangement.^65^ (e) Model of a trimer after inverting the orientation and the handedness of one helix. *P* helices and *M* helices are colored in blue and red, respectively. The C terminus of helix ② is then close to the N termini of both helices ① and ③. (f) Illustration of the similar arrangement of hydrogen bond donors (yellow balls) and acceptors (red balls) after inverting the handedness and orientation of a helix. (g) Connection of the C terminus of helix ② to the N terminus of helix ③ and of the N terminus of helix ② to the C terminus of helix ①. (h) Connection of the C terminus of helix ② to the N terminus of helix ① and of the N terminus of helix ② to the C terminus of helix ③. (i) Hydrogen bonding motif between X units and between Y units in the parallel trimer shown in (c). (j) Hydrogen bonding motif between X units and between Y units in the trimer shown in (e). The color code is the same as in (e). (k) “Open-book” view of the hydrogen bond patterns of the homochiral trimer. (l) “Open-book” view of the hydrogen bond patterns of the representation shown in (g). In (k) and (l), the helix face is represented as a plane, with the N terminus shown as a white ball, the C terminus shown as a black ball and the linker T6 shown as a green stick. Planes with a blue and red frame correspond to *P* and *M* helices, respectively. Dashed-arrows indicate hydrogen bonds.

By carefully arranging the hydroxy groups of X and Y units at the helix surface and choosing a proper turn unit to covalently link two helices, a first helix-turn-helix motif was designed in which two helices are held with their axes parallel to each other by inter-helix hydrogen bonds (Fig. 2a and S2†).^50^ In this first generation, the helices of the bundle were by design set to both have the same right-handed (*P*) or left-handed (*M*) handedness while in a second generation of helix-turn-helix motif, a *P* helix was linked to an *M* helix (Fig. S2†).^51^ These tertiary structures are robust enough to undergo further assembly into quaternary motifs upon introducing additional hydroxy groups to form intermolecular hydrogen bonds.^52^ Nevertheless, we discovered that the parallel arrangement of two helices is not the most stable. When the connecting turn element (T1 or T2 in Fig. S2†) is absent as, for example, in sequence 1 (Fig. 1b), single helices associate in parallel trimers (Fig. 2c) or in titled dimers (Fig. 2b), not in parallel dimers.^50^ These different aggregates coexist in solution making the selective formation of one or the other challenging. The reason for their prevalence is that they both allow the release of some unfavorable helix torsion present in the helix-turn-helix structures without hampering hydrogen bonding.^53,54^

The parallel trimer was a serendipitous discovery and the first motif in which three abiotic helices were assembled in such a way that each helix interacts with the two others (Fig. 2i). Here we present how we successfully transformed this trimolecular object into a unimolecular helix-turn-helix-turn-helix tertiary structure analogous to a pattern common in proteins, for example in the B domain of protein A (Fig. S3†).^55,56^ We first explored the behavior of shorter models 2–6 before implementing the same design principle in 7 and 8 which contain three helical segments. This achievement not only generated the most complex abiotic tertiary structure known to date. It also allowed us to streamline the design principles, computational approach, as well as synthetic and purification strategies. Routinely accessing such 6.9 kDa abiotic folds may become a realistic prospect in the near future.

## Results and discussion

### Redesign of the relative helix orientation

**Fig. 3:**
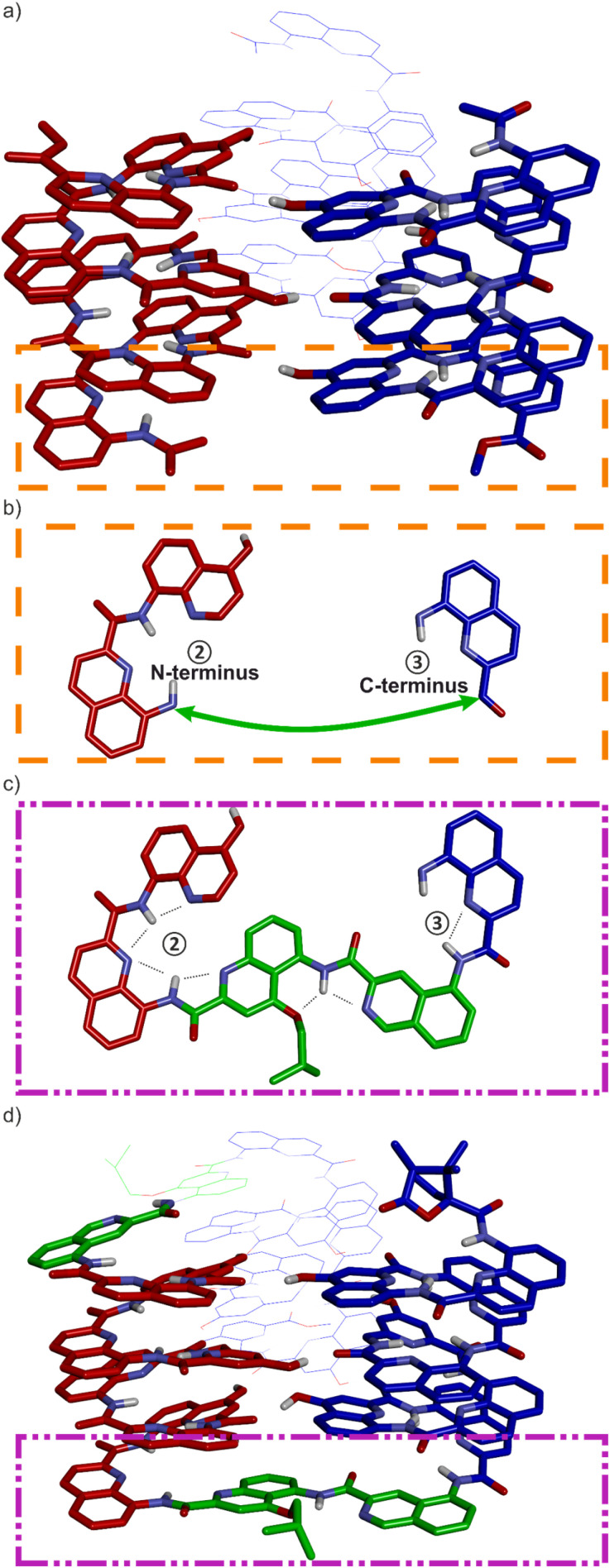
(a) Energy-minimized model of a heterochiral (blue = *P*, red = *M*) trimer of Ac-QXQQYQXQ-OMe. Helices in the front are in stick representation. The helix in the back is in line representation. (b) Top view of the terminal units within the orange dashed line box of (a). A green arrow shows where the turn unit should be placed. (c) Top view of the terminal units within the purple dashed line box of (d). (d) Energy-minimized model of 8b. Color coding is as in (a). The T6r turn is shown in green. In all structures, side chains on Q units have been omitted for clarity.

The design of a unimolecular three-helix bundle derived from the crystal structure of the parallel trimer of 1 required to meet two distinct challenges. The design and synthesis of proper linkers, *i.e.*, turn units, is addressed in the next section. This section deals with the first challenge which is to organize the helices in space so that they may conveniently be connected. The crystal structure of 1 is a *C*_3_-symmetrical trimeric assembly (Fig. 2c and S4†). In this structure, the three identical helices named ①, ②, and ③ in Fig. 2 have the same handedness (all right-handed, *P*, or all left-handed, *M*) and are arranged in a head-to-head manner. Thus, the N terminus of helix ② is close to the N termini of helices ① and ③ but distant from their C termini. Covalently connecting two N termini and two C termini using diacid and diamine linkers, respectively, would be a way to transform (1)_3_ into a unimolecular three-helix bundle. However, this approach was not considered because it would require a complicated synthetic approach. Another approach would be to covalently connect the N terminus of a helix to the C terminus of an adjacent helix, but this would require a long and difficult-to-design linker. Inverting the orientation of one helix would solve that problem by reducing the distance between N and C termini. However, the hydrogen bonding motif would then no longer promote helix–helix associations. Instead, donors would face other donors, and acceptors would face acceptors. The solution comes from the inversion of both the orientation and the handedness of one helix as this also preserves the position of the hydrogen bond donors and acceptors (Fig. 2e, f, i–l). This operation is well-known in the so-called α-helical retro-inverso peptides,^57–59^ and has been used by us to design the *PM*/*MP* helix-turn-helix tertiary fold.^51^ Associations between *P* and *M* peptidic helices have also been reported.^60–63^ Thus, a molecular model of a trimeric bundle where helix ② has an orientation and a handedness both opposite to those of the two other helices was built and energy-minimized in Maestro (Fig. 3a and S4†).^64^ The intermolecular hydrogen bond patterns extracted from the heterochiral trimer molecular model were very similar to those of the homochiral *C*_3_-symmetrical trimer. Nevertheless, one should recall that 1 trimerizes into a homochiral *PPP*/*MMM* (1)_3_ helix bundle. The *PPM*/*MMP* heterochiral trimer was not observed in solution or in the solid state and must therefore be inherently less stable.

### Linker design and synthesis

Having reduced the distance between the C and N termini to be connected, the next step was the design of the linker. In an earlier study, we succeeded to design linkers for a tilted helix dimer motif (Fig. 2d),^65^ and could experience how delicate this exercise can be. For the earlier parallel helix-turn-helix designs, the turn was selected first and the helix–helix interactions were designed next.^50,51^ In the present case, this approach was not considered as we preferred to take advantage of an existing pattern of hydrogen bonds. First, sequence length was adjusted so as to minimize the distance between the termini to be connected – plus or minus one unit may result in significant distance variations. This generated a design with a total of nine hydrogen bonds, compared to twelve intermolecular hydrogen bonds in the structure of (1)_3_. As depicted in Fig. 2g and h, two distinct helix–helix connection strategies may be envisaged based on an amino acid turn unit: (N terminus)-①-turn-②-turn-③-(C terminus) or (N terminus)-③-turn-②-turn-①-(C terminus) (Fig. 2g). In other words, the N terminus of the central helix ② is close to the C termini of both ① and ③ but its spatial relationships to the two differ. In both strategies, the same linker would be used twice but the linkers for one strategy or the other differ. We discarded the arrangement shown in Fig. 2h because the suitable linkers between two helices that we found were also overlapping with the terminal cross section of the other helix, de facto preventing elongation of the C and N termini of the final object. The other arrangement shown in Fig. 2g does not have this impediment. Note that, at this stage, no consideration of absolute configuration is needed and the system can be treated as racemic. If ① is *P*-helical, ② is *M* and ③ is *P*. If ① is *M*-helical, ② is *P* and ③ is *M*.

**Fig. 4:**
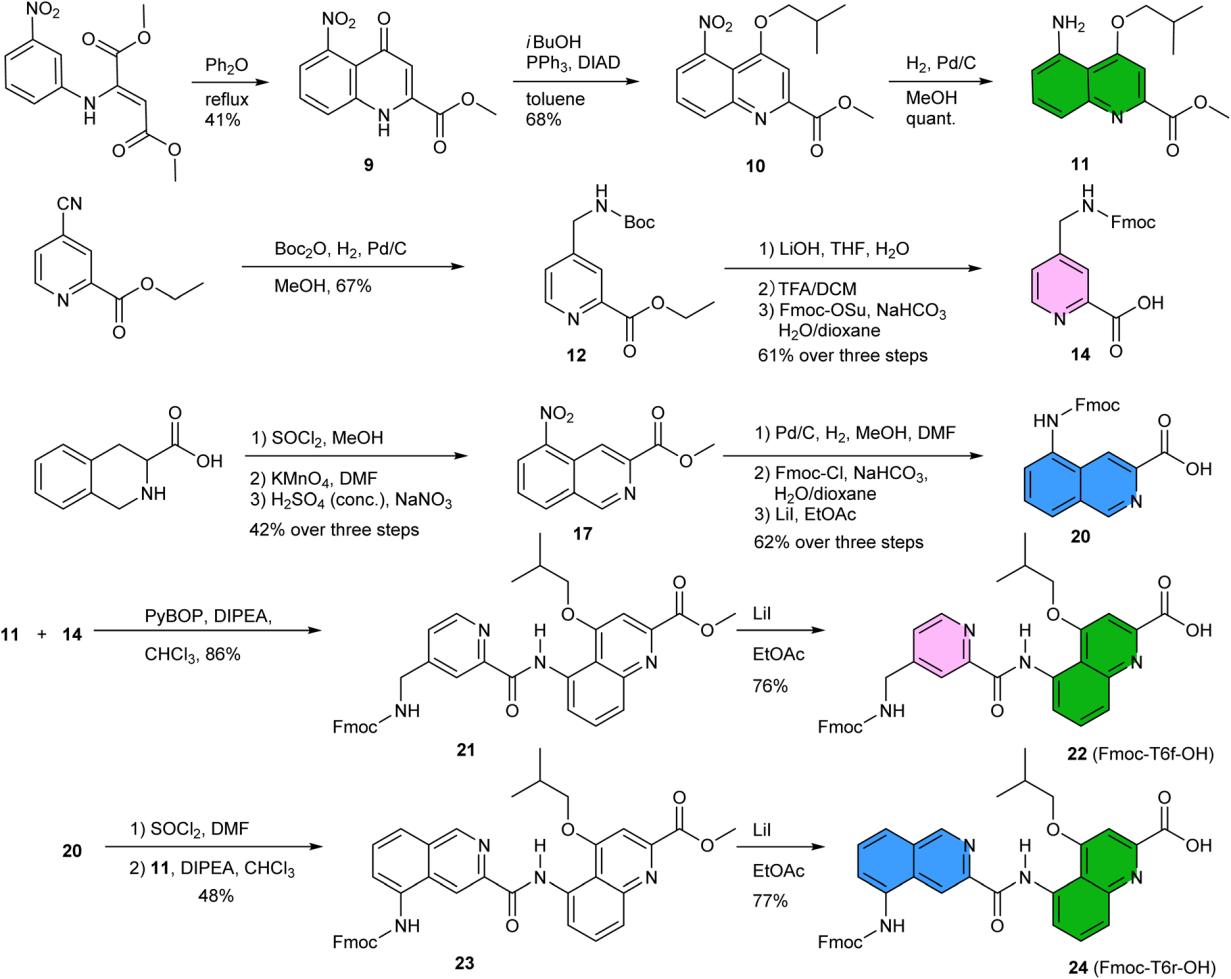
Synthesis of Fmoc-T6f-OH and Fmoc-T6r-OH. Compound 13 (not shown) is the saponification product of 12. Compounds 18 and 19 (not shown) are the products of the hydrogenation of 17 and of the subsequent Fmoc installation, respectively.

The linker should preferably be relatively rigid to reduce the possibility of arrangements other than those intended. The linker should also not create strain that would risk destabilizing the structure. Keeping this in mind, models with different connections between the helix termini (green arrow in Fig. 3b) were built and energy-minimized. When strain was not apparent in the minimized structure under the form of *e.g.* helix distortion, a further test was to cut a bond in the linker and energy-minimize again. Any important conformational change at this stage would be interpreted as strain and the corresponding linker would be discarded or subjected to improvement. Finally, the synthetic accessibility was also considered to select the linker. A number of trials led to the design of two analogous turn units, T6f and T6r (Fig. 1a, 3c and d). T6f is a diamide of 4-aminomethyl pyridine 2-carboxylic acid and 5-amino quinoline 2-carboxylic acid, while T6r is a diamide of 5-amino isoquinoline 3-carboxylic acid and 5-amino quinoline 2-carboxylic acid. T6f is more flexible due to the presence of an additional rotatable bond at the CH_2_ group. The linker units were produced with a free acid function and an Fmoc-protected amine, that is, ready for solid-phase synthesis (Fig. 4, see ESI† for details).

The synthesis of 5-nitroquinoline 10 is similar to that of the 8-nitro precursor of Q^B^,^66^ starting from 3-nitroaniline instead of 2-nitroaniline. Of note, the cyclization that produces 9 yields an equal amount of the 7-nitro isomer which can be used in other foldamer architectures.^67^ The two isomers can be separated by selective crystallization. Similarly, 14 represents a regioisomer of Fmoc-P-OH, albeit obtained by a different route. Hydrogenation of the commercially available ethyl 4-cyano-2-pyridinecarboxylate in MeOH and in presence of Boc_2_O produced 12 without observable transesterification. The synthesis of precursor 20 starts from commercially available tetrahydroisoquinoline 3-carboxylic acid. The nitration step is selective and the 5-nitro regioisomer was isolated in 80% yield by precipitation. The acylation of 11 by 14 using PyBOP activation or by 20 produced Fmoc-T6f-OH and Fmoc-T6r-OH, respectively. Overall, these syntheses worked quite well and needed not be repeated for this study. About half of the steps required chromatographic purification. We surmise that a full optimization would allow to improve some yields further, to increase the scales, and to avoid some chromatographic steps.

### Validation of the linker design in helix-turn-helix models

Sequences 7b and 8b (Fig. 1b) were designed to fold into the desired ∼6.9 kDa three-helix tertiary structure. They both have three identical QXQQYQXQ subdomains linked by T6f in 7b or T6r in 8b and a (1*S*)-camphanyl group at the N terminus that quantitatively biases the handedness of the N-terminal helix to *P*.^68^ As for 4b, chirality can be useful to investigate conformations by circular dichroism (CD). Before undertaking the synthesis and investigation of such large compounds, we sought a validation of the turn units T6r and T6f in smaller helix-turn-helix motifs.

We thus prepared three sequences equivalent in length to Q_3_-turn-Q_3_, 2b, 3b and 4b. Sequences 2b and 3b are achiral, with T6f and T6r as turn units, respectively. Sequence 4b is a chiral analogue of 3b, bearing a (1*S*)-camphanyl group so that the N-terminal helix has *P* handedness.^68^ All three sequences have a single X monomer in position two of the first helical domain. The hydroxy group of this X unit can potentially form an intramolecular hydrogen bond with a carbonyl group of the second Q_3_ helical domain. In these short oligomers, the second residue after the turn is a Q^M^ monomer in replacement of X in 7b and 8b, where this residue should interact with the third helix.

**Fig. 5:**
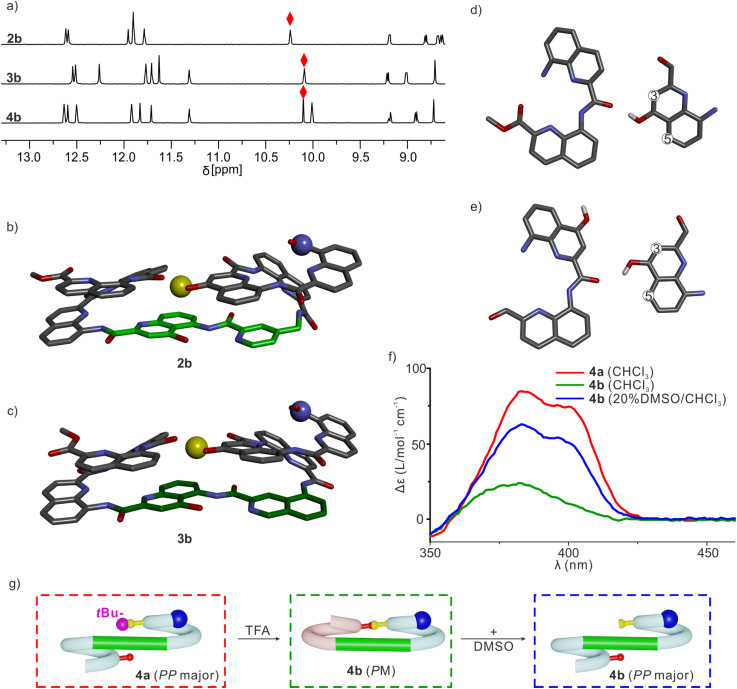
(a) ^1^H NMR spectra (500 MHz, CDCl_3_) of 2b, 3b and 4b. Hydrogen-bonded O*H* signals are marked with red diamonds. Solid state structures of 2b (b) and 3b (c). Both structures are shown in stick representation. Hydrogen atoms (except the OH proton), side chains and included solvent molecules are omitted for clarity. T6f and T6r units are colored in light green and dark green, respectively. The hydrogen bond donor is shown as a yellow ball. The N terminus is shown as a blue ball. Only the *PM* enantiomers are shown. (d) Intramolecular hydrogen bonding extracted from (c), which resemble that observed in previously described shifted dimers. (e) Intramolecular hydrogen bonding in molecular models of 7b and 8b. (f) CD spectra of 4a and 4b in chloroform or in 20% DMSO/chloroform. (g) Cartoon representation explaining the different CD intensities. The *t*Bu protecting group is shown as a purple ball. Its bulkiness favors the *PP* conformer of 4a, whereas 4b adopts a *PM* conformation in CDCl_3_. DMSO disrupts the *PM* conformation of 4b allowing for the prevalence of the *PP* conformer as in 4a.

Sequences 2a, 3a and 4a (Fig. 1b), the protected precursors of 2b, 3b and 4b, were synthesized on solid phase using previously reported methods.^38^ The synthesis was performed on a super acid sensitive resin (SASRIN™) so that mildly acidic resin cleavage preserved *t*Bu-ether protection of the X̲ monomer. The C-terminal carboxy group was then methylated and the sequences were purified in their protected form. After TFA-mediated cleavage of the *t*Bu group, they were purified in their deprotected form. The ^1^H NMR spectra of 2a, 3a and 4a in CDCl_3_ showed one set of sharp signals (Fig. S5†), and so did the spectra of 2b, 3b and 4b (Fig. 5a). However, the signal patterns of the C*H*_2_ protons belonging to *i*Bu side chains indicate that *P*/*M* handedness interconversion of the helical segments of 2a and 3a is fast on the NMR time scale at 298 K whereas it is slow for 2b and 3b, indicating higher conformational stability of the latter (Fig. S6†). For 2b, 3b and 4b, ^1^H,^15^N Heteronuclear Single Quantum Coherence (HSQC) spectra allowed for the indirect assignment of the OH resonance as an exchangeable proton that does not correlate with ^15^N (Fig. 5a and S7–9†). In all cases, the OH proton signal is found above 10 ppm indicating that this proton is involved in a hydrogen bond.

The solid-state structures of 2b and 3b were elucidated using single crystal X-ray diffraction analysis. Their conformations are similar and both are very close to the initial molecular models. The helix-turn-helix motif is heterochiral, that is, *PM* or *MP*, meaning that T6r and T6f promote a reversal of helix handedness as desired. The hydroxy group of X is involved in the expected intramolecular hydrogen bond (Fig. 5b and c). A slight deviation from the initial design concerns the relative orientation of the hydrogen bond donor and acceptor (Fig. 5d and e). The observed orientation, with the hydroxy group pointing to the position 3 of the quinoline ring to which it belongs (Fig. 5d), is similar to that of a previously described arrangement that we called “shifted” interface (Fig. S10†).^69^ In contrast, in the predicted arrangement (Fig. 5e) and in the structure of (1)_3_ (Fig. 2i),^50^ the hydroxy group points toward the position 5 of the quinoline ring. Molecular Dynamics (MD) simulations starting from the solid-state structure of 3b showed that the two hydrogen bond patterns alternate within a simulation time as short as 1 ns, suggesting fast dynamics in these smaller model compounds (Fig. S11†).

The CD spectra of chiral sequences 4a and 4b in solution were consistent with the solid-state structure of 3b (Fig. 5f). The relatively weak CD band of 4b is in agreement with its two helical segments having opposite handedness and largely cancelling each other's CD contribution. In contrast, the bulky *t*Bu ether of 4a may disfavor a *P*-turn-*M* arrangement with both helices on the same side (Fig. 5g). Having both Q_3_ helices on opposite sides of the turn unit would then favor a *P*-turn-*P* arrangement,^70,71^ as reflected in its intense positive CD band. Adding DMSO to a CHCl_3_ solution of 4b disrupts the intramolecular hydrogen bond, resulting in an increase of the proportion of the *P*-turn-*P* conformation and thus in an enhancement of CD intensity (Fig. 5f and g). Altogether, these results hint at T6r and T6f performing well at promoting the desired conformations. We thus proceeded with the examination of longer model systems.

Sequences 5b (with T6f) and 6b (with T6r) are equivalent in length to Q_8_-turn-Q_8_ and represent achiral analogues of the two N-terminal helix-turn-helix segments of 7b and 8b, respectively. In 5b and 6b, the OH groups intended to promote interactions between helices ③ and ② of 7b and 8b have been preserved, and the OH groups intended to promote interactions with helix ① have been removed. Thus, 5b and 6b contain three X units in total, compared to three X units and two Y units for the first two helices of 7b and 8b. The protected precursors 5a and 6a (Fig. 1b) were synthesized on solid phase using the same protocol as for 2a, 3a and 4a, but on a different resin. With a Gly-HMBA AM resin (4-(hydroxymethyl)benzoyl-aminomethyl polystyrene with a preloaded glycine), resin cleavage in presence of methanol under basic conditions directly yields the C-terminal methyl ester while preserving *t*Bu-ether protection on the X̲ monomers. Sequences 5 and 6 thus have a glycine methyl ester at their C terminus.

**Fig. 6:**
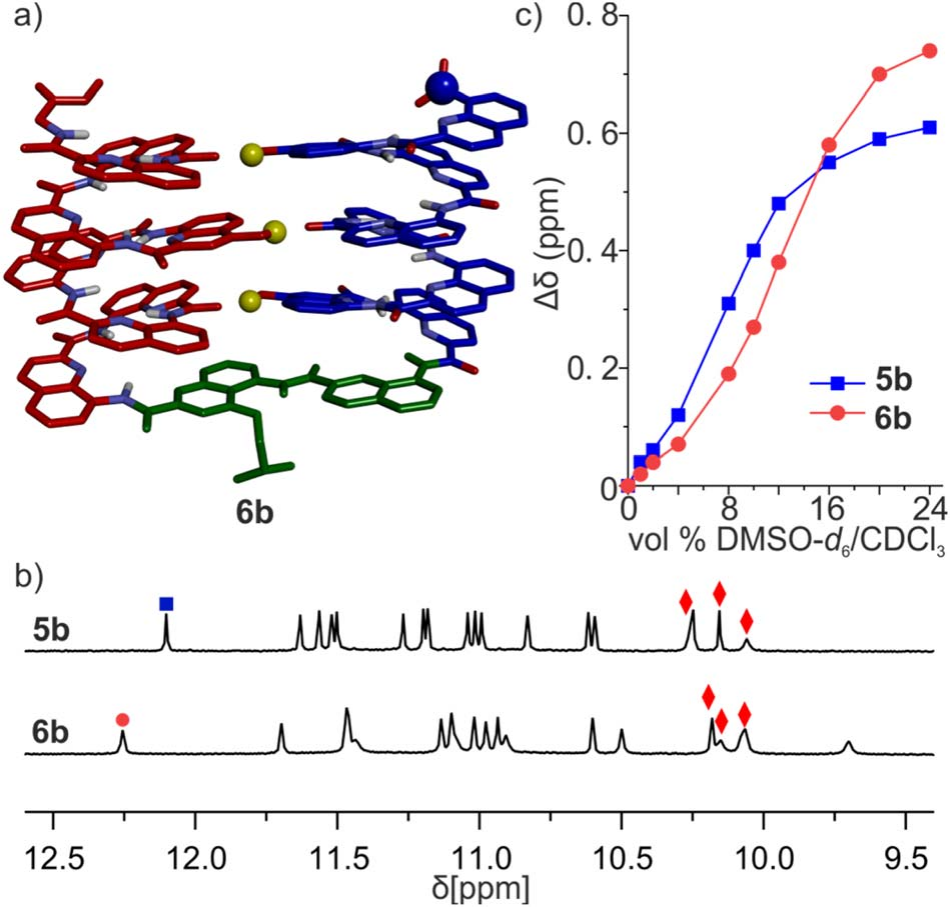
(a) Energy-minimized model of the 6b-T conformer of 6b. The molecule is shown in stick representation. Non-polar hydrogen atoms and side chains (except that of the T6r turn) have been removed for clarity. Hydroxy protons are shown as yellow balls. The intramolecular hydrogen bond pattern is similar to what we observed in the parallel trimer. (b) Extracts of ^1^H NMR spectra (500 MHz, CDCl_3_) of 5b and 6b. The signals marked with red diamonds were assigned to hydroxy protons involved in hydrogen bonding. (c) Variations of the chemical shift of ^1^H NMR signals marked with blue boxes and red dots in (b) upon the addition of DMSO-*d*_6_ to CDCl_3_ solution of 5b and 6b, respectively.

Handedness interconversion in each of the longer helices of 5a and 6a is slow on the NMR timescale, unlike in the shorter helices of, *e.g*., 2a. This allows for the observation of diastereomeric conformers as distinct sets of signals at 298 K (Fig. S5†). Thus, the ^1^H NMR spectrum of 5a showed two species in a 1 : 0.8 ratio, which correspond to *PP*/*MM* and *PM*/*MP* diastereomers. In contrast, the spectrum of 6a shows one set of signals assigned to the *PP*/*MM* conformers by extension of the preference of 4a for the *PP* conformation. The different spectra of 5a and 6a reflect different behaviors of T6f and T6r. Due to its rigidity, the latter conveys handedness from one helix to the next, whereas the former disrupts helix handedness communication.^70,71^ Nevertheless, after side chain deprotection, the ^1^H NMR spectra of 5b and 6b both show a single set of signals in CDCl_3_ (Fig. 6b), indicating that a single diastereomeric conformer prevails and thus that quantitative helix handedness communication through the turn units takes place, as observed above for 2b and 3b. O*H* resonances were assigned as for 2a and 3a (Fig. 6b, S12 and 13†) and appeared above 10 ppm, indicating their involvement in hydrogen bonds. Diffusion-Ordered Spectroscopy (DOSY) shows that 6b has a larger diffusion coefficient than 6a (Fig. S14†). Since 6a is a monomer, this suggests that 6b is monomeric as well and thus that all hydrogen bonds are intramolecular. The prevalent conformers of 5b and 6b in CDCl_3_ were also prevalent in CD_2_Cl_2_ (Fig. S15 and 16†). This was examined because the relative stability of (1)_3_ had been shown to differ in these two solvents.^50^

We then evaluated the stability of these conformers by ^1^H NMR upon adding DMSO-*d*_6_ to CDCl_3_ solutions (Fig. 6c, S17 and 18†). DMSO competes for hydrogen bonding and is known to disrupt tertiary folds mediated by hydrogen bonds, resulting in variations of chemical shift values.^50,51,53,72^ The conformational change was observed in both species, yet their transition points differed slightly: around 8% (vol/vol) DMSO for 5b, and 12% for 6b. The sequence having the more rigid linker thus appears to be more stable.

The results above are consistent with 5b and 6b folding as a U-shaped helix-turn-helix structure stabilized by the turn unit geometry and three intramolecular inter-helix hydrogen bonds. Crystals of 5b and 6b were obtained but did not diffract at atomic resolution and their solid-state structure could not be solved. Their conformations were thus further investigated by MD simulations. Building on what was observed for 3b, two different models of 6b were built. In one model, the OH groups were made to point towards the position 5 of the quinoline ring they belong to, as in the models of 7b and 8b and in the structure of (1)_3_ (Fig. 6a). This conformation was termed 6b-T, T standing for “Three helix pattern”. In the other model, the OH groups were made to point towards the position 3 of the quinoline ring they belong to, as in the crystal structures of 2b and 3b. This conformation was termed 6b-S, S standing for “Shifted pattern”. Both models could be energy-minimized while preserving the orientation of the OH groups and the three inter-helix hydrogen bonds. In 6b-T, a view of the helix down its axis showed a 15-crown-5-like shape of the inner rim, suggesting that the helix held its preferred curvature of 2.5 units per turn (Fig. S19†). This was less obvious in 6b-S, hinting at a possible distortion of the main chain. Furthermore, the two helices had their axes parallel in 6b-T and at an angle in 6b-S. During MD simulation, the 6b-S was found to convert to 6b-T within 2 ns while the reverse process was not observed (Fig. S20†), suggesting a higher stability of 6b-T.

In summary, the evidence gathered so far suggests that T6f and T6r indeed mediate the helix-turn-helix arrangement desired to form three-helix bundles. This entails preventing the formation of aggregates and of other hydrogen-bonded arrangements that may occur in the absence of linker.^50^ The shorter helix-T6-helix models may also adopt a conformation with a different orientation of the hydrogen bonds without changing the donors and acceptors involved, but we expect these alternate conformations to be disfavored with the longer helices. Some differences were observed between T6f and T6r, with the more rigid T6r emerging as a better option to control conformation.

### Synthesis and folding of an abiotic unimolecular three-helix bundle

The synthetic approach to prepare 7a and 8a was a matter of strategic choices. While relatively long, these sequences should in principle still be well within the range of automated solid phase foldamer synthesis and may be prepared in one go, like 5a and 6a.^38^ However, our experience has shown that Reverse-Phase (RP) HPLC purification – the method that gave the best results so far – can be extremely challenging for long hydrophobic sequences. In turn, incomplete purification makes it difficult to assign multiple species seen in NMR spectra to impurities or to alternate conformers or aggregates. With their diethylene glycol side chains, the Q^D^ monomers help enhance hydrophilicity, and thus amenability to RP-HPLC, while not compromising solubility in organic solvent or crystal growth ability. Yet, the hydrophobic protecting groups of X̲ and Y̲ are unavoidable. We therefore opted for a fragment condensation approach to synthesize 7a and 8a. This entailed the preparation and careful RP-HPLC purification of relatively short fragments, and their subsequent coupling on solid phase. In the final mixture, deleted products where an entire fragment is missing should differ in size from the product sufficiently to allow for purification by recycling gel permeation chromatography (GPC) in chloroform.

**Fig. 7:**
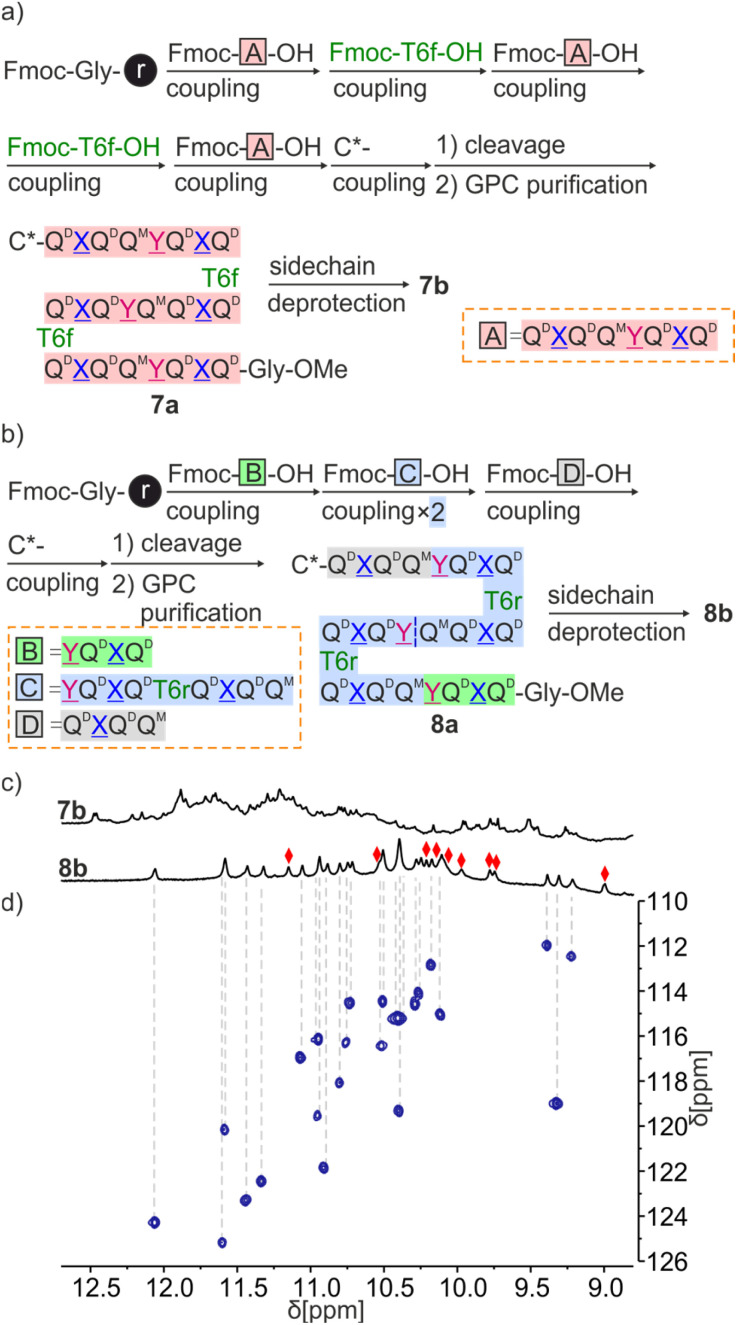
Scheme representation of fragment condensation strategies for the synthesis of 7a, 7b (a) and 8a, 8b (b). (c) Extracts of ^1^H NMR spectra (500 MHz, CD_2_Cl_2_) of 7b and 8b. (d) Extract of ^1^H,^15^N HSQC (500 MHz, CD_2_Cl_2_) of 8b. The region above 8.8 ppm is shown, see Fig. S25† for a full spectrum. Only NH resonances correlate, red diamonds indicate the signals of hydrogen-bonded OH protons.

Sequences 7a and 8a both have three identical octaamide segments linked by T6f or T6r turns. However, fragment condensation with aromatic helices works much better on aliphatic amines and this led to adopting different strategies for the two targets (Fig. 7). T6f is terminated by an aliphatic amine where segments can be coupled. Accordingly, we prepared the Fmoc-QX̲QQY̲QX̲Q-OH repeat motif (fragment A in Fig. 7a) on SASRIN™ resin and purified it in its fully protected form. Sequence 7a was then assembled on a Gly-HMBA AM resin using alternatively fragment A activated with BOP and T6f activated as an acid chloride. The terminal Fmoc group was removed with 2% DBU/NMP and (1*S*)-camphanic chloride was used to finally cap the N terminus and induce a *P* handedness in the N terminal helix. After cleavage from the resin as a methyl ester using the same conditions as for 5a and 6a, 7a was purified by GPC and isolated in high purity in an overall yield of 10%.

Because T6r is terminated by an aromatic amine, a different strategy was required to prepare 8a. The sequence was divided into four fragments of three different kinds (B, C and D in Fig. 7b) with the plan to condense the fragments at the benzylic amine of Y̲ units. The synthesis proceeded as for 7a with the prior synthesis of the fragments on SASRIN™ resin and their purification as a free carboxylic acid, followed by their assembly on Gly-HMBA AM resin. The final isolated yield after GPC purification was 9%.

Consistent with the behavior of 5a, the ^1^H NMR spectrum of 7a showed four sets of signals with similar intensities (ratio of 1/0.9/0.85/0.8) that can be explained by the coexistence of *PPP*, *PMP*, *PPM* and *PMM* diastereomeric conformers (Fig. S5†). In contrast, and in agreement with the behavior of 6a, the ^1^H NMR spectrum of 8a showed one set of signals assigned to the *PPP* conformer (Fig. S5†). After side chain deprotection, the ^1^H NMR spectra of 7b in both CDCl_3_ and CD_2_Cl_2_ showed numerous overlapping signals indicating the presence of several species (Fig. 7c and S21†). No significant changes were observed after allowing the solution to stand for 2 weeks (Fig. S22†). It is not clear whether the species correspond to aggregates, severely kinetically trapped states, alternate folds or a combination of these. Adding DMSO-*d*_6_ to a solution of 7b to disrupt the hydrogen bonds led to a simplification of the spectrum (Fig. S23†).

**Fig. 8:**
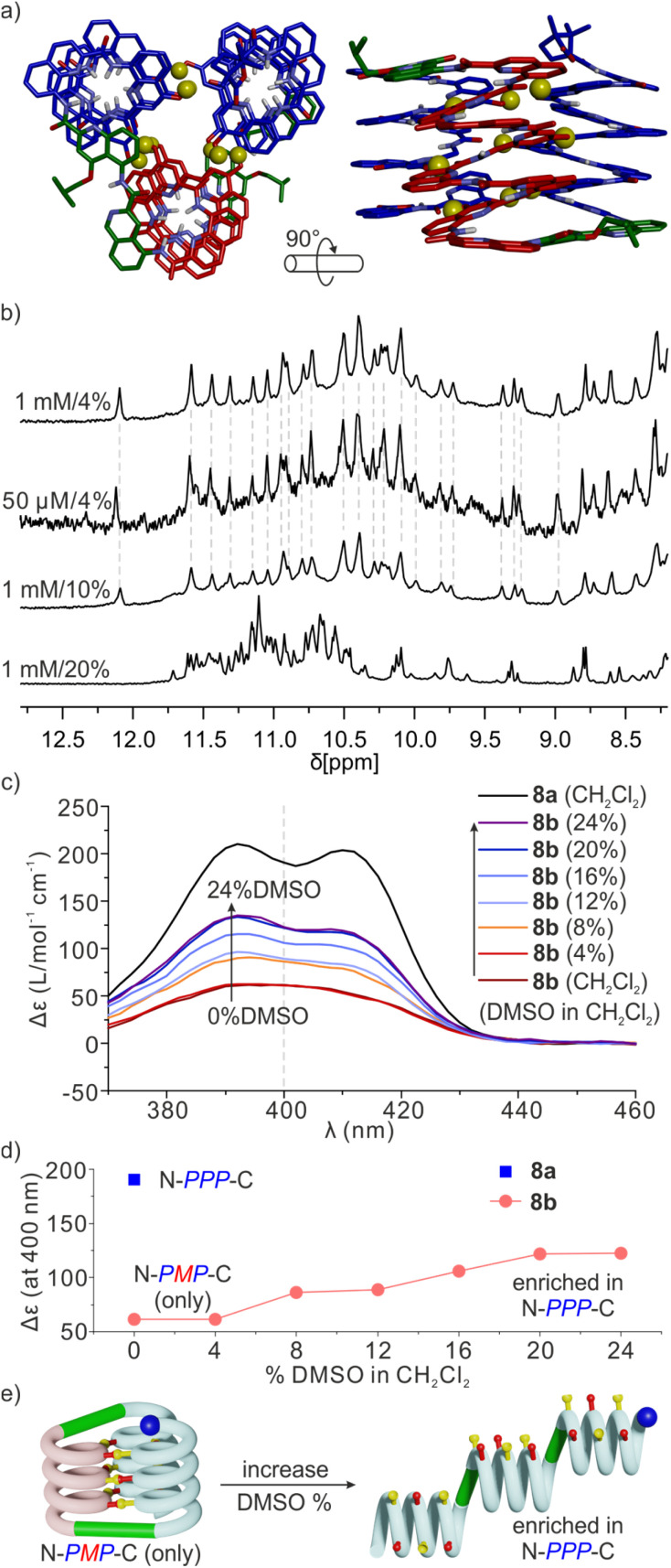
(a) Molecular model of 8b. The molecule is shown in stick representation. Non-polar hydrogen atoms and side chains (except that of the T6r turn) have been removed for clarity. Hydroxy protons are shown as yellow balls. (b) Excerpts of ^1^H NMR spectra (500 MHz, CD_2_Cl_2_) of 8b in DMSO-*d*_6_/CD_2_Cl_2_ at different concentrations and different vol% of DMSO-*d*_6_. (c) CD spectra of 8a and 8b, 50 μM in CH_2_Cl_2_ (DCM) with different proportions of DMSO. (d) Δ*ε* values at 400 nm extracted from (c) as a function of the vol% of DMSO in CH_2_Cl_2_. (e) Schematic representation of the process of hydrogen bond and tertiary fold disruption.

Sequence 8b behaved differently from 7b. Its spectrum in CD_2_Cl_2_ showed one set of signals indicating a well-defined unimolecular species or a symmetrical aggregate (Fig. 7c and S24†), while the spectrum in CDCl_3_ was complex, reflecting the coexistence of several conformers or aggregates in solution. The ^1^H,^15^N HSQC spectrum in CD_2_Cl_2_ allowed us to assign all twenty-nine ^15^NH̲ resonances and, indirectly, the nine hydrogen bonded O*H* resonances (Fig. 7d and S25†). The DOSY spectrum of an 8a + 8b mixture in CD_2_Cl_2_ confirms the smaller size of 8b and thus its probable monomeric nature (Fig. S26†). Moreover, the ^1^H NMR spectrum did not significantly change upon adding 4% DMSO-*d*_6_ even at concentrations as low as 50 μmol (Fig. 8b and S27†). This proportion of DMSO is usually insufficient to disrupt tertiary folds but potentially destabilizing for some aggregates.^50,53,54^ The effect of further increasing the proportion of DMSO was monitored by ^1^H NMR and CD. The ^1^H NMR spectra showed that a transition occurred between 12 and 16% of DMSO (Fig. S28†), that is, more than required for the disruption of the folded conformer of 6b, hinting at a higher stability of 8b. The CD spectra corroborated our interpretation of NMR data. The spectrum of protected precursor 8a showed a strong positive band consistent with a *PPP* conformation (Fig. 8c). In comparison, the CD spectrum of 8b showed a relatively weak band in CH_2_Cl_2_ which we assigned to the desired *PMP* conformer in which the contributions of the helical segments partly cancel each other. As described above for 4b, the intensity of this band increased upon adding DMSO as the *PMP* conformer is at least in part disrupted, leading to an increase of the proportion of *PPP* conformer (Fig. 8c–e).

Taken altogether, these data point to 8b being a unimolecular tertiary fold with two helix handedness reversals held together by nine intramolecular hydrogen bonds consistent with the initially proposed molecular model (Fig. 8a). Well-defined folding of 8b, however, appears to be restricted to CH_2_Cl_2_. In CDCl_3_, its ^1^H NMR spectrum is broad and shows multiple species. These species already emerge upon adding CDCl_3_ to a solution of 8b in CD_2_Cl_2_ (Fig. S24†). Whether these species correspond to alternate folds or aggregates is unknown at this stage. One may point to other examples that we previously reported of changes in aggregation behavior of aromatic helices between CDCl_3_ and CD_2_Cl_2_.^69^ It is probably because of other possibilities these molecules have to fold and aggregate that the rigid T6r turn brought a critical advantage to control the conformation of 8b in CD_2_Cl_2_. Indeed, the *PMP* three-helix bundle of 8b does not form at all in the absence of linker – sequence 1 trimerizes in a *PPP*/*MMM* three-helix bundle. Apparently, the more flexible T6f turn of 7b failed to bring a sufficient advantage. One important lesson to draw from this study is thus that the design of a well-defined tertiary fold amounts as much to stabilizing the desired fold as to preventing the formation of alternate structures.

## Conclusions

Starting from a well-characterized *C*_3_-symmetrical *PPP*/*MMM* parallel trimolecular bundle of three aromatic helices held together by intermolecular hydrogen bonds, we have used simple rational principles and molecular modelling to design a similar *PMP* structure where the central helix had an opposite orientation and handedness. We next identified possible linkers to connect the termini of the helices to transform a trimolecular bundle into a unimolecular tertiary fold in which each helix hydrogen bonds to the two others. The synthesis and characterization first of reduced models and then of the full size 6.9 kDa abiotic fold validated the overall approach. The final unimolecular three-helix bundle was shown to be stable in CH_2_Cl_2_ even though other solvents or the use of flexible linkers impacted its behavior. These results represent an important milestone in the exploration of abiotic tertiary foldamers. The whole process that led to the unimolecular three-helix bundle highlights well what can be designed and how simple molecular modelling tools work quite effectively. The solvent dependence suggests that the design of 8b can be further improved to enhance its robustness. Progress in this direction is being made and will be reported in due course.

## Data availability

Crystallographic data for 2b and 3b has been deposited at the CCDC under accession numbers 2391393 and 2391429, respectively, and can be obtained from https://www.ccdc.cam.ac.uk.

## Author contributions

SW performed the syntheses. JS collected diffraction data and solved the solid-state structures. SW and LA performed solution studies. SW and VM performed modelling studies. IH supervised the research. SW and IH wrote the manuscript. All authors proofread and improved the manuscript.

## Conflicts of interest

There are no conflicts to declare.

## Supplementary Material

SC-OLF-D4SC07336C-s001

SC-OLF-D4SC07336C-s002
