# Supplementary material for: Design of an abiotic unimolecular three-helix bundle
Source: Chem Sci. 2024 Dec 4;16(3):1136–46. doi: 10.1039/d4sc07336c (PMC11615733; doi:10.1039/d4sc07336c)
Supplement: SC-016-D4SC07336C-s001 [file SC-016-D4SC07336C-s001.pdf]

Supplementary Information (SI) for Chemical Science.
This journal is © The Royal Society of Chemistry 2024

Supplementary Information

for:

Design of an abiotic unimolecular three-helix bundle

Shuhe Wang, Johannes Sigl, Lars Allmendinger, Victor Maurizot and Ivan Huc

Contents

1 List of Abbreviations 2
2 Supplementary figures 3
3 Supplementary methods. 21
3.1 LC-MS analyses 21
3.2 Molecular modelling 21
3.3 Molecular dynamic simulations 21
3.4 Nuclear magnetic resonance spectroscopy 22
3.5 CD studies 23
3.6 X-ray crystallography 23
4 Synthetic Schemes 26
4.1 Synthesis of turn units 26
4.2 synthesis of foldamers 28
5 Synthetic Procedures 33
5.1 General methods 33
5.2 synthesis of monomers and the turn units. 33
5.3 Solid phase synthesis general methods 39
5.3.1 Loading of the resin via HBTU activation 39
5.3.2 Estimation of the loading 39
5.3.3 Solid phase synthesis of monomer and turn units via *in-situ* activation 39
5.3.4 Fragment condensation via BOP activation 40
5.3.5 Mini-cleavage 40
5.3.6 Full cleavage 41
5.4 Synthesis of oligomers 41
6 References 48
7 NMR spectra of new compounds 49
8 MS spectra of 7a, 7b, 8a and 8b 72

1 List of Abbreviations

**CD**	circular dichroism
**DBU**	1,8-diazabicyclo[5.4.0]undec-7-ene
**DCM**	dichloromethane
**DIPEA**	*N*,*N*-diisopropylethylamine
**DMF**	*N*,*N*-dimethylformamide
**DMSO**	dimethyl sulfoxide
**DOSY**	diffusion ordered spectroscopy
**HR-ESI**	high resolution electrospray ionization
**eq.**	equivalent
**Fmoc**	fluorenylmethoxycarbonyl
**HBTU**	2-(1*H*-benzotriazol-1-yl)-1,1,3,3-tetramethyluronium hexafluorophosphate
**HSQC**	heteronuclear single quantum correlation
**Me**	methyl
**MeOH**	methanol
**min**	minutes
**MS**	mass spectrometry
**MW**	microwave
**NMP**	*N*-methyl-2-pyrrolidone
**NMR**	nuclear magnetic resonance
**r. t.**	room temperature
**SPS**	solid phase synthesis
***t*Bu**	*tert*-butyl
**TFA**	trifluoroacetic acid
**THF**	tetrahydrofuran
**UV/Vis**	ultraviolet–visible

2 Supplementary figures

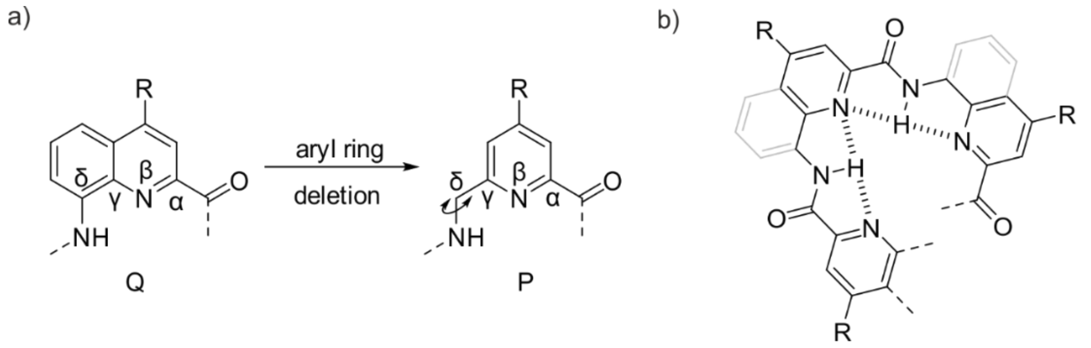

Figure S1. Chemical structure of Q and P units and the folding principle of their oligomers. a) Chemical structure of Q (or X with R = OH) and P (or Y with R = OH). b) Intramolecular H-bonding and helical folding principle of P/Q oligomers. Note that the amide carbonyl groups diverge from the folded structures and thus provide hydrogen bond acceptors.

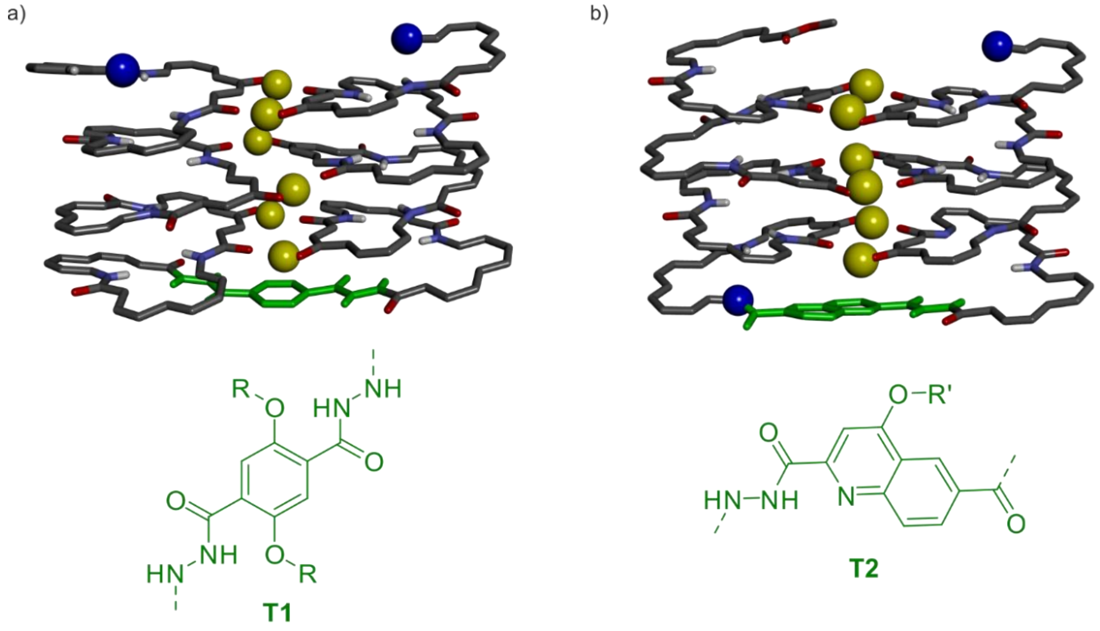

Figure S2. a) The side view of a helix-T1-helix tertiary structure and the chemical structure of T1.[1] **b) The side view of a helix-T2-helix tertiary structure and the chemical structure of T2.**[2] The N-terminus of each helix is highlighted with a blue ball. The hydroxyl proton hydrogen bond donors are shown as yellow balls. Turn units are coloured in green. Only the outer rim of the helix is shown. The side chains on the Q unit and turn unit are omitted for clarity.

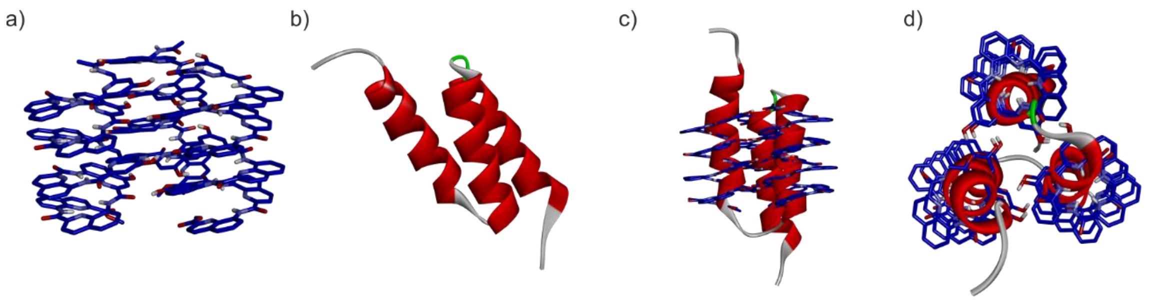

Figure S3. Comparison of an abiotic three helix bundle and the Z domain derived from protein A. a) The structure of $(\mathbf{1})_3$[1]. b) A modified subdomain of protein A (b, PDB:2B89). The side view (c) and top view (d) of the overlay of the structures shown in (a) and (b).

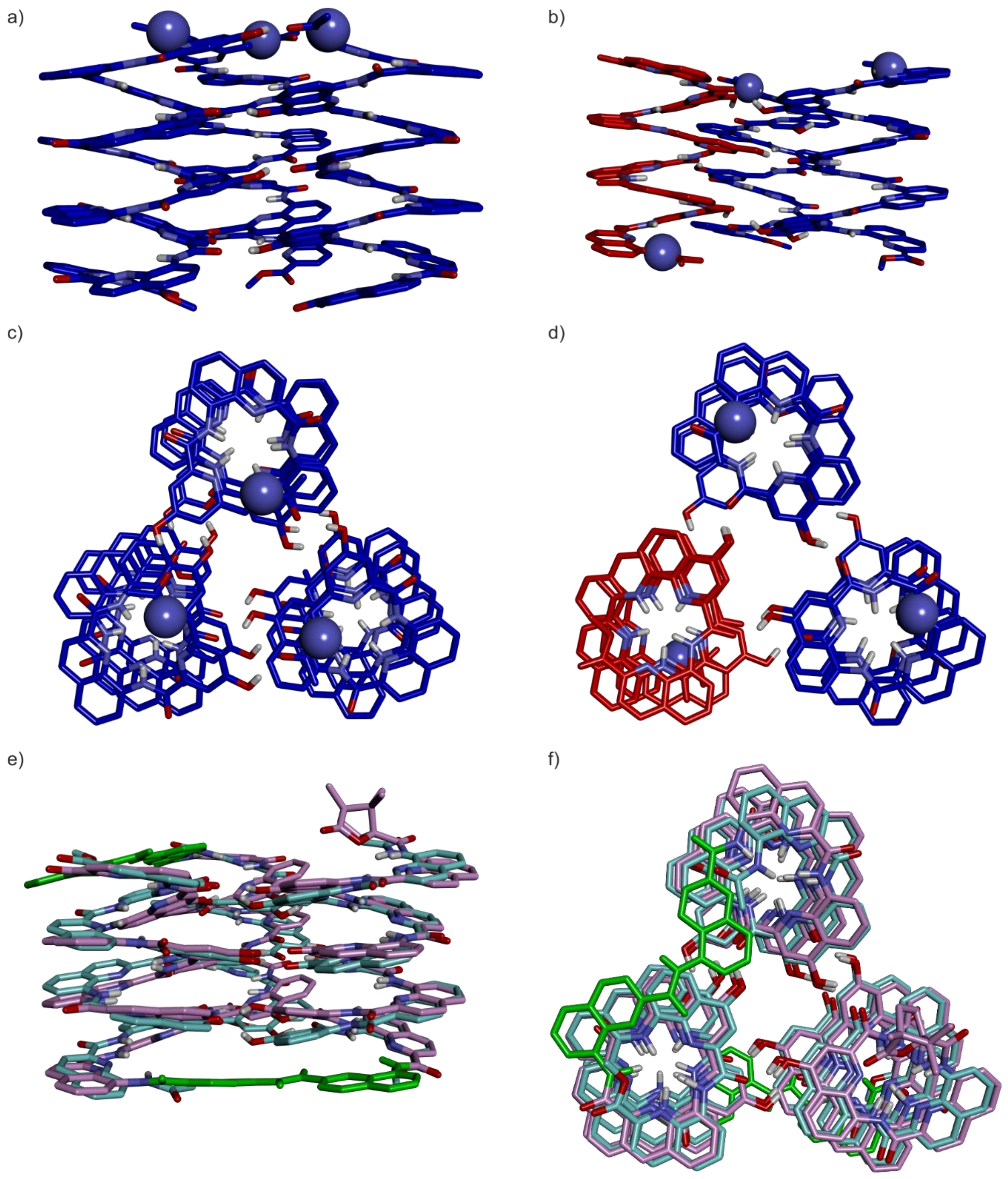

Figure S4. Design of the unimolecular three helix bundle and of the turn units. The side view (a) and top view (c) of the crystal structure of (**1**)$_3$.[1] The side view (b) and top view (d) of an energy-minimised heterochiral trimer molecular model of sequence (Ac-QXQQYQXQ-OMe)$_3$. The *P* helices are coloured in blue and *M* helices are coloured in red. The N-termini are shown as blue balls. Side chains are omitted for clarity. Side view (e) and top view (f) of the overlay of the structure in (b) and of the energy-minimised model of sequence **8b**. The (Ac-QXQQYQXQ-OMe)$_3$ is coloured in cyan and sequence **8b** is coloured in pink (helical segments) and green (T6r turn unit).

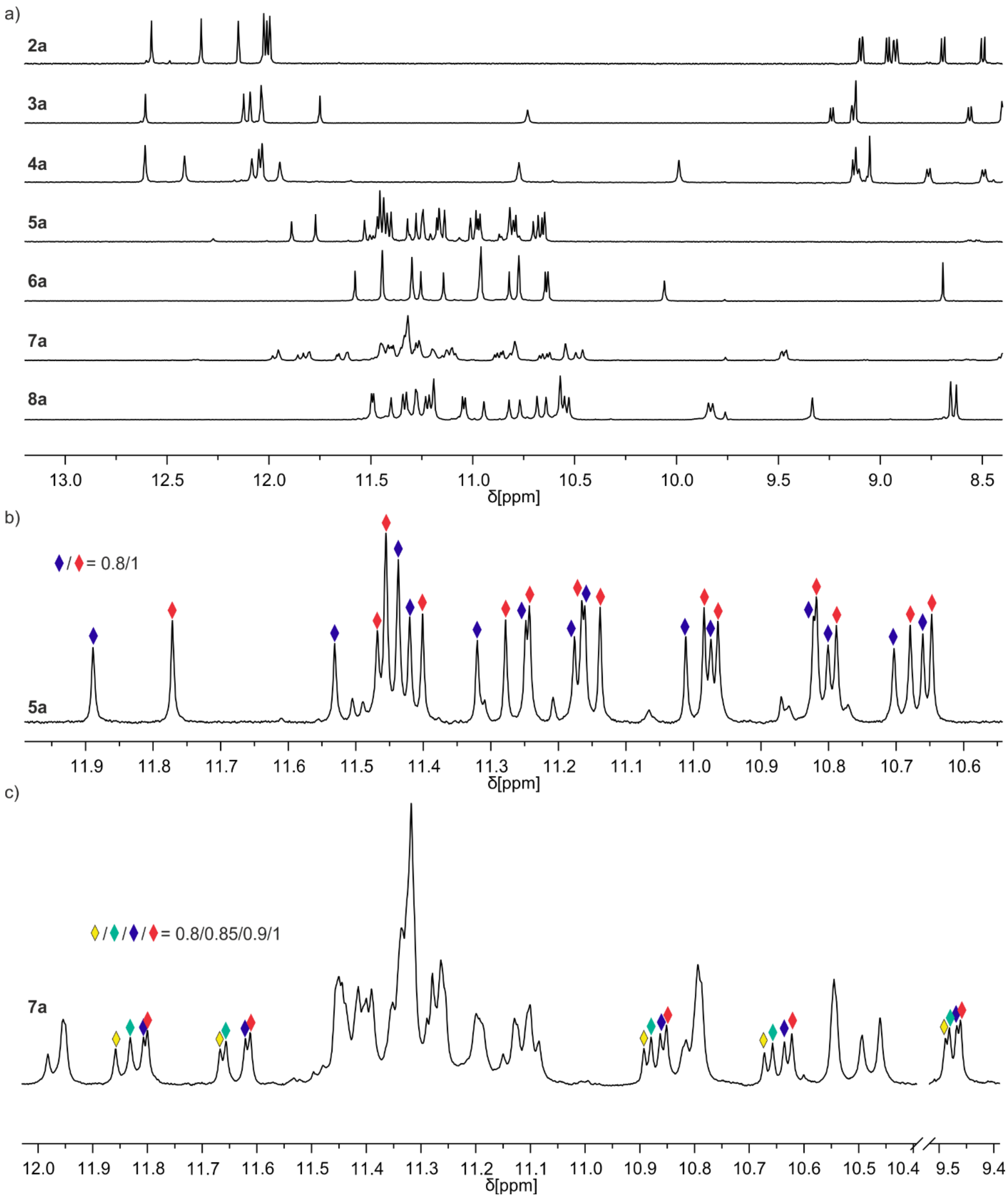

Figure S5. ^{1}H NMR spectra of side chain protected sequences. a) Extracts of the ^{1}H NMR spectra (500 MHz, $CDCl_3$) of **2a**, **3a**, **4a**, **5a**, **6a**, **7a** and **8a**. The sequences **2a**, **3a** and **4a** show one set of signals because the equilibrium between their diastereomeric conformers is fast on the NMR timescale, not necessarily because only one species is present. Sequence **5a** shows two sets of signals from two major species which were assigned to *PP*/*MM* and *PM*/*MP* conformers. Sequence **6a** shows one major species assigned to the *PP*/*MM* conformer. Sequence **7a** shows four sets of signals assigned to *PPP*, *PMP*, *PPM* and *PMM* conformers. Sequence **8a** show one major species assigned to the *PPP* conformer. The enlarged spectra of **5a** (b) and **7a** (c) show the presence of different isomers and their proportions.

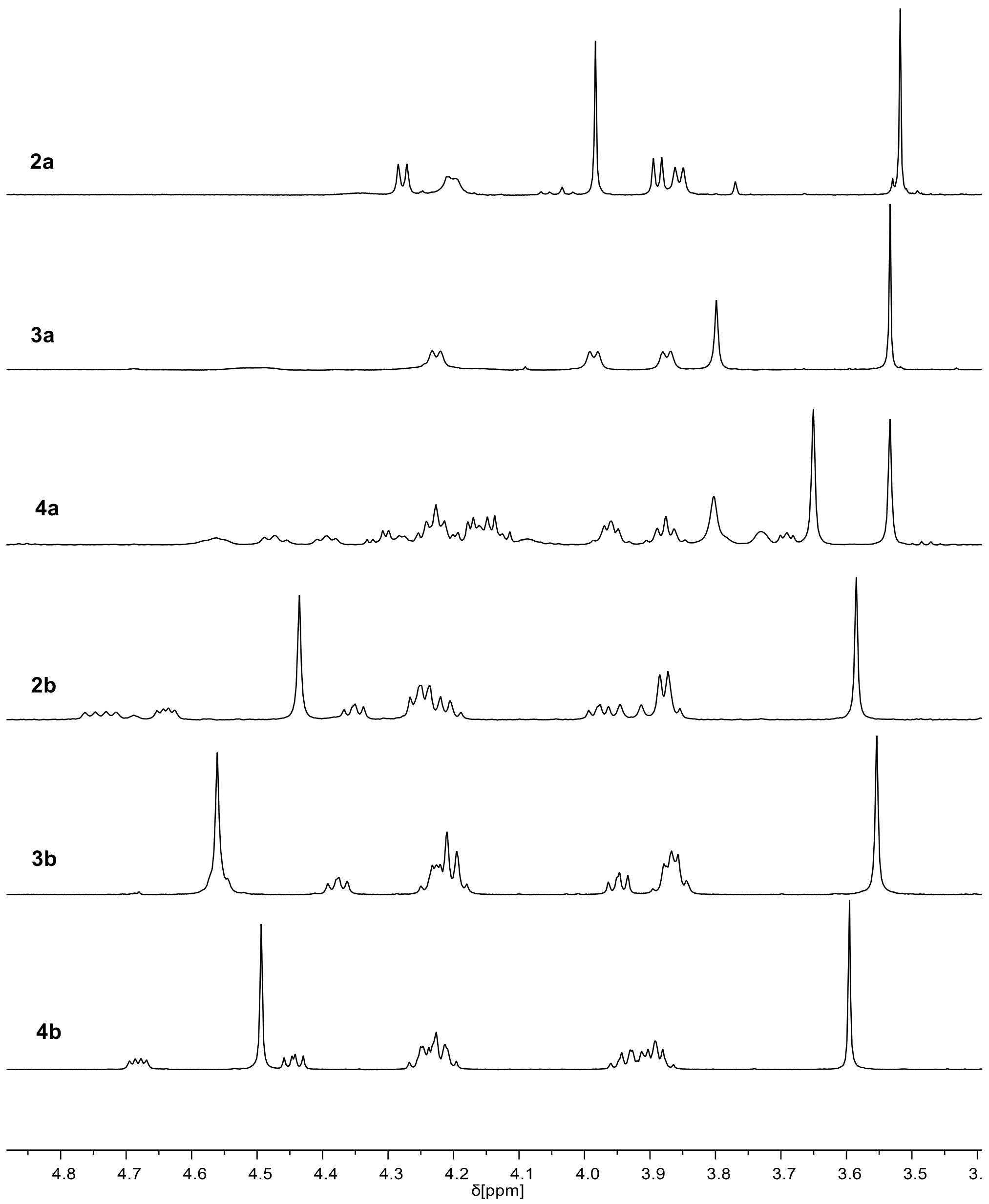

Figure S6. Excerpts of the ^{1}H NMR spectra (500 MHz, $CDCl_3$) of 2a, **3a**, **4a**, **2b**, **3b and 4b.** The singlet near 3.6 ppm is the methyl ester resonance. The other singlet belongs to the Q^M methoxy side chain. The deshielding of the Q^M methyl group in **2b**, **3b**, and **4b** is consistent with its proximity to the carbonyl group of the X unit on the other helix, as seen in the solid state structures of **2b** and **3b**. All other resonances belong to the diastereotopic CH_2 groups of *i*Bu side chains and T6f units. For **2a** and **3a**, CH_2 groups appear as doublets indicating fast exchange between *PP*/*MM* and *PM*/MP conformers at 298 K. Note that in both cases, one CH_2 group is broadened (coalesced) to such an extent that it is not visible. In **4b**, the CH_2 groups appear as doublets of doublets of diastereotopic protons. This is a consequence of this molecule being chiral while exchange is still fast on the NMR timescale. In contrast, the doublet of doublets in the spectra of achiral **2b** and **3b** indicate slow exchange on the NMR time scale. In the slow exchange regime, a single set of signals indicates that a single, racemic, diastereomeric conformer is present.

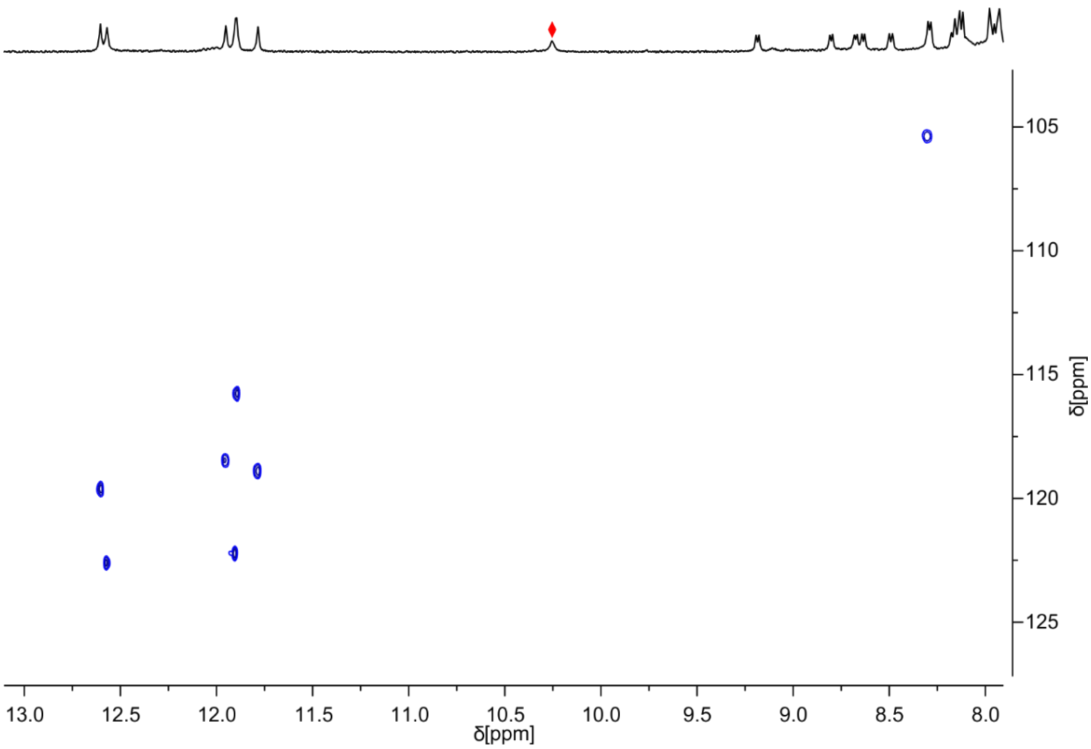

Figure S7. Indirect identification of the signals of the hydrogen-bonded OH proton of 2b. Extract of the $^{1}H,^{15}N$ HSQC NMR spectrum (500 MHz, $CDCl_3$) of **2b**. Only NH resonances correlate, the red diamond indicates the signal of the hydrogen bonded OH proton.

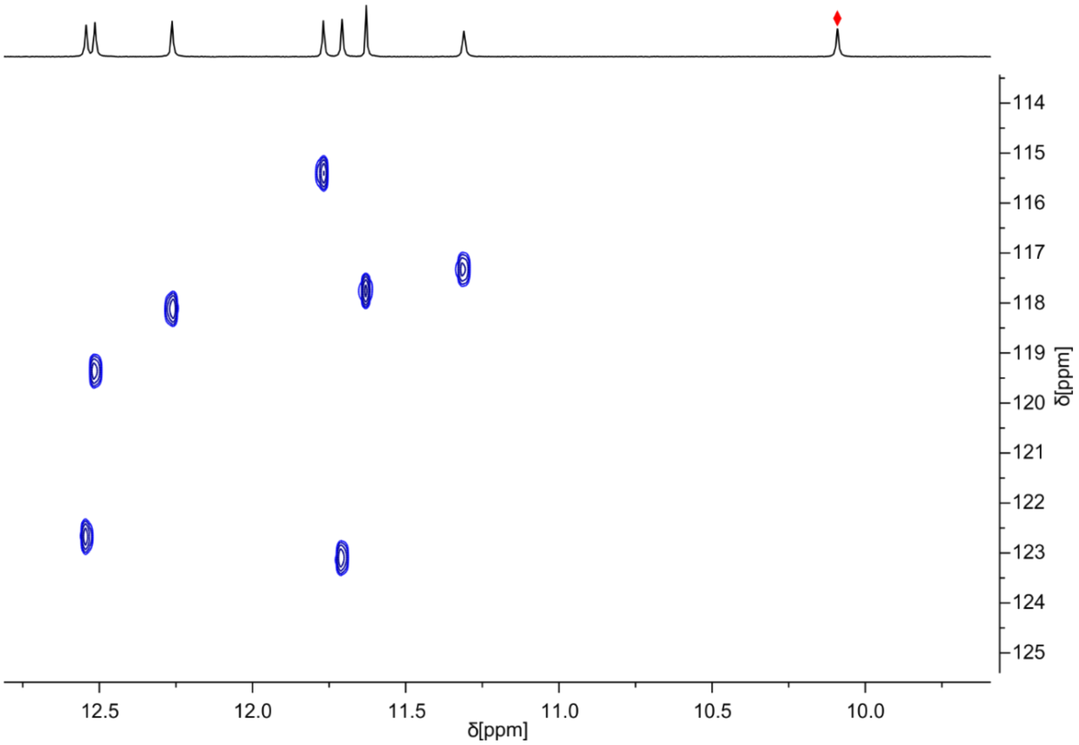

Figure S8. Indirect identification of the signals of hydrogen-bonded OH proton of 3b. Extract of the $^{1}H,^{15}N$ HSQC NMR spectrum (500 MHz, $CDCl_3$) of **3b**. Only NH resonances correlate, the red diamond indicates the signal of the hydrogen bonded OH proton.

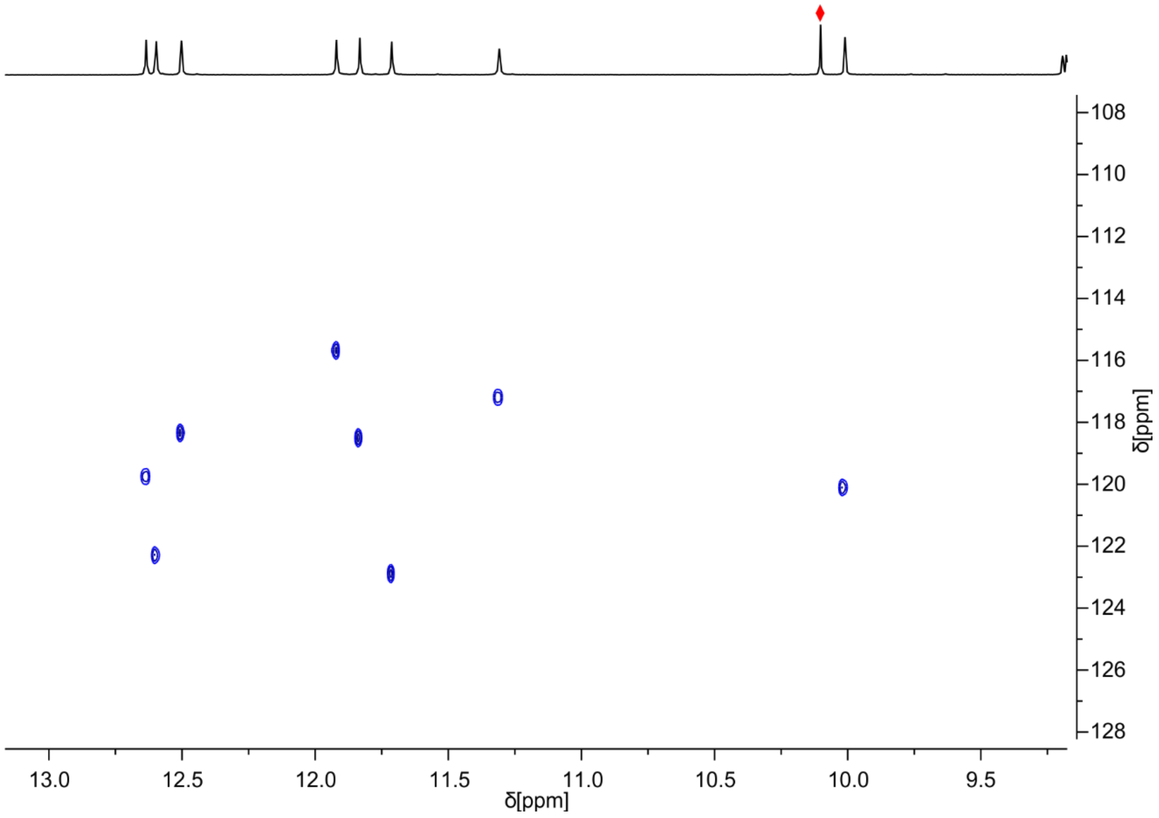

Figure S9. Indirect identification of the signals of hydrogen-bonded OH proton of 4b. Extract of the $^1H,^{15}N$ HSQC NMR spectrum (500 MHz, $CDCl_3$) of **4b**. Only NH resonances correlate, the red diamond indicates the signal of the hydrogen bonded OH proton.

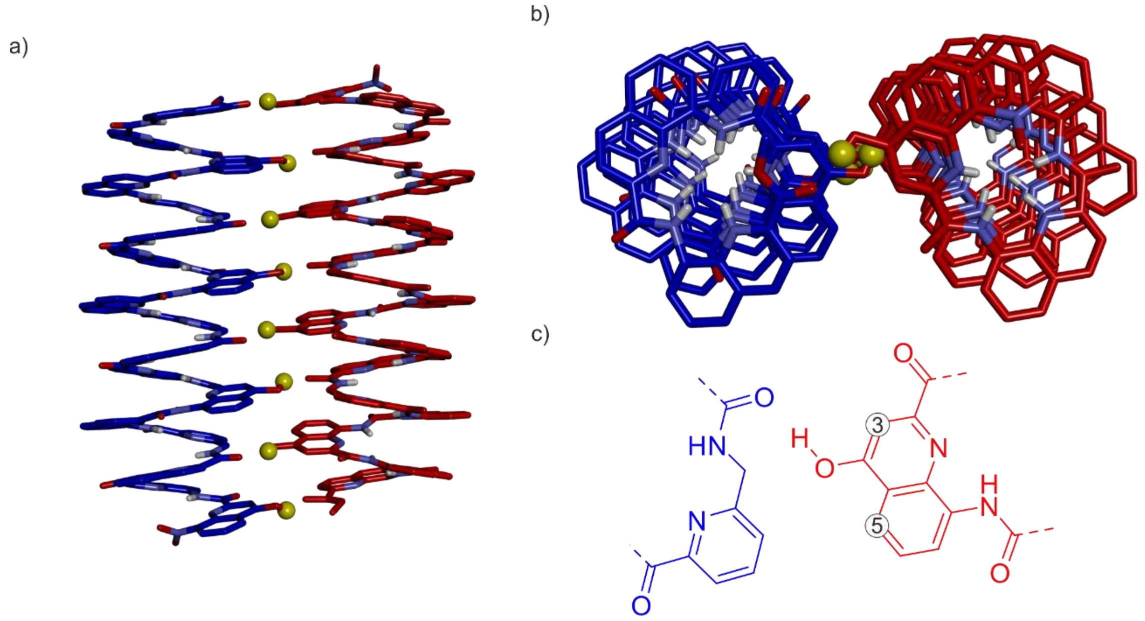

Figure S10. The crystal structure of a shifted dimer.[3] The side view (a) and top view (b) of the shifted dimer structure. The backbones of *P* and *M* helices are coloured blue and red, respectively. c) The intermolecular hydrogen bond pattern.

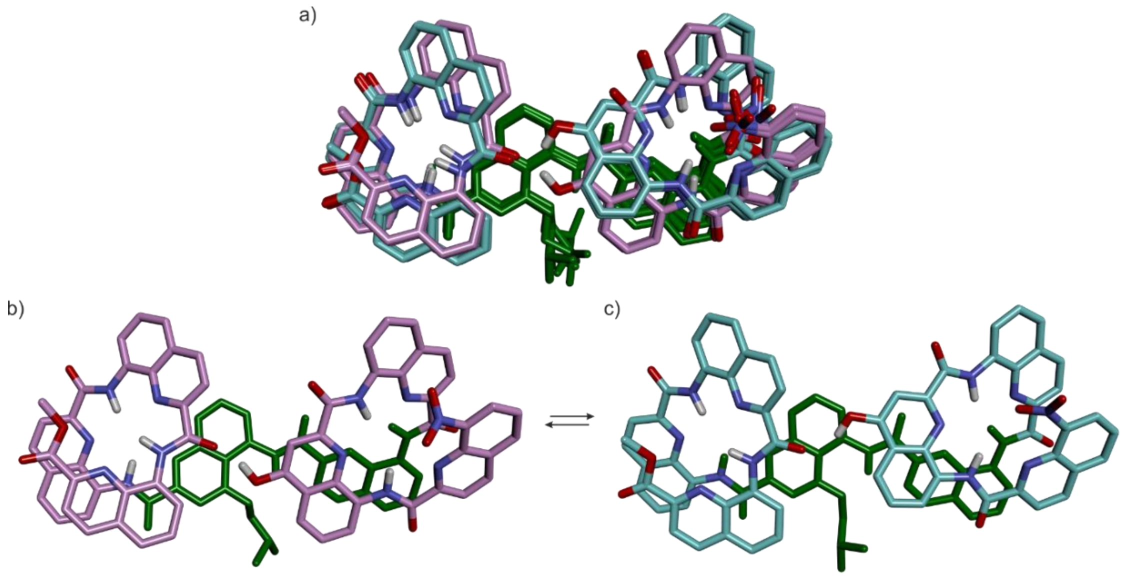

Figure S11. Molecular dynamic (MD) simulation of 3b. The MD simulation was performed based on the crystal structure of **3b** in Maestro (MMFFs, $CHCl_3$, TNCG, 300 K, 1 ns). a) The overlay of ten structures sampled during the MD simulation. b) The crystal structure of **3b**. c) One helix-turn-helix structure mediated by the trimer pattern extracted from the MD simulation. The two patterns interconvert rapidly.

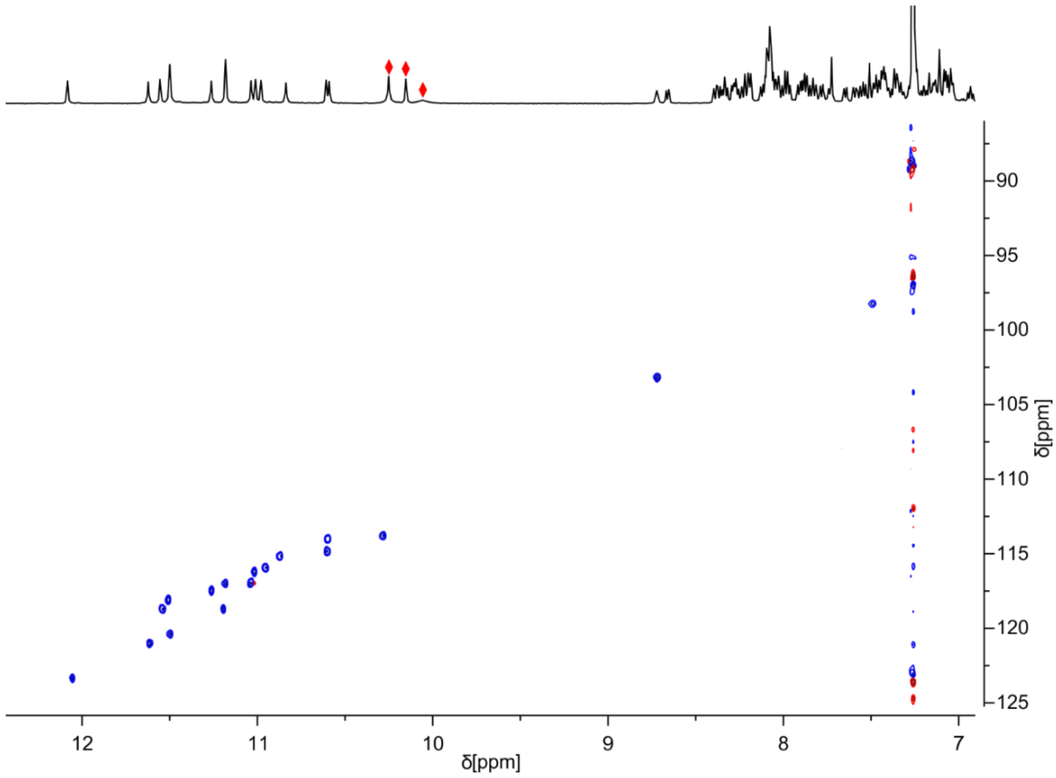

Figure S12. **Indirect identification of the signals of hydrogen-bonded OH protons of 5b in $CDCl_3$**. Extract of the $^1H,^{15}N$ HSQC NMR spectrum (500 MHz, $CDCl_3$) of **5b**. Only NH resonances correlate, red diamonds indicate the signals of hydrogen bonded OH protons. The correlation of the glycine NH signal is probably hidden under the streak of the t1 noise caused by the intense resonance of $CHCl_3$.

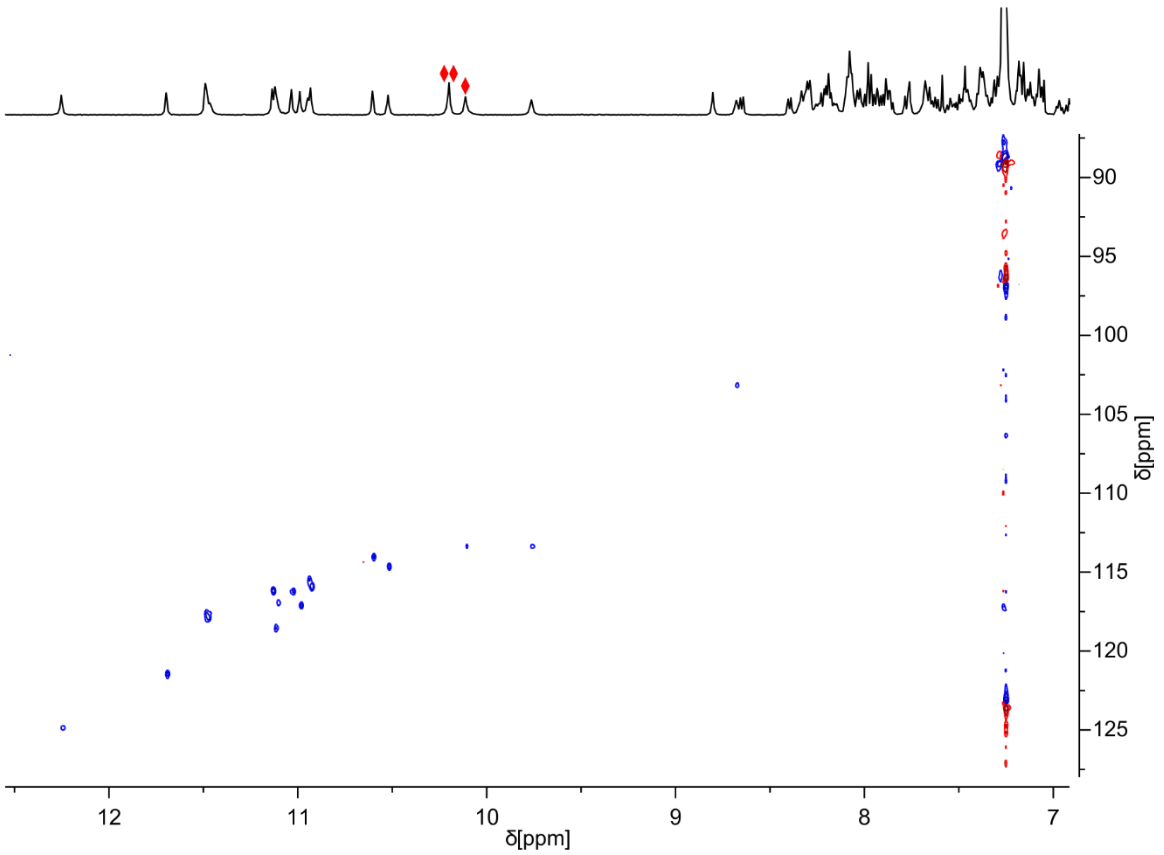

Figure S13. Indirect identification of the signals of hydrogen-bonded OH protons of 6b in $CDCl_3$. Extract of the $^{1}H,^{15}N$ HSQC NMR spectrum (500 MHz, $CDCl_3$) of **6b**. Only NH resonances correlate, red diamonds indicate the signals of hydrogen-bonded OH protons. The correlation of the glycine NH signal is probably hidden under the streak of the t1 noise caused by the intense resonance of $CHCl_3$.

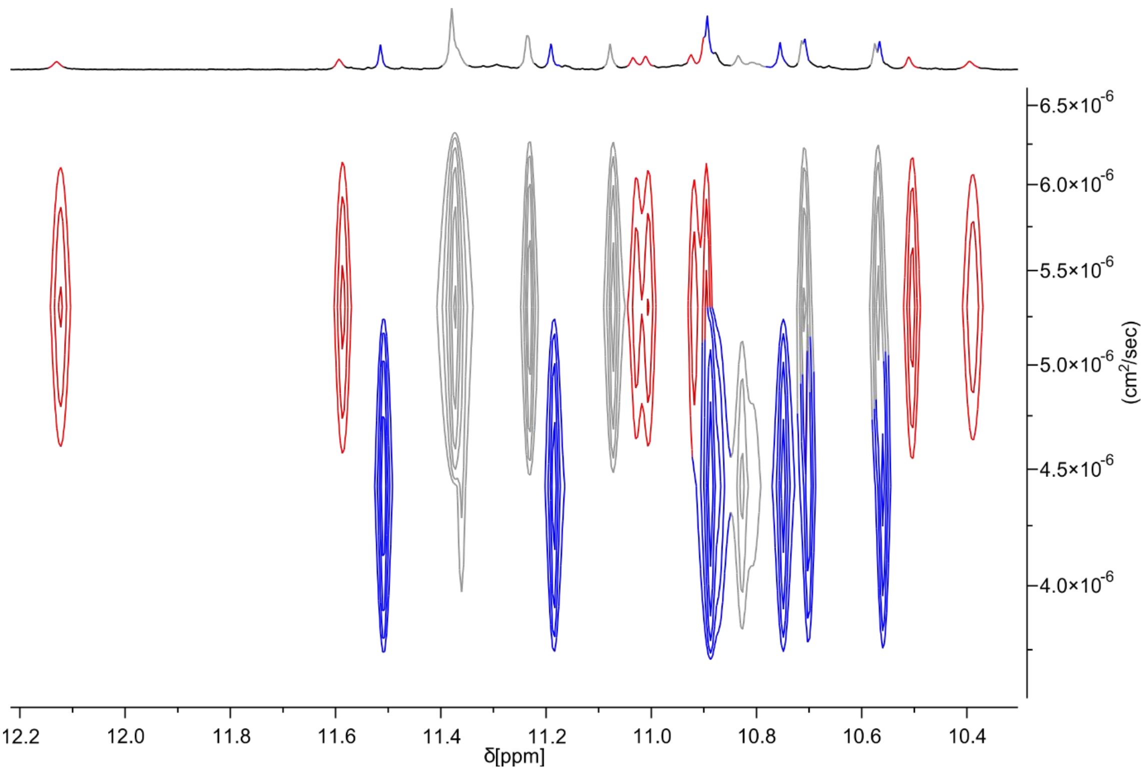

Figure S14. ^{1}H DOSY spectrum (500 MHz, $CDCl_3$) of a mixture of 6a and 6b with a ratio of 1/0.8. Some peaks assigned to **6a** are highlighted in blue and those assigned to **6b** are highlighted in red. Protected sequence **6a** is monomeric and its diffusion coefficient is substantially lower than that of **6b**, indicating that the latter is also monomeric and supporting a compact helix-turn-helix conformation of **6b**.

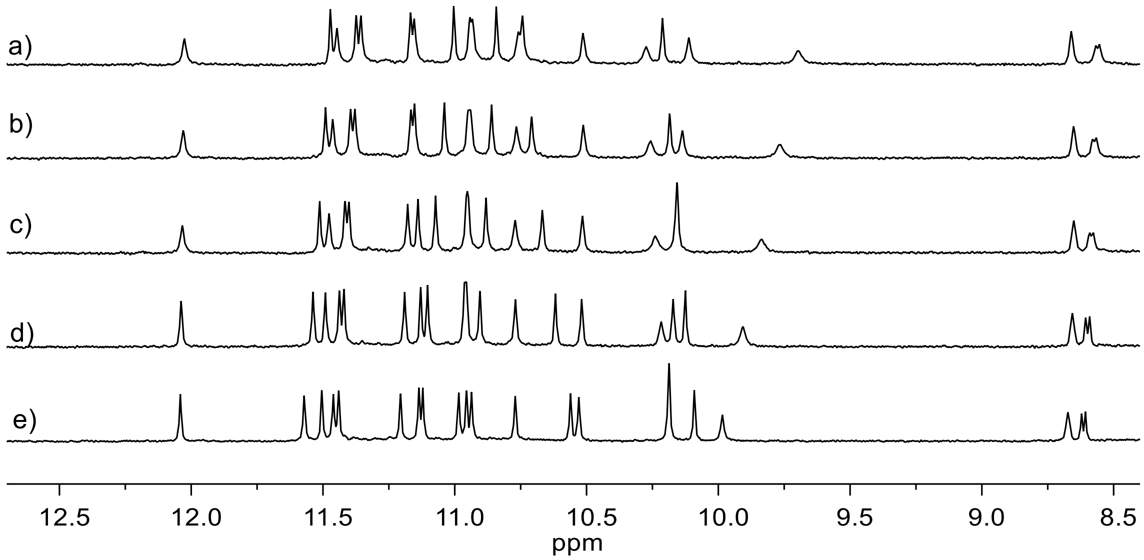

Figure S15. ^{1}H NMR spectra of 5b in $CDCl_3$/CD_2Cl_2 mixtures. Extracts of the ^{1}H NMR spectra (500 MHz) of **5b** in $CDCl_3$/CD_2Cl_2 mixtures showing the amide and hydroxy proton resonances. The vol% of CD_2Cl_2 are 100 (a), 75 (b), 50 (c), 25 (d), and 0 (e). In other sequences, it was observed that the aggregation behaviour or the conformation change radically with $CDCl_3$ or CD_2Cl_2,[1,3] but it is not the case for **5b**.

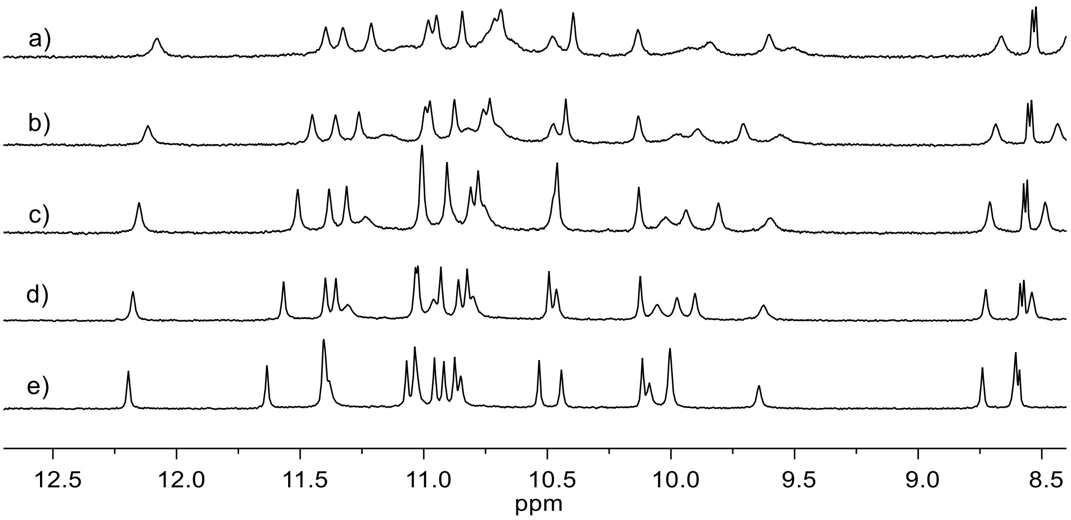

Figure S16. ^{1}H NMR spectra of 6b in $CDCl_3/CD_2Cl_2$ mixtures. Extracts of the ^{1}H NMR spectra (500 MHz) of **6b** in $CDCl_3/CD_2Cl_2$ mixtures showing the amide and hydroxy proton resonances. The vol% of CD_2Cl_2 are 100 (a), 75 (b), 50 (c), 25 (d), and 0 (e). In other sequences, it was observed that the aggregation behaviour or the conformation change radically with $CDCl_3$ or CD_2Cl_2,[1,3] but it is not the case for **6b**.

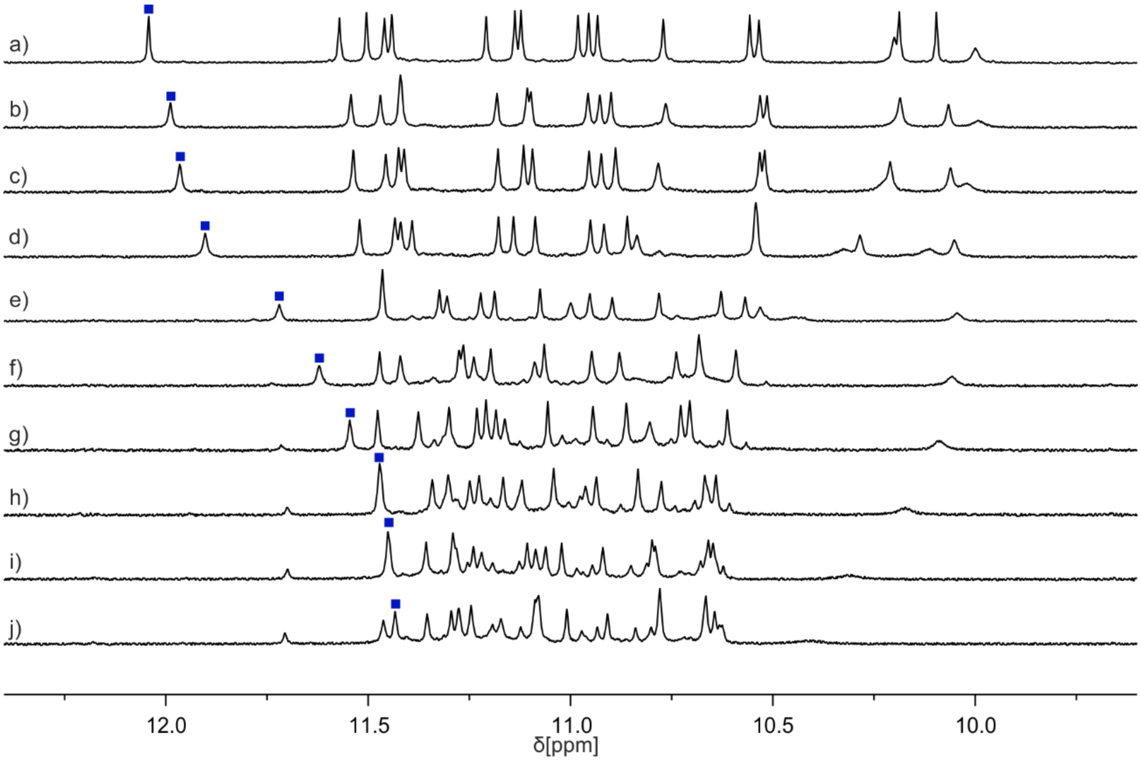

Figure S17. ^{1}H NMR spectra of 5b in DMSO-d_6/$CDCl_3$ mixtures. Extracts of the ^{1}H NMR spectra (500 MHz, 298K) of **5b** in DMSO-d_6/$CDCl_3$ mixtures. The vol% of DMSO-d_6 are 0 (a), 1 (b), 2 (c), 4 (d), 8 (e), 10 (f), 12 (g), 16 (h), 20 (i), and 24 (j). The variation of the chemical shift of the signal marked with a blue box is shown in Figure 6c.

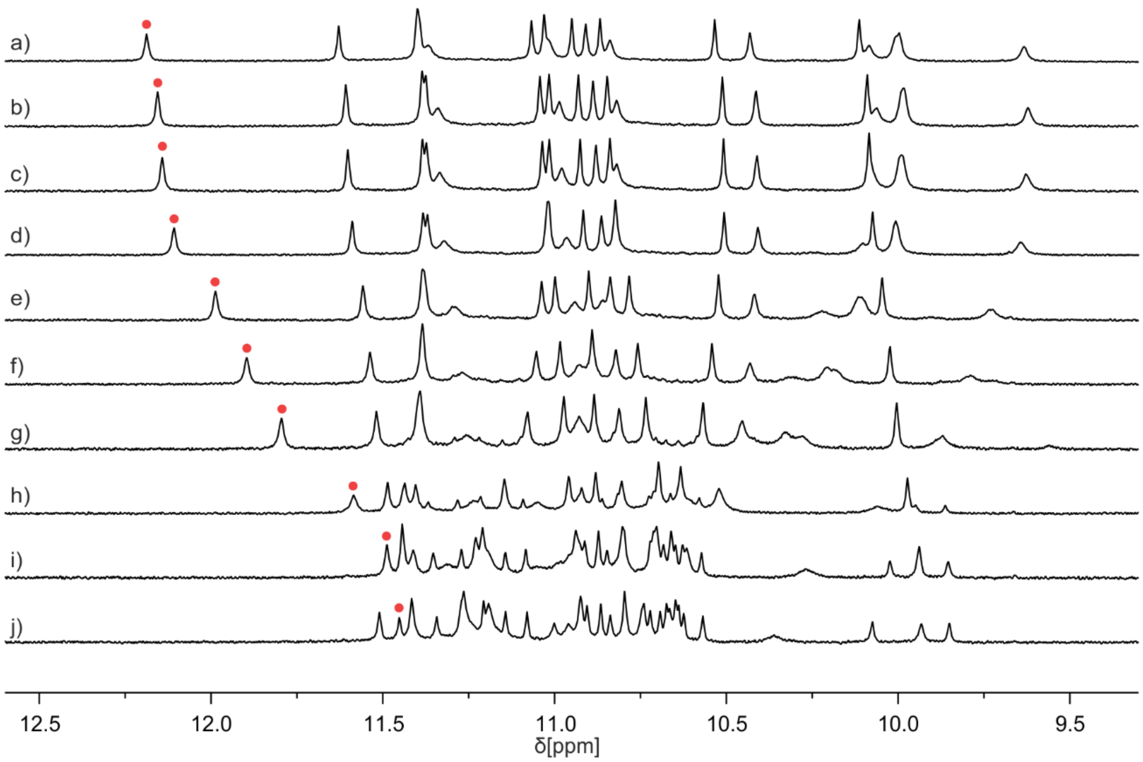

Figure S18. ^{1}H NMR spectra of 6b in DMSO-*d*$_6$/CDCl$_3$ mixtures. Extracts of the ^{1}H NMR spectra (500 MHz, 298K) of **6b** in DMSO-*d*$_6$/CDCl$_3$ mixtures. The vol% of DMSO-*d*$_6$ are 0 (a), 1 (b), 2 (c), 4 (d), 8 (e), 10 (f), 12 (g), 16 (h), 20 (i), and 24 (j). The variation of the chemical shift of the signal marked with a red dot is shown in Figure 6c.

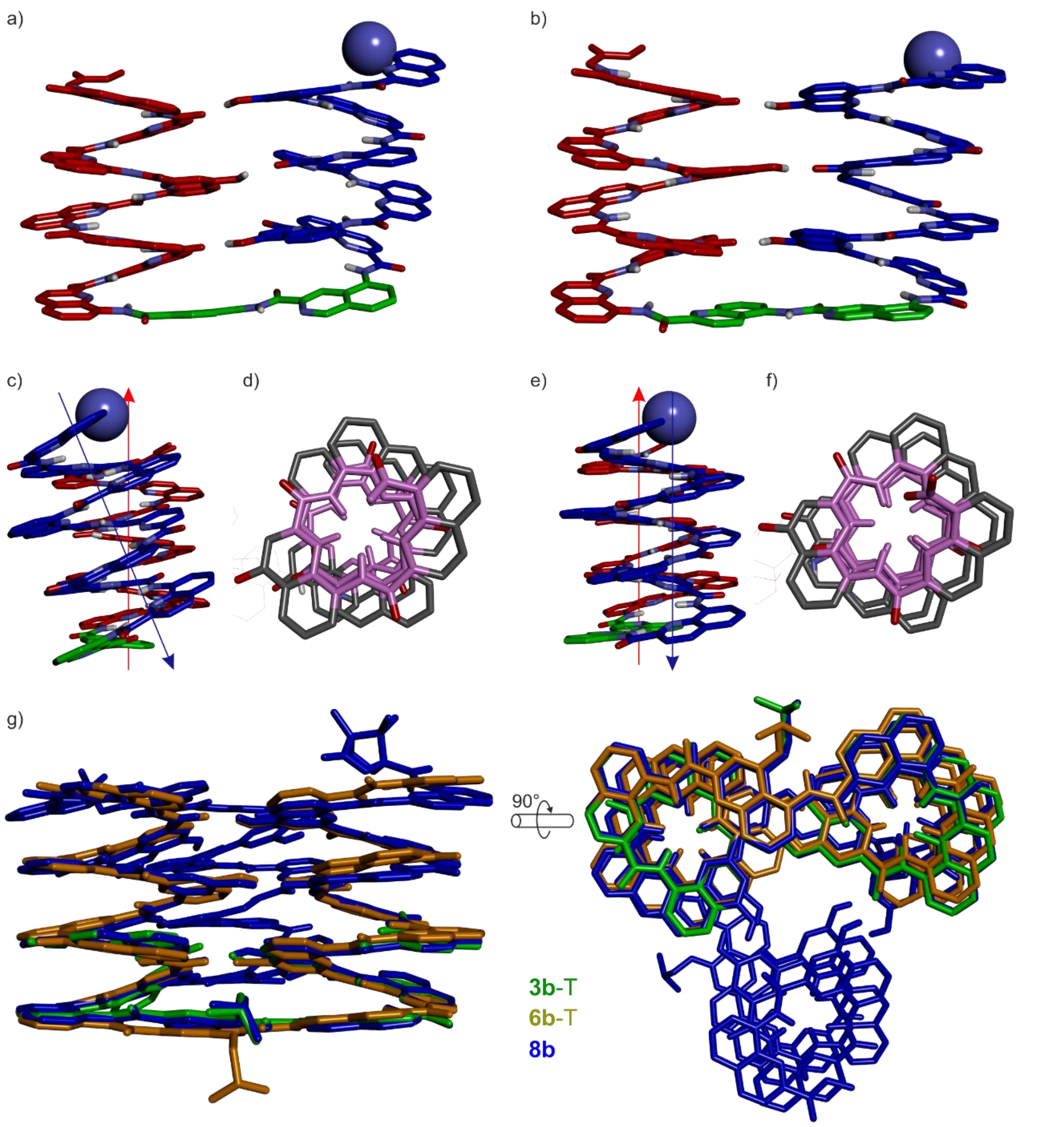

Figure S19. Molecular models of two conformations of 6b mediated different hydrogen bond patterns. Energy minimized model of conformer **6b**-S mediated by shifted a hydrogen bonding pattern (a) and of conformer **6b**-T mediated by a three helix bundle pattern (b). The main chain in the *P* helix, the *M* helix and in T6r is coloured in blue, red and green, respectively. The N-terminus is highlighted with a blue ball. The side views of **6b**-S (c) and **6b**-T (e) highlight the relative orientation of the helixes (axes shown as a blue arrow for the *P* helix and a red arrow for the *M* helix). The two helices are parallel in **6b**-T, not in **6b**-S. Views down the axis of the N-terminal helix of **6b**-S (d) and **6b**-T (f). The inner rim is highlighted in pink. It shows a 15-crown-5 shape in **6b**-T, not in **6b**-S, hinting at potential strain in the latter. g) Overlay of molecular models of **3b**-T (**3b** mediated by a three helix bundle pattern, by analogy with **6b**-T, in green), **6b**-T (in brown) and **8b** (in blue).

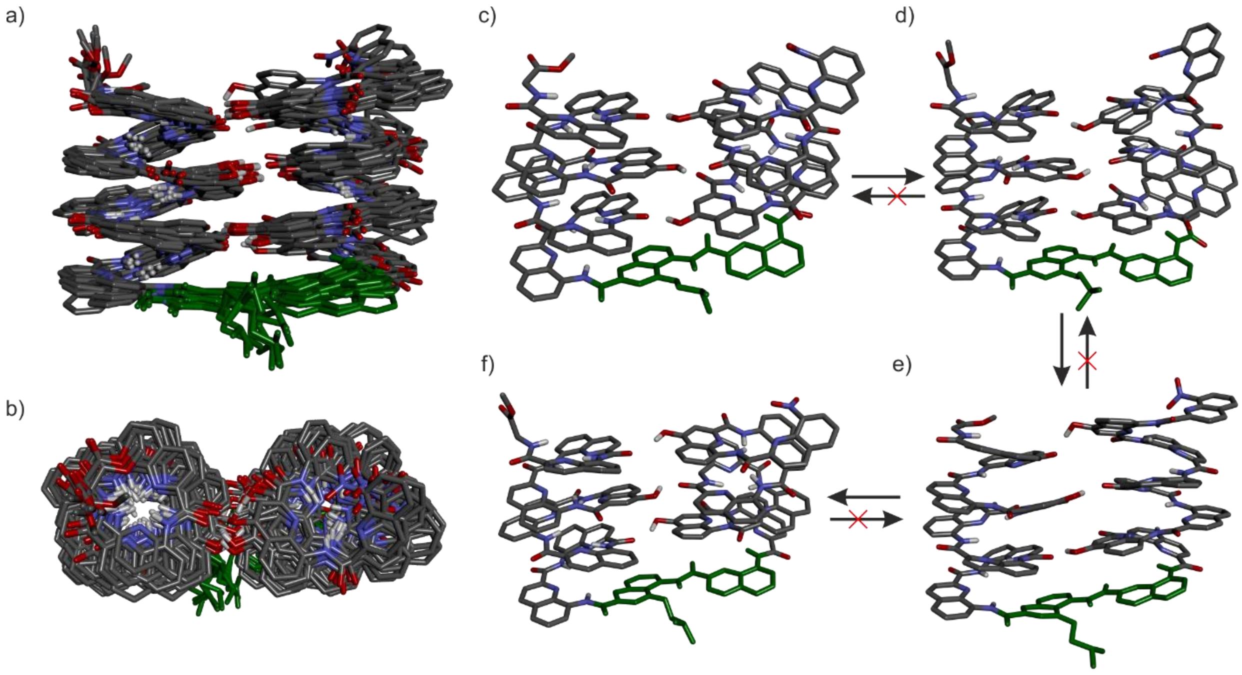

Figure S20. Molecular dynamic (MD) simulation of 6b. The MD simulation in Maestro (MMFFs, $CHCl_3$, TNCG, 300 K, 2 ns) was performed starting from the molecular model of **6b**-S shown in Figure S19. The side view (a) and top view (b) of the overlay of ten structures sampled during the MD simulation. c-f) Four structures extracted from the MD simulation. The structure gradually converted to structures that were similar to **6b**-T during the simulation.

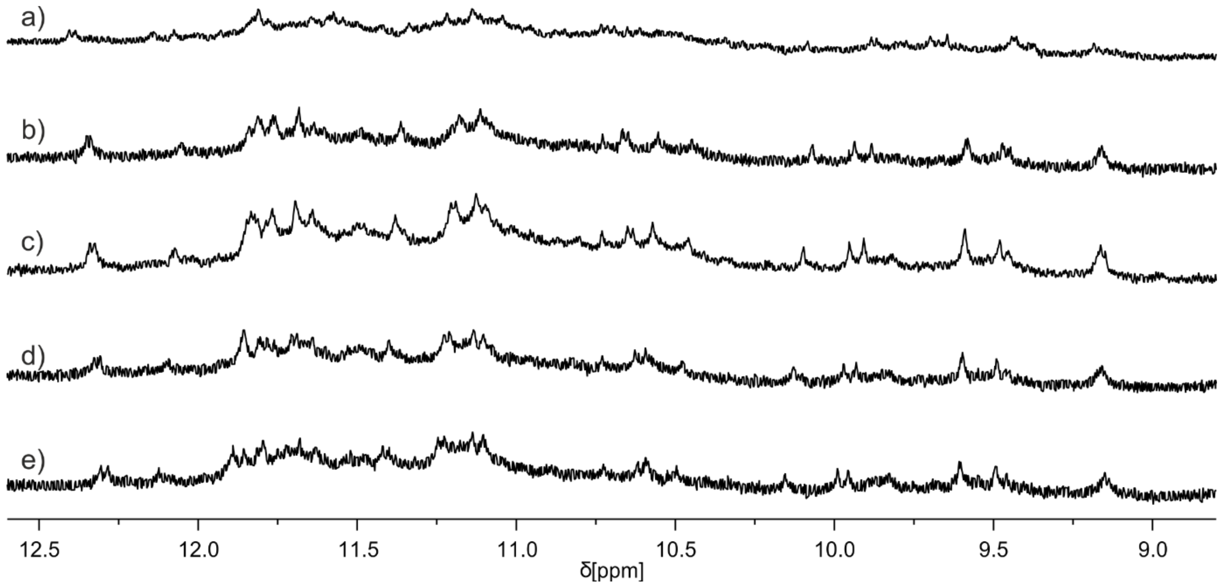

Figure S21. ^{1}H NMR spectra of 7b in $CDCl_3/CD_2Cl_2$ mixtures. Extracts of the ^{1}H NMR spectra (500 MHz) of **7b** in $CDCl_3/CD_2Cl_2$ mixtures showing the amide and hydroxy proton resonances. The vol% of CD_2Cl_2 are 100 (a), 75 (b), 50 (c), 25 (d), and 0 (e).

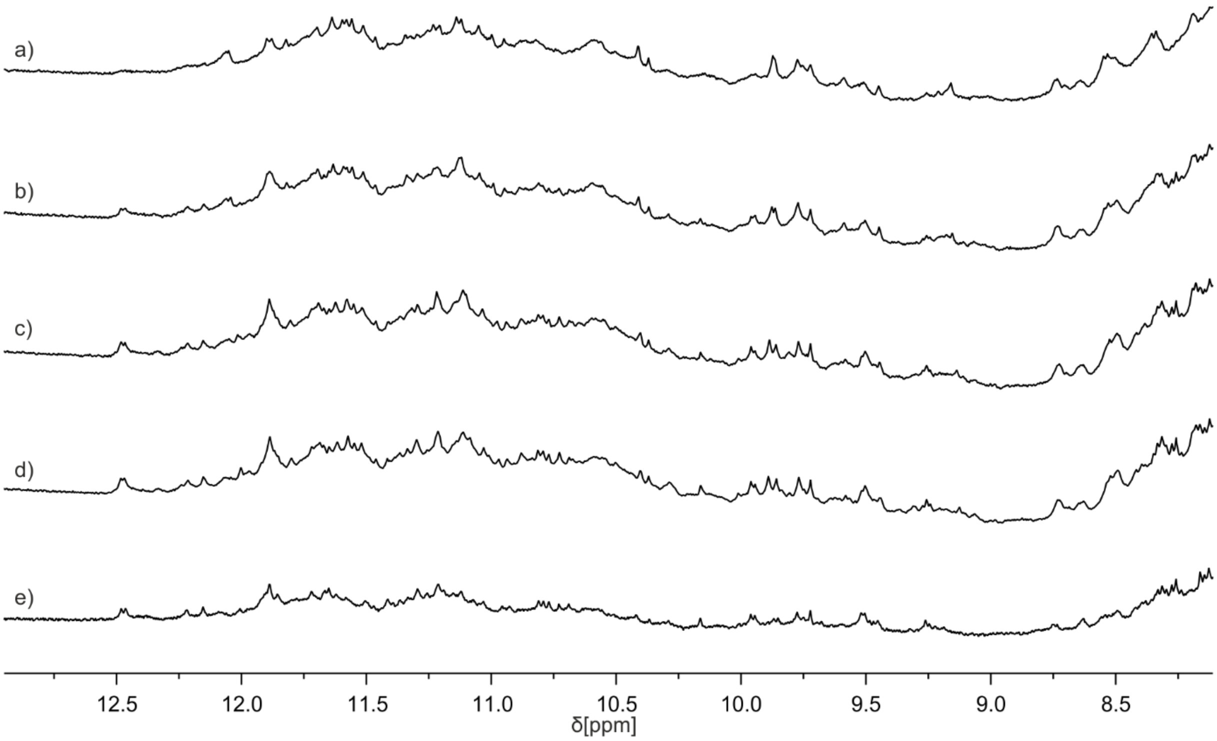

Figure S22. The spectrum of 7b changes marginally with time. Extracts of the ^{1}H NMR spectra (500 MHz, CD_2Cl_2) of **7b**. The equilibration time was 1 h (a), 1 day (b), 5 days (c), 1 week (d), and 2 weeks (e).

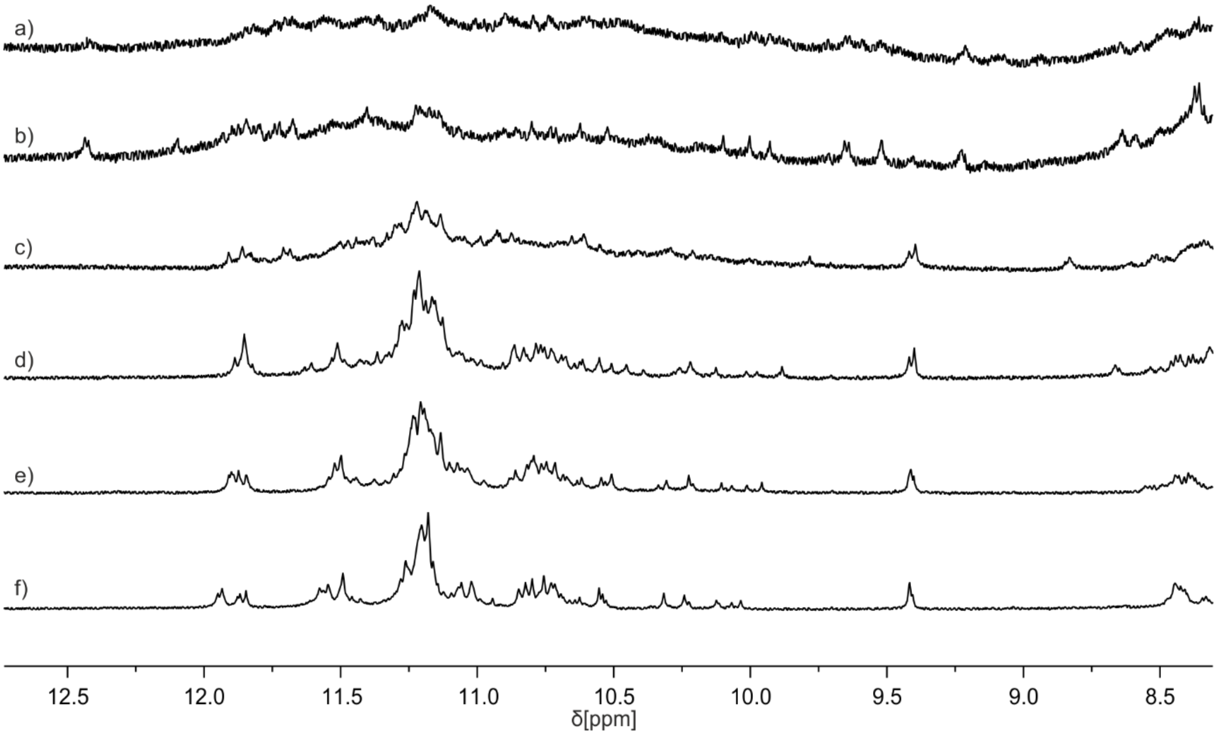

Figure S23. ^{1}H NMR observation of the DMSO-induced dissociation or disruption of 7b. Extracts of the ^{1}H NMR spectra (500 MHz) showing amide resonances of **7b** in CD_2Cl_2/DMSO-d_6 mixture. The vol% of DMSO-d_6 are 2 (a), 4 (b), 8 (c), 12 (d), 16 (e), and 20 (f).

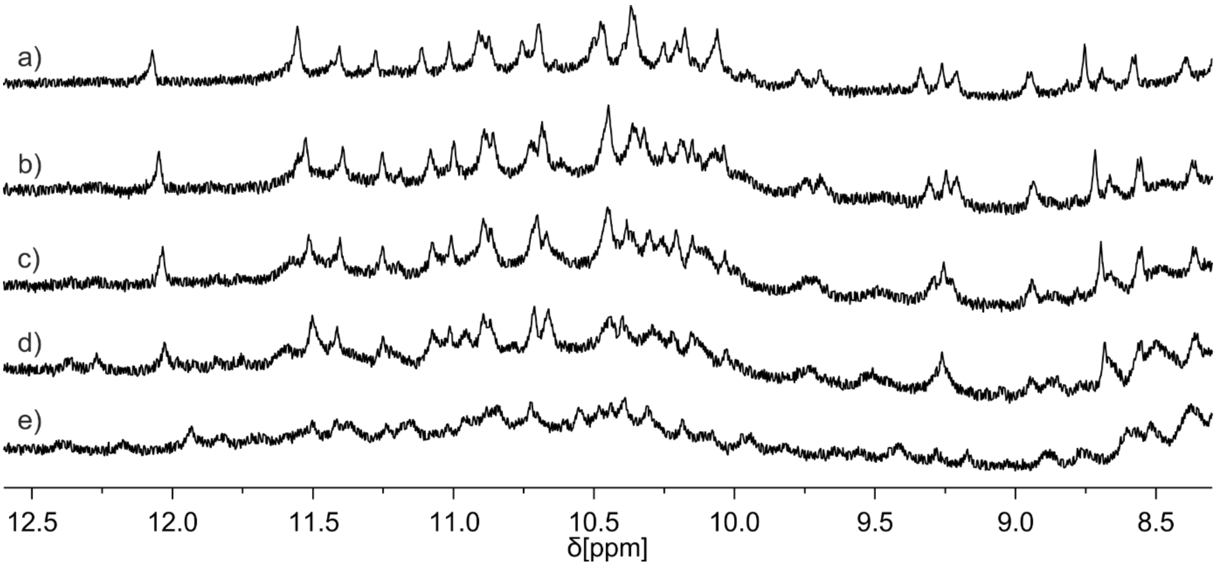

Figure S24. ^{1}H NMR spectra of 8b in $CDCl_3$/CD_2Cl_2 mixtures. Extracts of the ^{1}H NMR spectra (500 MHz) of **8b** in $CDCl_3$/CD_2Cl_2 mixtures showing the amide and hydroxy proton resonances. The vol% of CD_2Cl_2 are 100 (a), 75 (b), 50 (c), 25 (d), and 0 (e).

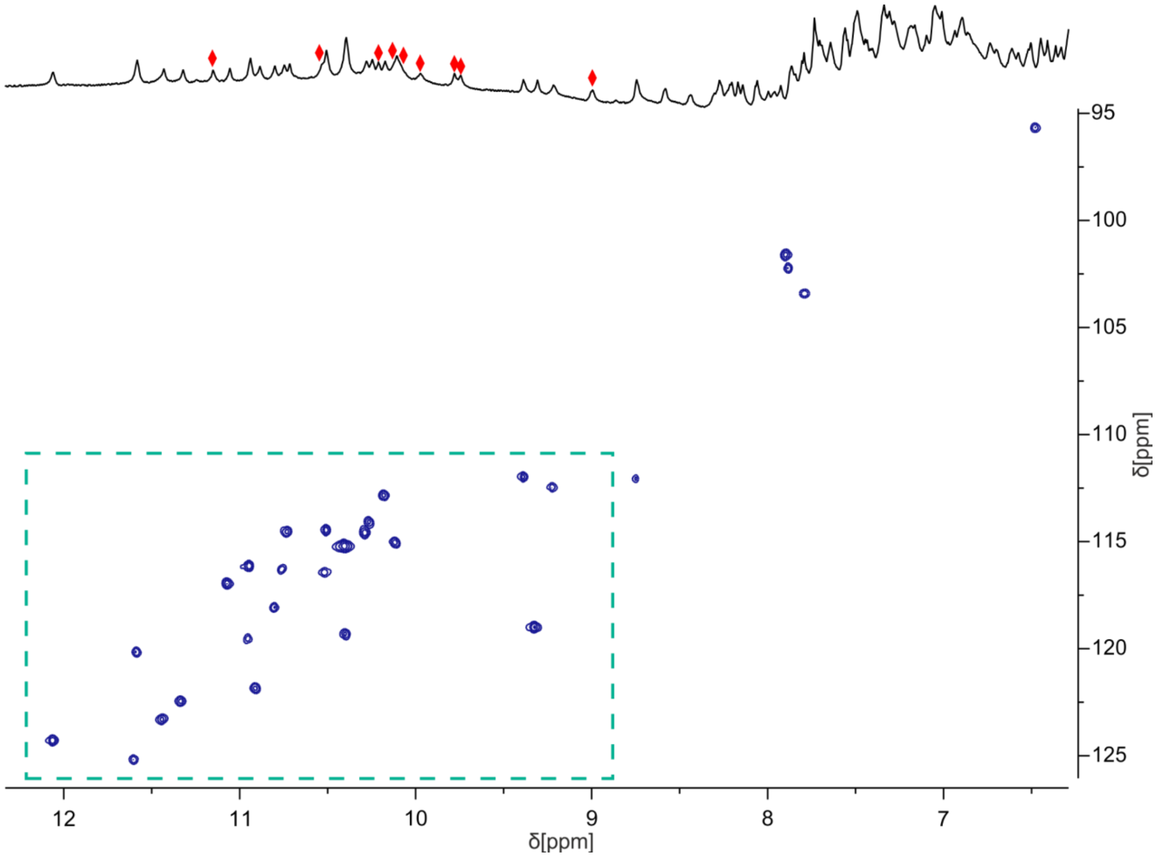

Figure S25. Indirect identification of the signals of hydrogen-bonded OH protons of 8b in $CDCl_3$. Extract of the ^{1}H,^{15}N HSQC NMR spectrum (500 MHz, CD_2Cl_2) of **8b**. Only NH resonances correlate, red diamonds indicate the signals of hydrogen-bonded OH protons. The region highlighted with green dashed box is shown in Figure 7d.

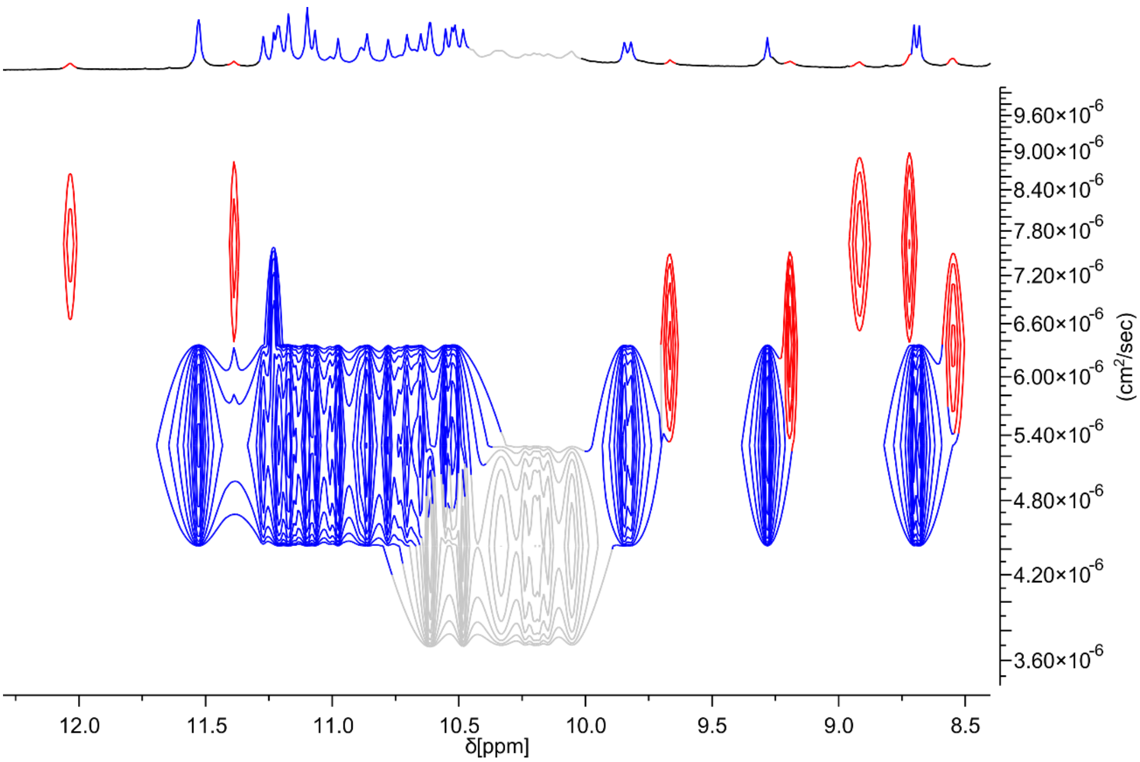

Figure S26. ^{1}H DOSY spectrum (500 MHz, CD_2Cl_2) of a mixture of 8a and 8b in a ratio of 1/0.5. The peaks corresponding to **8a** are highlighted in blue and those to **8b** are highlighted in red. Protected sequence **8a** is monomeric and its diffusion coefficient is substantially lower than that of **8b**, indicating that the latter is also monomeric and supporting a compact helix-turn-helix-turn-helix conformation of **8b**.

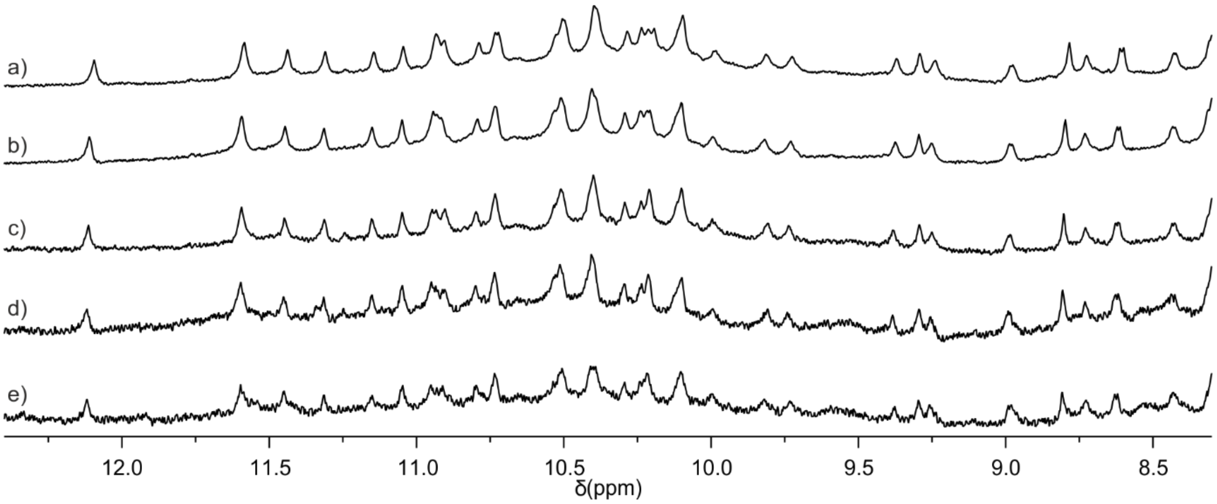

Figure S27. ^{1}H NMR spectra of 8b at different concentrations. Extracts of the ^{1}H NMR spectra of **8b** (500 MHz, 4% DMSO-d_6/CD_2Cl_2) at 1 mM (a), 0.5 mM (b), 0.25 mM (c), 0.1 mM (d), and 0.05 mM (e). No significant changes were observed.

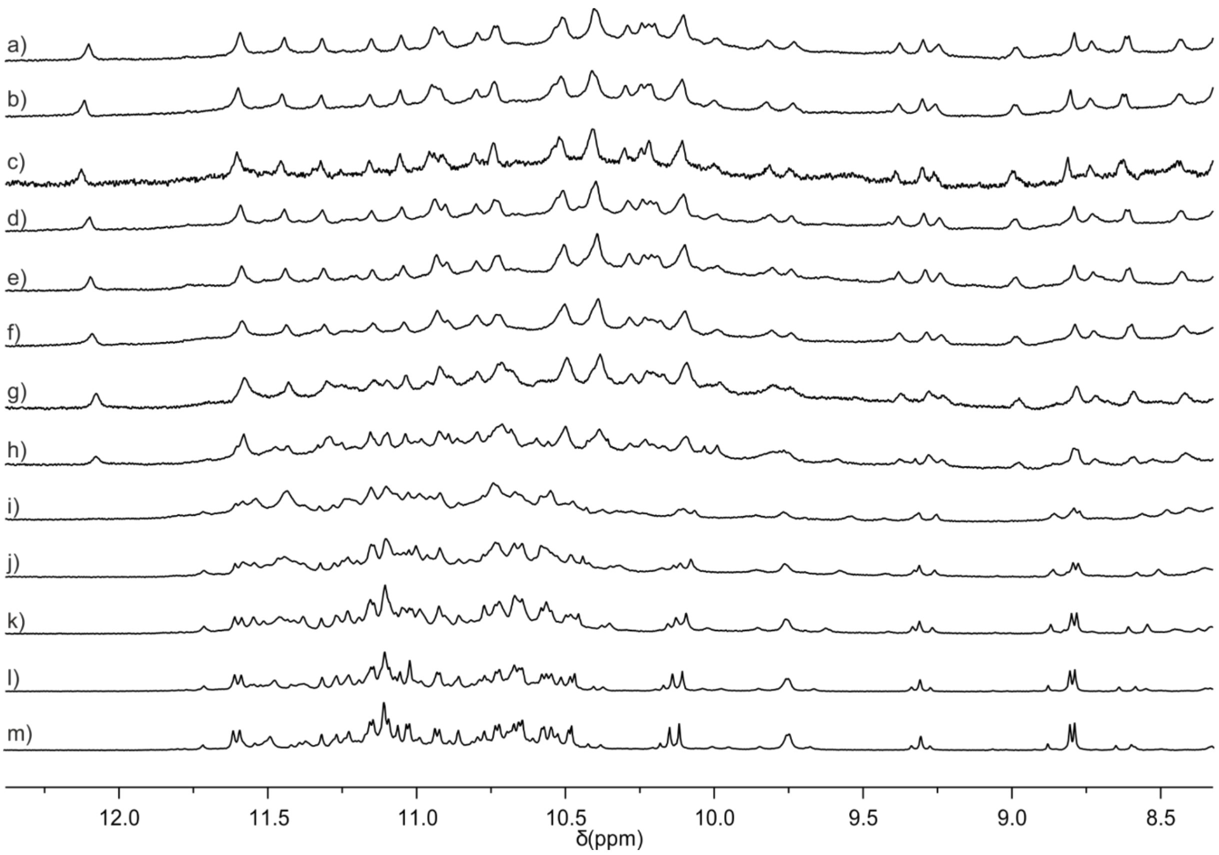

Figure S28. ^{1}H NMR observation of the DMSO-induced disruption of 8b. Extracts of the ^{1}H NMR spectra (500 MHz) showing amide resonances of **8b** in CD_2Cl_2/DMSO-d_6 mixture. The vol% of DMSO-d_6 are 1 (a), 2 (b), 4 (c), 6 (d), 8 (e), 10 (f), 12 (g), 14 (h), 16 (i), 18 (j), 20 (k), 22 (l), and 24 (m).

3 Supplementary methods

3.1 LC-MS analyses

LC-MS spectra were recorded on a Bruker microTOF II in positive ionization mode. The instrument was calibrated in positive mode by direct infusion of a calibration solution (Agilent Technologies ESI-L Low Concentration Tuning Mix). The HPLC line was an Ultimate 3000 RP-HPLC system (ThermoFisher Scientific) equipped with a Aeris^{TM} Widepore C4 column (2.1 x 150 mm, 3.6 µm) at a flow rate of 0.25 mL/min. 0.1 % formic acid and 0.025% TFA were added to the aqueous mobile phase (solvent A) and to acetonitrile (solvent B). The gradient is: 0-5 min, 70% to 100% solvent B; 5–14 min, 100% solvent B at 50 °C. The column eluent was monitored by UV detection at 214, 254, and 300 nm with a diode array detector. The sample was prepared by adding 10 µL of a solution of the sample in DCM (0.1 mg/mL) to 1 mL acetonitrile containing 0.05–0.1% formic acid.

3.2 Molecular modelling

Models were simulated by using Maestro version 11.5 (Schrödinger Inc.). Energy minimized structures were obtained using MacroModel energy minimization with the following parameters: force field: MMFFs; solvent: none; electrostatic treatment: constant dielectric; dielectric constant: 1.0; charges from: force field; cutoff: normal; Van der Waals: 7.0; electrostatic: 12.0; H-bond: 4.0; mini method: TNCG; maximum iterations: 2500; converge on gradient; convergence threshold: 0.05; constraints: distances. As a starting point, the crystal structure of **1** (CCDC entry # 1955168) was used. The Y unit at the N-terminus was replaced by a nitro group and the handedness and orientation of one helix was converted. The molecular model shown in Figure 3a, S4b,d was obtained after energy-minimization. After inserting two T6r linker units to the heterochiral trimer molecular model, the structure was energy-minimized to obtain the molecular model of **8b** shown in Figure 3d, 8a, S4e,f. As a starting point, the crystal structure of **3b** (CCDC entry # 2391429) was used and the C- and N-terminus were prolonged. The molecular model of **6b**-S shown in Figure S19a was obtained after energy-minimization. After removing the C-terminus helix and T6r unit of molecular model of **8b**, the molecular model of **6b**-T shown in Figure 6a, S19b was obtained after energy-minimization. The molecular model of **3b**-T shown in Figure S19g was obtained using a similar approach.

3.3 Molecular dynamic simulations

Molecular dynamic simulations were carried out using MacroModel version 11.1 (Maestro, Schrödinger Inc.). The crystal structure of **3b** (CCDC entry # 2391429) and the energy-minimized molecular model of **6b-S** and

6b-T were used as the object for the MD simulation. Stochastic dynamic simulations were obtained using MMFFs force field, $CHCl_3$ as the solvent, extended cutoff and TNCG method. The simulations were performed for 1 ns or 2 ns at 300 K, the time step of 1.5 fs and 1 ps as equilibration time. During the simulation, 10 structures were sampled and the sampled structures were minimized.

3.4 Nuclear magnetic resonance spectroscopy

NMR spectra were recorded on different NMR spectrometers: (1) an Avance III HD NMR spectrometer 400 MHz (Bruker BioSpin) for ^1H NMR and ^{13}C NMR spectra of some small molecules. (2) an Avance III HD NMR spectrometer 500 MHz (Bruker BioSpin) with CryoProbe™ Prodigy for ^1H NMR, ^{13}C NMR, $^1H,^{15}N$ HSQC, and DOSY spectra of some small molecules and foldamers. All NMR measurements were performed at 25 °C unless specified. Chemical shifts are described in part per million (ppm, δ) relative to the ^1H residual signal of the deuterated solvent used – meaning DMSO-d_6 (δ 2.50 ppm), pyridine-d_5 (δ 8.74 ppm), CD_2Cl_2 (δ 5.32 ppm) and $CDCl_3$ (δ 7.26 ppm). For the DMSO-d_6/$CDCl_3$ and DMSO-d_6/CD_2Cl_2 solvent mixtures, the chemical shifts were calibrated according to DMSO-d_6 (δ 2.50 ppm). For the CD_2Cl_2 and $CDCl_3$ solvent mixture, the chemical shifts were calibrated according to internal standard tetramethylsilane (δ 0.00 ppm).^1H NMR splitting patterns with observed first-order coupling are entitled as singlet (s), broad singlet (bs), doublet (d), triplet (t), doublet of doublets (dd) or multiplet (m). Coupling constants (J) are ported in Hz.

The ^1H NMR spectra of each sample were measured at different times respectively until no further change was observed within a week. We generally consider that at this point the compound reached equilibrium. When the sample reached equilibrium, re-dissolving the compound solid results in the equilibrated spectrum immediately without going through the equilibration process again. Complete disruption of the hydrogen bonds was achieved by dissolving the sample in polar solvents (such as DMSO, pyridine or MeOH/chloroform mixture), followed by removal of the solvent. When all of the hydrogen bonds were completely disrupted, it has to go through the equilibrium process again to reach the equilibrium. The equilibration time (the measurement time gap between two different conditions) of ^1H NMR spectra at different temperatures and in different proportions of DMSO-d_6/$CDCl_3$ and DMSO-d_6/CD_2Cl_2 solvents were usually several minutes. Due to similar properties of $CDCl_3$ and CD_2Cl_2, the individual samples in different proportions of $CDCl_3$/CD_2Cl_2 mixture were prepared and the ^1H NMR spectra of all of the samples were measured over time whereas no change was observed.

$^1H,^{15}N$ HSQC spectra were recorded with a phase-sensitive pulse sequence with sensitivity enhancement using trim pulses in inept transfer (hsqcetgpsi2) from the Bruker pulse program library. Data acquisition was

performed utilizing non-uniform sampling (NUS; NUS amount: 50% with an automatically created NUSList) yielding 1024 (F2) x 128 (F1) data points in Echo/Antiecho gradient selection mode. The recycling delay was 2.0 s and 64 transients per increment were applied at a sweep width of 2.5 kHz in F2 and 7 kHz in F1 resulting in an acquisition time of 0.1462 s. NUS processing was performed using the fully automated NUS processing tool provided by MestReNova. Zero filling in F1 has been used to yield a final matrix of 1K x 1K real points.

The DOSY spectrum was recorded by applying a pulse sequence with stimulated echo using stimulated echo for diffusion from the Bruker pulse program library (stegp1s). The diffusion delay Δ (big delta) and the diffusion gradient pulse length δ (little delta) were set as follows: 100 ms and 2.0 ms for the DOSY of **6a**/**6b** mixture, 150 ms and 2.0 ms for the DOSY of **8a**/**8b** mixture. The number of gradient steps was set to 32 with linear spacing starting from 2% reaching 95% of the full gradient strength in the final step. For each of the 32 gradient amplitudes, 256 transients of 65K complex data points were acquired. DOSY processing was performed with the DOSY processing tool from MestReNova (v.12.x64) employing the "Peak Heights Fit" algorithm including the "use existing peaks" and "autocorrect peak positions" with 128 points in diffusion dimension and a window of $1.00\cdot10^{-10}$ to $1.00\cdot10^{+00}$ cm^2 s^{-1}.

3.5 CD studies

The CD spectra of **4a** and **4b** were recorded on a Jasco J-1500 spectrometer with 1 mm quartz cuvette. The following parameters were used: wavelength range from 460 to 280 nm. Scan speed: 100 nm/min; accumulation: 2; response time: 1.0 s; bandwidth: 2; temperature: 20 °C; sensitivity: standard (100 mdeg); data pitch: 0.5 nm; nitrogen gas flow rate: 500 L/h. The CD spectra of **8a** and **8b** were recorded on a Jasco J-810 spectrometer with 1 mm quartz cuvette. The following parameters were used: wavelength range from 460 to 280 nm. Scan speed: 200 nm/min; accumulation: 3; response time: 1.0 s; bandwidth: 2; temperature: 20 °C; sensitivity: standard (100 mdeg); data pitch: 0.1 nm; nitrogen gas flow rate: 500 L/h. The sample solution of **4b** and **8b** in different proportions of DMSO/$CHCl_3$ or DMSO/CH_2Cl_2 solvents was prepared separately. The concentration is 0.1 mM for **4a**/**4b** and 0.05 mM for **8a**/**8b**. Δε values (in $cm^2\cdot mol^{-1}$) were obtained by using the formula: Δε = m°/(C.l.32980) where m = CD value in millidegrees; l = cuvette pathlength in cm; C = sample concentration in mol/L.

3.6 X-ray crystallography

Crystals of **2b** and **3b** were grown via liquid-liquid diffusion in NMR tubes from $CHCl_3$-MeCN and CH_2Cl_2-MeCN respectively. X-ray diffraction data was collected on a Rigaku XtaLAB Synergy R, HyPix-Arc 150 with Cu-

Kα radiation from a PhotonJet Rotating-anode X-ray Source at 100 K. Data processing was performed in CrysAlisPro (V. 1.171.43.130a).[4] The structures were solved by direct methods and preliminarily refined in the AutoChem-6[5] pipeline in CrysAlisPro. Further refinements were carried out with the SHELXL[6] package through Olex2 1.5-ac6-020.[7] All non-hydrogen atoms were refined anisotropically. Hydrogen atom positions were calculated geometrically and refined using the riding model. DFIX restraints were used to optimize model geometry and RIGU instructions were applied to some sidechain atom groups. Due to the high content of disordered chlorinated solvents in both structures, solvent masking was implied using the BYPASS implementation in Olex2. In structure **3b**, atoms beyond the ring adjacent oxygen (O00A) of the -O*i*Bu sidechain on the iso-Quinoline linker were deleted, since no complete chain could be modelled due to disorder. Final cif files were evaluated with the checkCIF algorithm through the IUCR online tool. Atomic coordinates and structure factors were deposited to the Cambridge Crystallographic Data Centre (CCDC) and can be accessed under the CCDC codes 2391393 (**2b**) and 2391429 (**3b**).

Table S1. Crystal data and refinement details

**Identification code**	**2b**	**3b**
**Chemical formula**	$C_{99}H_{92}N_{16}O_{18} \cdot CHCl_3$ solvent	$C_{102}H_{92}N_{16}O_{18}$ · (CH_2Cl_2 3 MeCN) solvent
**Formula weight**	1793.92	1829.95
**Crystal growth conditions**	liquid-liquid diffusion<br>$CHCl_3$-MeCN	liquid-liquid diffusion<br>CH_2Cl_2-MeCN
**Crystal size (mm^3)**	0.6 × 0.15 × 0.1	0.42 × 0.37 × 0.21
**Crystal system**	Triclinic	
**Spacegroup**	*P*-1	
**Unit cell dimensions (Å, °)**	a = 14.3247(2)	a = 13.16060(10)
	b = 17.1233(2)	b = 17.3713(2)
	c = 23.0646(2)	c = 24.3400(2)
	α = 96.8220(10)	α = 95.6610(10)
	β = 98.8460(10)	β = 93.9890(10)

	γ = 108.1960(10)	γ = 105.3540(10)
**Volume (Å^3)**	5226.07(11)	5307.66(7)
**Z**	2	2
**ρ(calc) (g/cm^3)**	1.215	1.234
**Absorption coefficient (mm^{-1})**	1.378	1.155
**F (000)**	1998.0	2063.0
**Radiation**	Cu Kα (λ = 1.54184)	
**T (K) collection**	100.0	
**2Θ range for data collection/°**	6.188 to 151.082	6.82 to 151.304
**Reflections collected**	106475	113760
**Independent reflections**	20937 [R_{int} = 0.0359, R_{sigma} = 0.0235]	21386 [R_{int} = 0.0243, R_{sigma} = 0.0189]
**Data/restraints/parameters**	20937/4/1305	21386/2/1335
**Goodness-of-fit on F^2**	1.052	1.073
**Final R indexes [I>=2σ (I)]**	R_1 = 0.0761, wR_2 = 0.2384	R_1 = 0.0806, wR_2 = 0.2312
**Final R indexes [all data]**	R_1 = 0.0854, wR_2 = 0.2489	R_1 = 0.0864, wR_2 = 0.2372
**Largest diff. peak/hole**	0.87/-1.28	2.92/-1.53
**CCDC accession #**	2391393	2391429

4 Synthetic Schemes

4.1 Synthesis of turn units

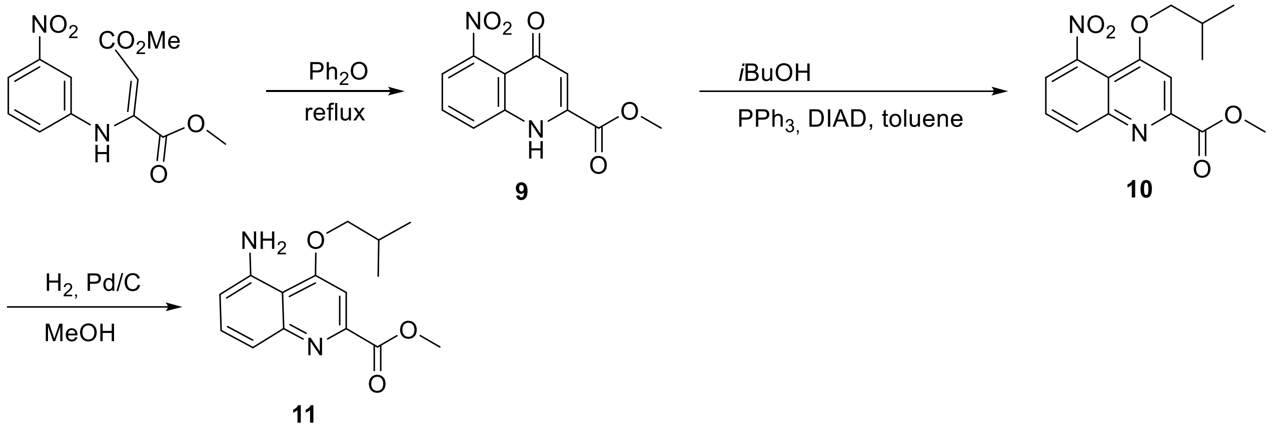

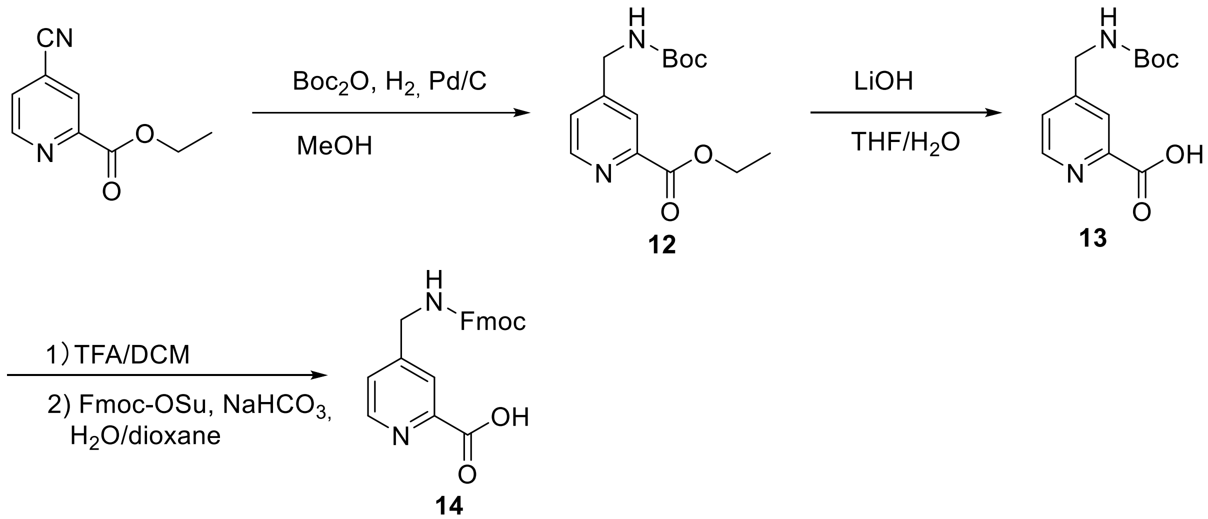

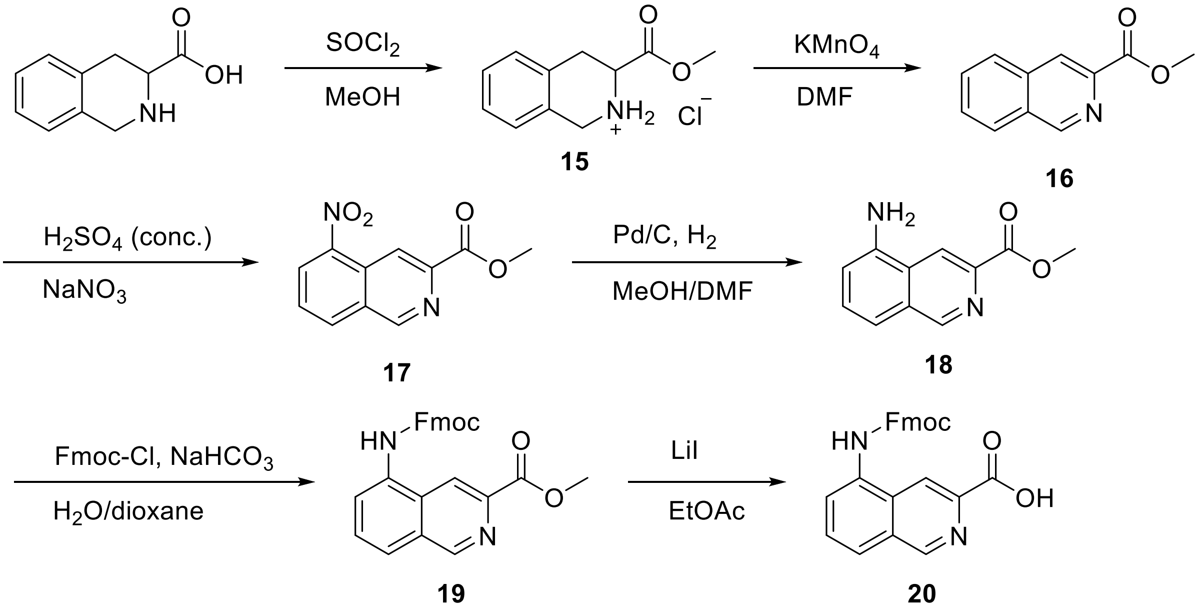

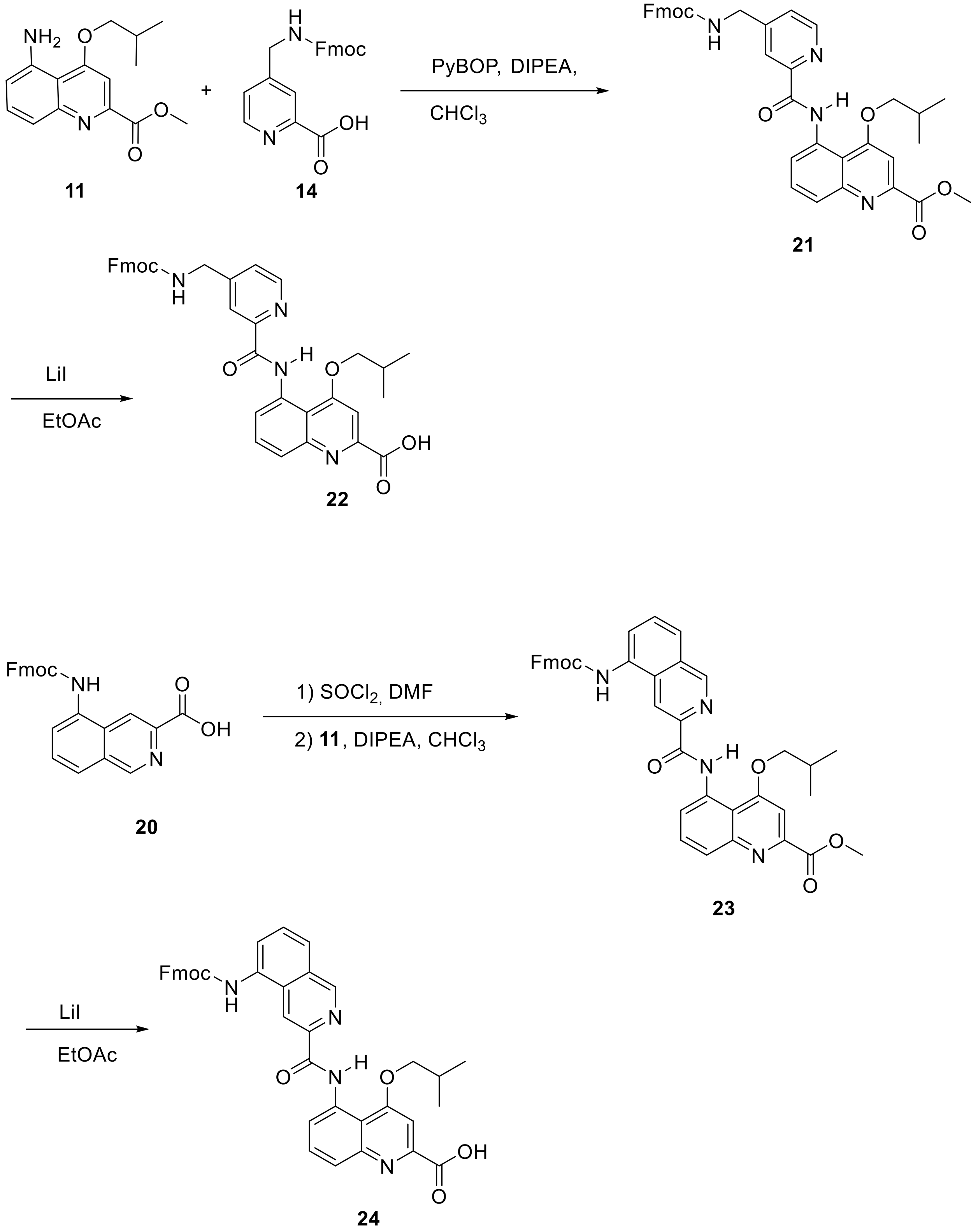

Scheme 1. Synthesis of linker units Fmoc-T6f-OH (**22**) and Fmoc-T6r-OH (**24**).

4.2 synthesis of foldamers

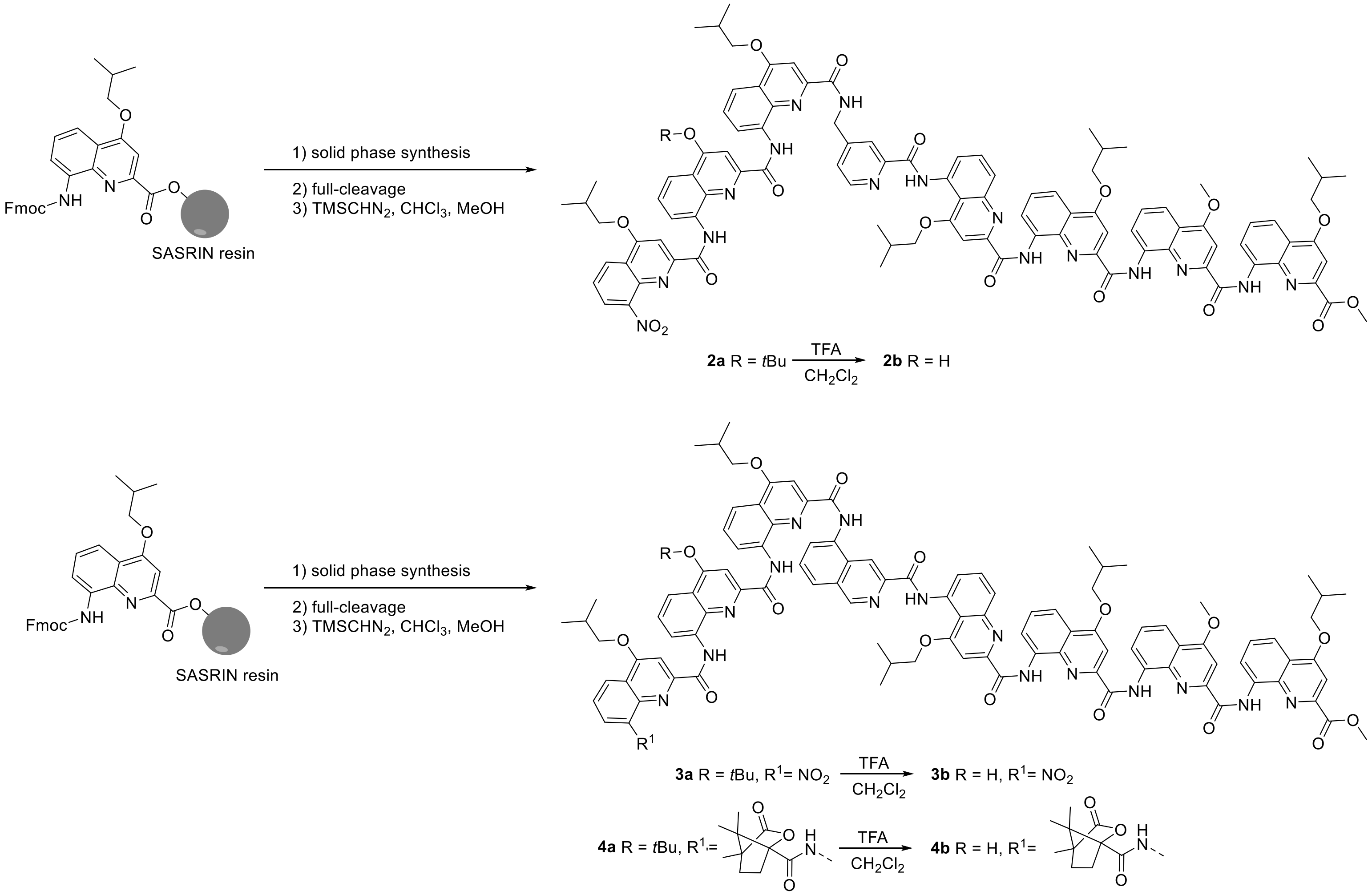

Scheme 2. Synthesis of **2a**, **2b**, **3a**, **3b**, **4a,** and **4b**.

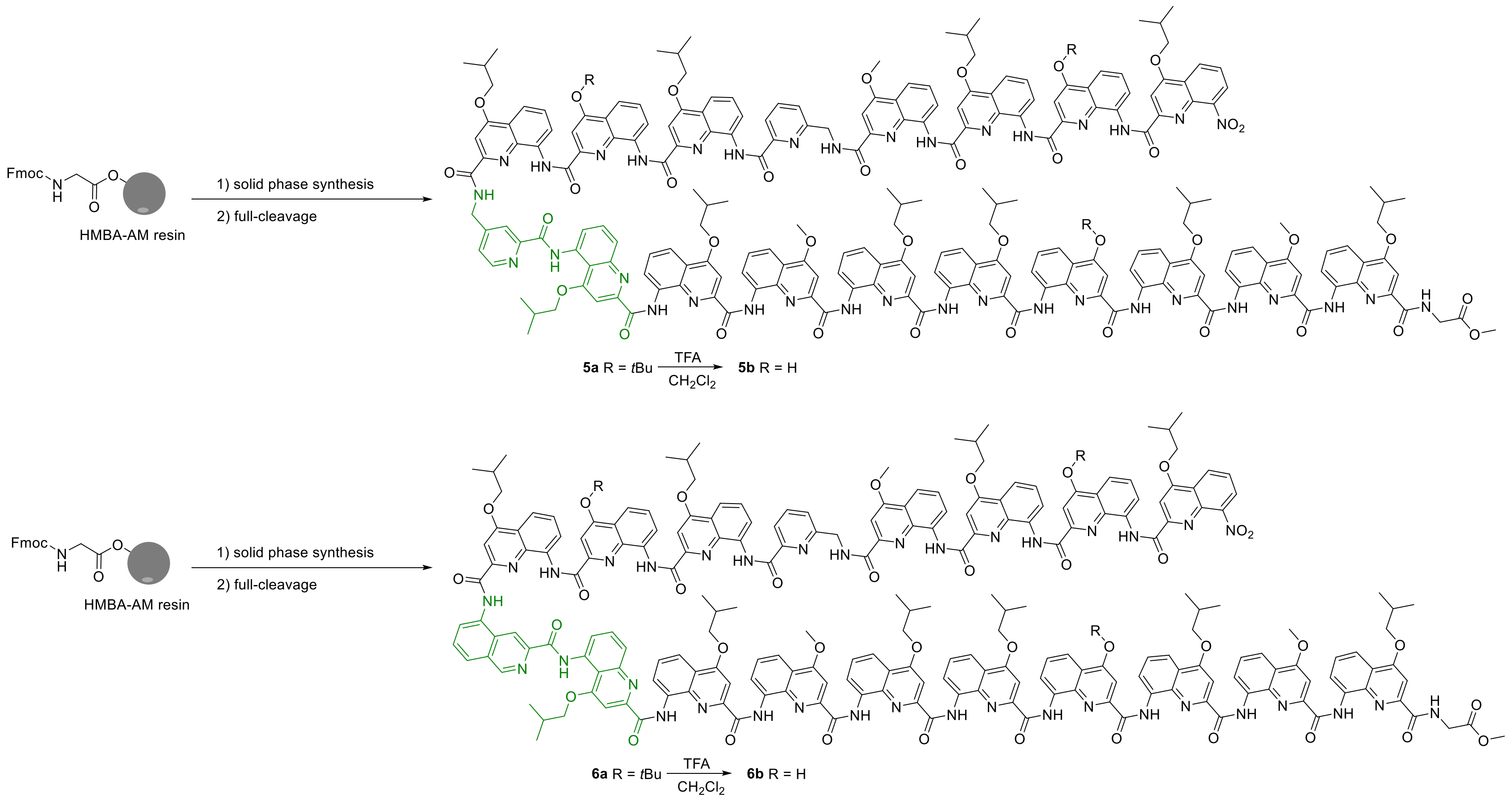

Scheme 3. Synthesis of **5a**, **5b**, **6a**, and **6b**.

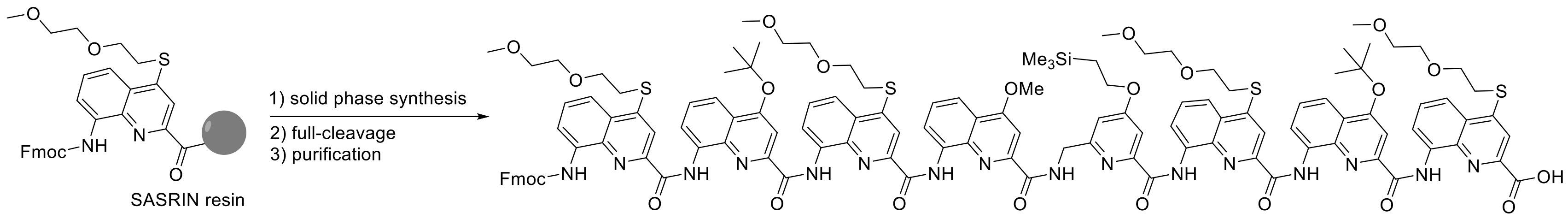

25

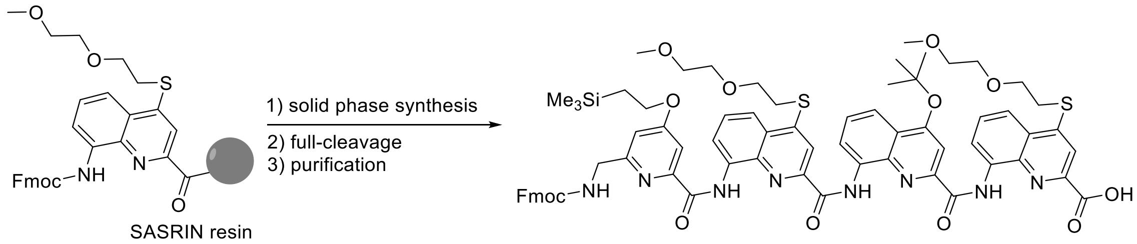
1) solid phase synthesis
2) full-cleavage
3) purification
SASRIN resin

26

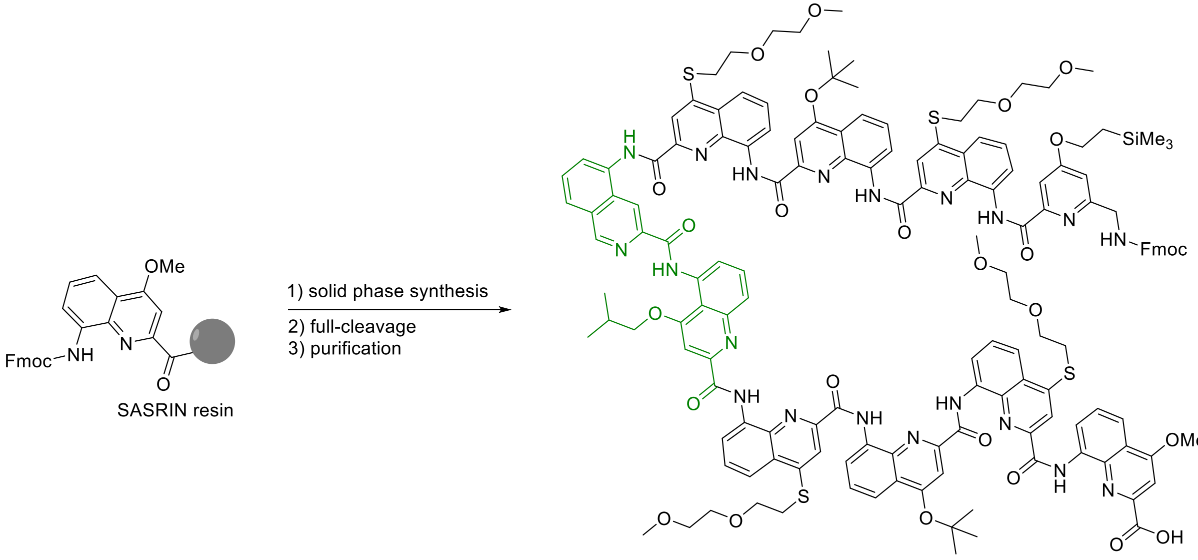
1) solid phase synthesis
2) full-cleavage
3) purification
SASRIN resin

27

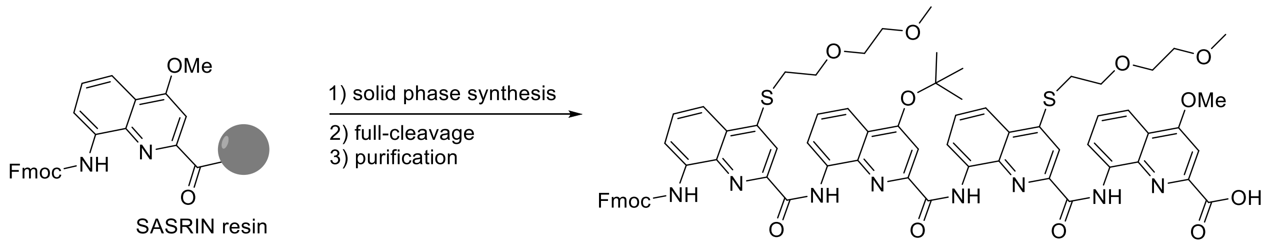
1) solid phase synthesis
2) full-cleavage
3) purification
SASRIN resin

28

Scheme 4. Synthesis of the fragments.

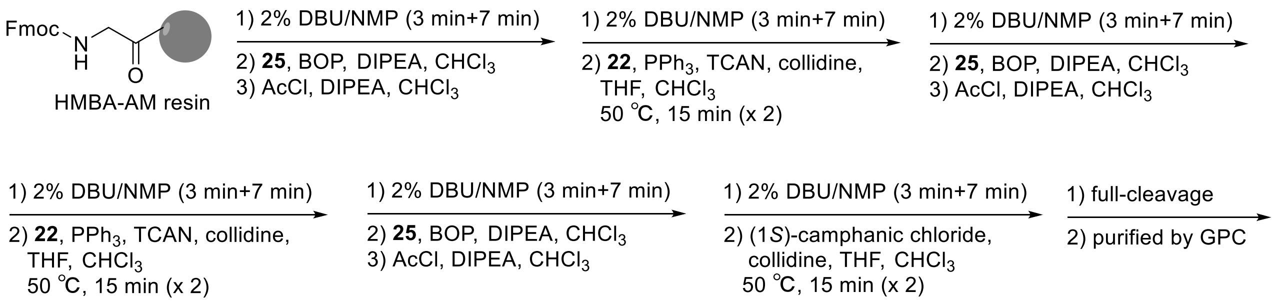

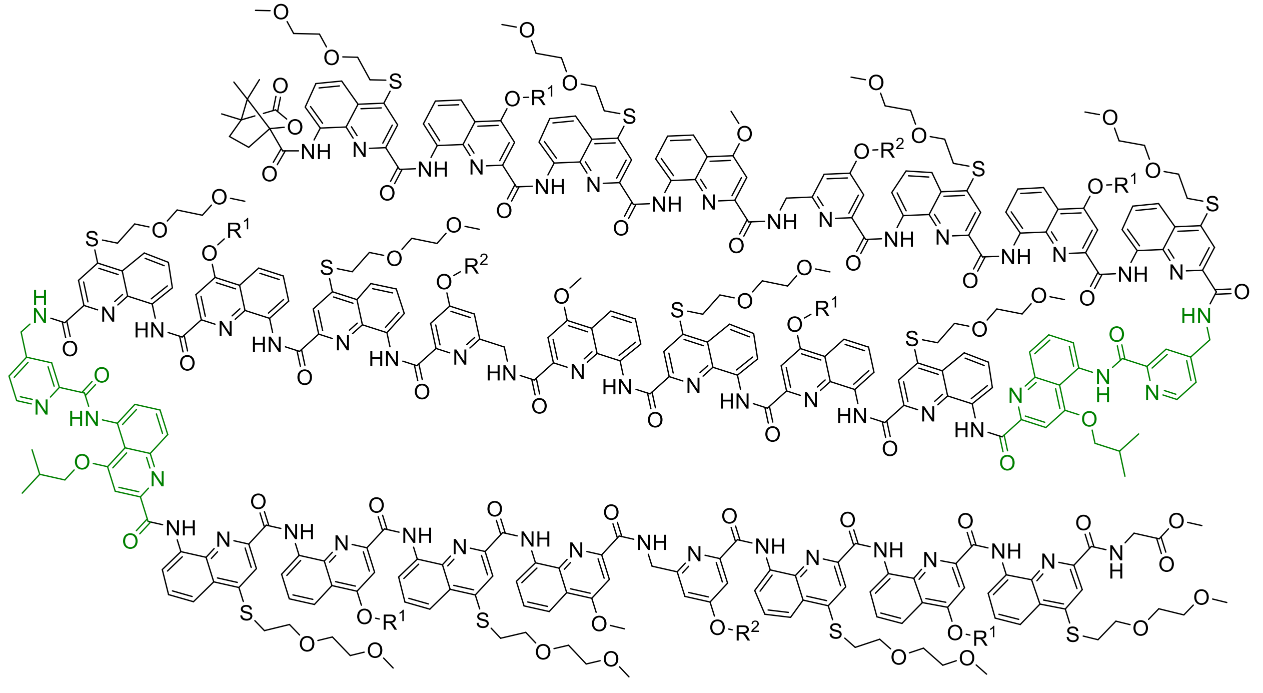

7a: R^1 = *t*Bu, R^2 = TMSE $\xrightarrow{\text{1/1 TFA/DCM}}$ **7b**: R^1 = H, R^2 = H

Scheme 5. Synthesis of **7a** and **7b** by fragment condensation strategy.

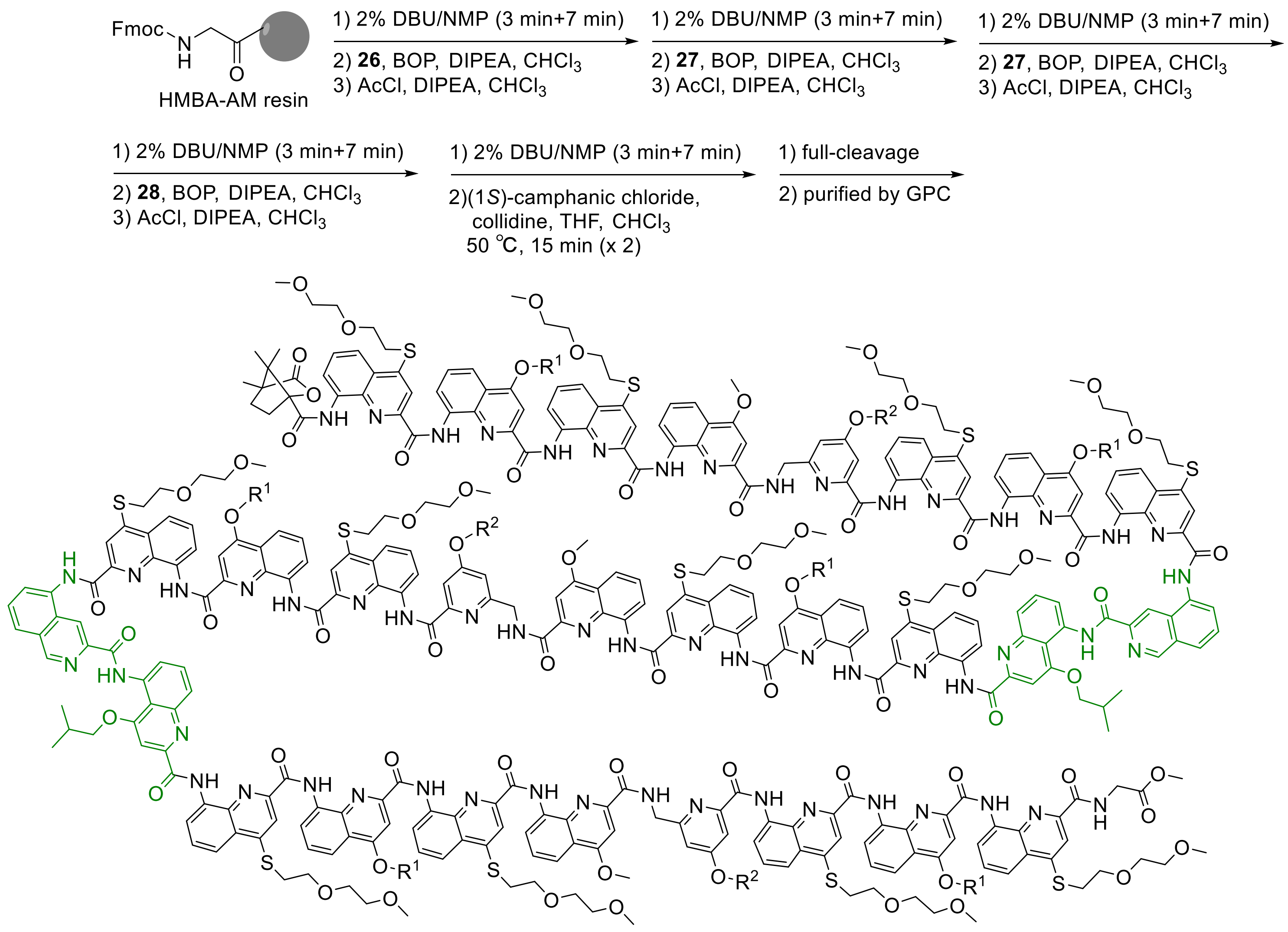

8a: R_1 = *t*Bu, R_2 = TMSE —(1/1 TFA/DCM)→ **8b**: R_1 = H, R_2 = H

Scheme 6. Synthesis of **8a** and **8b** by fragment condensation strategy.

5 Synthetic Procedures

5.1 General methods

Commercially available reagents were purchased from Sigma-Aldrich, Alfa-Aesar or TCI and were used without further purification unless specified. HMBA-AM resin (200–400 mesh, loading 0.8–1.2 mmol/g) was purchased from Iris-biotech. SASRIN resin (200–400 mesh, 0.80-1.20 mmol/g) was purchased from Bachem. THF, DCM and toluene were dried over alumina columns (MBRAUN SPS-800 solvent purification system). *N*,*N*-diisopropylethylamine and chloroform were distilled over CaH_2 prior to use. Extra dry DMF was purchased from Sigma-Aldrich. Ultrapure water was obtained via a Stakpure OmniaPure-T UV-TOC ultrapure water system. Reactions were monitored by thin layer chromatography (TLC) on Merck silica gel 60-F254 plates and observed under UV light. Column chromatography purifications were carried out on Merck GEDURAN Si60 (40–63 µm).

Analytical reversed-phase (RP) high-performance liquid chromatography (HPLC) was performed on a Thermo Fisher Scientific Ultimate 3000 HPLC System using Macherey-Nagel Nucleodur C8 Gravity columns (4 × 50 mm, 5 µm). UV absorbance was monitored at 300 nm and 254 nm, if not stated otherwise. The semi-preparative HPLC was performed on a Waters system equipped with a 2545 Quaternary Gradient Module with an automated fraction collector system on a XBridge® Prep C8 OBD™ column (19 × 150 mm, 5 µm) at a flow rate of 25 mL/min. 0.1 % TFA was added to the aqueous mobile phase (referred to as mobile phase A) and to acetonitrile (referred to as mobile phase B). The gradient is: 0–5 min, 90% to 100% solvent B; 5–25 min, 100% solvent B at r.t.. The column eluent was monitored by UV detection at 254 and 300 nm with a diode array detector. Preparative recycling gel permeation chromatography (GPC) was carried out on JAIGEL 20*600 mm columns (Japan Analytical Industry) in chloroform containing 1% ethanol and 0.25% trimethylamine as mobile phase, with a flow rate of 10 mL/min. Monitoring by UV detection was carried out at 214 nm, 254 nm, 300 nm and 400 nm.

The ultraviolet-visible (UV/Vis) absorbance measurements were done with a Thermo Fisher Scientific Nanodrop One instrument using a 1 cm path-length quartz cuvette. Circular dichroism (CD) spectra were measured on Jasco J-810 or Jasco J-1500 spectrometers. Measurements were performed at 20 °C if not stated otherwise.

Solid phase synthesis (SPS) was performed manually under MW irradiation on a CEM Discover (Liberty Bio) microwave oven using a reaction vessel and an internal fiber optic probe for temperature control, or with a fully automated synthesizer followed by previously reported protocol.[8]

5.2 synthesis of monomers and the turn units.

The Fmoc-Y̲-OH,[2] Fmoc-Q^M-OH,[9] Fmoc-Q^D-OH,[3] Fmoc-Q^B-OH,[10] Fmoc-X̲-OH[3] and Fmoc-P-OH[11] were synthesized according to literature. All of the Fmoc-protected monomers were≥ 98% pure before being used in the solid phase synthesis.

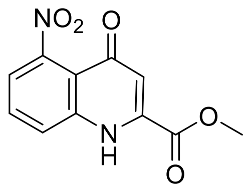

Compound 9. Diphenyl ether (200 mL) was heated to its boiling point, then dimethyl 2-((3-nitrophenyl)amino)maleate[12] (10 g, 35.7 mmol) was added. The reaction mixture was maintained at boiling for 20 min, after which it was allowed to cool to room temperature. Cyclohexane (200 mL) was added and the product was filtered off. The product was washed thoroughly with cyclohexane (100 mL × 3). DMF (100 mL) was added to the residue and the side product methyl 4-isobutoxy-7-nitroquinoline-2-carboxylate was precipitated and removed by filtration. The DMF solution was collected and concentrated in vacuo, yielding the desired compound as a brownish solid (3.62 g, 41% yield). **^{1}H NMR** (400 MHz, DMSO-*d*$_6$) δ 12.53 (s, 1H), 8.14 (d, *J* = 8.6, 1H), 7.83 (dd, *J* = 8.6, 7.5 Hz, 1H), 7.59 (d, *J* = 7.5 Hz, 1H), 6.65 (s, 1H), 3.97 (s, 3H). **^{13}C NMR** (101 MHz, DMSO-*d*$_6$) δ 174.6, 162.1, 147.8, 140.9, 138.3, 132.7, 122.4, 118.1, 115.9, 111.5, 53.7. **HRMS** (ESI+) calcd. for $C_{11}H_8N_2O_5$ [M+H]$^+$ 249.0506, found 249.0505.

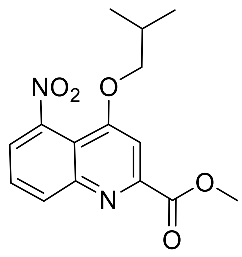

Compound 10. Compound **9** (1 g, 4.0 mmol, 1 equiv.) and PPh_3 (1.59 g, 6.0 mmol, 1.5 equiv.) were dissolved in dry toluene (15 mL) under N_2. Diisopropyl azodicarboxylate (1.23 mL, 6.0 mmol, 1.5 equiv.) was added dropwise to the solution. The mixture was stirred at 70 °C for 1 h and the formation of a white precipitate was observed. As isobutanol (0.6 mL, 6.0 mmol, 1.5 equiv.) was added the precipitation disappeared and the reaction mixture was stirred overnight at 70 °C. The solvent was removed and the crude product was purified by silica gel chromatography (EtOAc/cyclohexane 2:3). The product was crystallized from acetonitrile and obtained as a light-yellow solid (833 mg, 68% yield). **^{1}H NMR** (400 MHz, $CDCl_3$) δ 8.37 (dd, *J* = 8.6, 1.0 Hz, 1H), 7.77 (dd, *J* = 8.6, 7.5 Hz, 1H), 7.66 (s, 1H), 7.64 (dd, *J* = 7.5, 1.0 Hz, 1H), 4.09 (s, 3H), 4.02 (d, *J* = 6.2 Hz, 2H), 2.19 (nonet, *J* = 6.6 Hz, 1H), 1.07 (d, *J* = 6.8 Hz, 6H). **^{13}C NMR** (101 MHz, $CDCl_3$) δ 165.6, 161.5, 150.6, 149.1, 146.5, 133.5, 129.2, 122.1, 113.7, 103.1, 76.7, 53.7, 28.2, 19.3. **HRMS** (ESI+) calcd. for $C_{15}H_{16}N_2O_5$ [M+H]$^+$ 305.1132, found 305.1132.

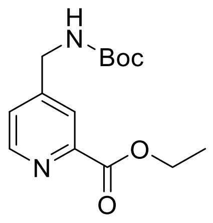

Compound 12. Ethyl 4-cyano-2-pyridinecarboxylate (300 mg, 1.7 mmol) and Boc_2O (930 mg, 4.3 mmol) were dissolved in dry MeOH (4 mL) under N_2 atmosphere. Then the Pd/C (30 mg) was added and the N_2 was replaced by H_2. The reaction mixture was stirred at r.t. overnight under H_2 atmosphere. The solution was then filtered and concentrated. The crude product was purified by silica gel chromatography (cyclohexane/EtOAc 2:3). The product was obtained as a white solid (320 mg, 67%). **^{1}H NMR** (500 MHz, $CDCl_3$) δ 8.70 (d, *J* = 4.9 Hz, 1H), 8.06–8.02 (m, 1H), 7.42–7.37 (m, 1H), 5.03 (s, 1H), 4.48 (q, *J* = 7.2 Hz, 2H), 4.43–4.38 (m, 2H), 1.47 (s, 9H), 1.45 (t, *J* = 7.2 Hz, 3H). **^{13}C NMR** (126 MHz, $CDCl_3$) δ 165.2, 155.9, 150.0 149.9, 148.5, 125.1, 123.3, 80.3, 62.1, 43.4, 28.4, 14.4. **HRMS** (ESI+) calcd. for $C_{14}H_{20}N_2O_4$ [M+H]$^+$ 281.1496, found 281.1467.

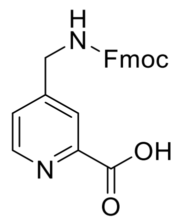

Compound 14. Compound **12** (320 mg, 1.1 mmol) was dissolved in THF/H_2O 5:1 (30 mL), then LiOH (55 mg, 2.2 mmol) was added. The reaction mixture was stirred at r.t. for 2 h, with completion indicated by TLC. The reaction mixture was diluted with water. The pH was adjusted to 4 by adding citric acid solution (5%, w/w). The product was extracted with DCM (3 x 50 mL). The organic layers were combined and dried over $MgSO_4$, filtered and concentrated. The product **13** was obtained as a solid in almost quantitative yield and used directly in the next step without further purification. Compound **13** (7.5 g, 29.7 mmol) was dissolved in DCM (37 mL). TFA (12 mL) was slowly added to the solution and stirred at r.t. until TLC indicated complete Boc deprotection. The solvent was removed under reduced pressure to provide the amino acid as a TFA salt. Then the amino acid was dissolved in a mixture of 1,4-dioxane (300 mL) and saturated $NaHCO_3$ solution (200 mL). The solution was cooled to 0 °C and a solution of Fmoc-OSu (11.0 g, 32.6 mmol, in 57 mL 1,4-dioxane) was added dropwise over 1 h. The reaction mixture was stirred at r.t. overnight. The resulting mixture was diluted with water and the pH was adjusted to 3 by dropwise addition of citric acid solution (5%, w/w). The precipitate was collected by filtration and washed with water. The product was obtained as a white solid. (6.8 g, 61%). **^{1}H NMR** (500 MHz, DMSO-*d*$_6$) δ 8.63 (d, *J* = 5.0 Hz, 1H), 8.03 (t, *J* = 6.1 Hz, 1H), 7.97 (s, 1H), 7.89 (d, *J* = 7.5 Hz, 2H), 7.70 (d, *J* = 7.5 Hz, 2H), 7.46–7.39 (m, 3H), 7.33 (t, *J* = 7.5, 2H), 4.38 (d, *J* = 6.9 Hz, 2H), 4.30 (d, *J* = 6.3 Hz, 2H), 4.25 (t, *J* = 6.9 Hz, 1H). **^{13}C NMR** (126 MHz, DMSO-*d*$_6$) δ 166.2, 156.5, 150.4, 149.4, 148.5, 143.8, 140.8, 127.6, 127.1, 125.1, 125.0, 124.7, 122.7, 120.2, 65.5, 46.8, 42.7. **HRMS** (ESI+) calcd. for $C_{22}H_{18}N_2O_4$ $[M+H]^+$ 375.1339, found 375.1338.

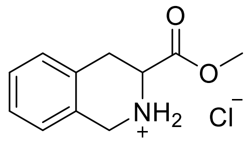

Compound 15. (*RS*)-1,2,3,4-tetrahydroisoquinoline-3-carboxylic acid (5 g, 28.2 mmol) was dissolved in MeOH (200 mL) and the reaction solution was cooled to 0 °C. Thionyl chloride (10 mL, 51.3 mmol) was added dropwise. The reaction was stirred at r.t. overnight. The solvent was evaporated and excess thionyl chloride was removed in vacuo. The residue was washed with diethyl ether to afford the product as a white solid (5.4 g, 99%). **^{1}H NMR** (500 MHz, DMSO-*d*$_6$) δ 10.22 (s, 2H), 7.26 (s, 4H), 4.57 (dd, *J* = 11.1, 5.1 Hz, 1H), 4.37–4.28 (m, 2H), 3.81 (s, 3H), 3.30 (dd, *J* = 16.9, 5.1 Hz, 1H), 3.17 (dd, *J* = 16.9, 11.1 Hz, 1H). **^{13}C NMR** (126 MHz, DMSO-*d*$_6$) δ 168.9, 130.5, 128.8, 128.4, 127.5, 126.9, 126.6, 53.1, 53.0, 43.8, 28.0. **HRMS** (ESI+) calcd. for $C_{11}H_{13}NO_2$ $[M+H]^+$ 192.1019, found 192.1018.

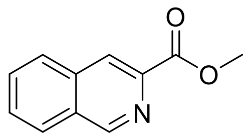

Compound 16. Compound **15** (12.7 g, 66.5 mmol) was dissolved in DMF (250 mL) and the solution was cooled to 0 °C. KMnO4 (7.35 g, 46.5 mmol) was added slowly and the reaction mixture was heated to r.t., then stirred for 48 h. The solvent was removed in vacuo and the resulting solid was dissolved in a mixture of 10% MeOH in DCM. The solution was filtered through a celite pad to remove insoluble material. The filtrate was then concentrated and purified by silica gel chromatography (acetone/DCM 1:30) to offer the product (5.5 g, 53%). **^{1}H NMR** (500 MHz, $CDCl_3$) δ 9.33 (s, 1H), 8.60 (s, 1H), 8.06 (m, 1H), 7.98 (m, 1H), 7.77 (m, 2H), 4.06 (s, 3H). **^{13}C NMR** (126 MHz, DMSO-*d*$_6$) δ 165.7, 152.7, 141.1,

134.9, 131.5, 130.0, 129.5, 128.1, 127.7, 123.6, 52.4. **HRMS** (ESI+) calcd. for $C_{11}H_9NO_2$ $[M+Na]^+$ 210.0525, found 210.0525.

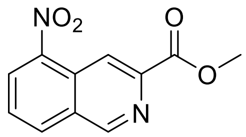

Compound 17. Compound **16** (4.72 g, 25.2 mmol) was dissolved in concentrated H_2SO_4 (47 mL) and the solution was cooled to 0 °C. $NaNO_3$ (2.36 g, 27.8 mmol) was added slowly while maintaining the temperature below 5 °C. The reaction mixture was stirred at r.t. for 2 h and then poured slowly into ice water. The pH was adjusted to 7 by adding saturated $NaHCO_3$ solution and the aqueous phase was extracted by a mixture of 10% MeOH in DCM (3 x 200 mL). The combined organic layers were collected, dried over $MgSO_4$, filtered and concentrated. The residue was washed with diethyl ether to afford the product as a yellow solid (4.67 g, 80%). **^{1}H NMR** (500 MHz, DMSO-*d*$_6$) δ 9.65 (s, 1H), 9.11 (s, 1H), 8.78 (d, *J* = 7.6 Hz, 1H), 8.72 (d, *J* = 8.1 Hz, 1H), 8.06 (t, *J* = 7.9 Hz, 1H), 3.97 (s, 3H). **^{13}C NMR** (126 MHz, DMSO-*d*$_6$) δ 165.6, 154.2, 145.2, 144.3, 135.9, 130.3, 130.2, 129.6, 127.7, 118.7, 53.3. **HRMS** (ESI+) calcd. for $C_{11}H_8N_2O_4$ $[M+Na]^+$ 255.0376, found 255.0377.

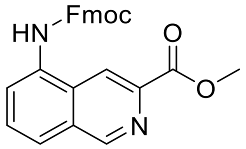

Compound 19. Compound **17** (2.27 g, 9.8 mmol) was dissolved in dry MeOH (30 mL) and dry DMF (30 mL) under N_2. Then Pd/C (227 mg) was added and the N_2 was replaced by H_2. The reaction mixture was stirred overnight under H_2 atmosphere, then filtered and concentrated. The product **18** was obtained with a quantitative yield and used directly in the next step. Compound **18** (1.5 g, 7.4 mmol) was dissolved in 1,4-dioxane (200 mL) and $NaHCO_3$ solution (5%, w/w, 30 mL). The reaction mixture was cooled to 0 °C. A solution of Fmoc-Cl (2.11 g, 8.2 mmol) in 1,4-dioxane (50 mL) was added dropwise at 0 °C and the reaction mixture was stirred at r.t. overnight. The pH was then adjusted to 4 by adding 5% citric acid solution. The aqueous phase was extracted with DCM (3 x 100 mL). The combined organic phases were collected, dried over $MgSO_4$, filtered and concentrated. The residue was washed with diethyl ether to afford the product as a white solid. (1.45 g, 78%).**^{1}H NMR** (500 MHz, DMSO-*d*$_6$) δ 10.14 (s, 1H), 9.40 (s, 1H), 8.79 (s, 1H), 8.04 (d, *J* = 8.1 Hz, 1H), 7.92 (d, *J* = 7.5 Hz, 3H), 7.79 (m, 3H), 7.43 (t, *J* = 7.5 Hz, 2H), 7.35 (t, *J* = 7.5 Hz, 2H), 4.53 (d, *J* = 6.9 Hz, 2H), 4.37 (t, *J* = 6.9 Hz, 1H), 3.95 (s, 3H). **^{13}C NMR** z(126 MHz, DMSO-*d*$_6$) δ 165.7, 154.6, 152.9, 143.8, 140.9, 140.8, 134.4, 130.0, 129.9, 129.4, 127.8, 127.2, 125.4, 125.3, 124.3, 120.2, 118.9, 66.2, 52.5, 46.6. **HRMS** (ESI+) calcd. for $C_{26}H_{20}N_2O_4$ $[M+H]^+$ 425.1496, found 425.1492.

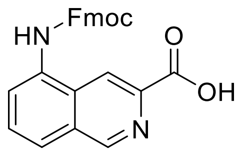

Compound 20. Compound **19** (2.3 g, 5.4 mmol) and LiI (3.6 g, 26.8 mmol) were suspended in degassed EtOAc (40 mL) under N_2. The reaction mixture was refluxed overnight. The solvent was removed in vacuo and the remaining solid was washed with aqueous citric acid solution (5%, w/w), then with aqueous $Na_2S_2O_3$ solution (5%, w/w) and finally with water. The product was obtained after drying in vacuo (1.75 g, 79%). **^{1}H NMR** (500 MHz, DMSO-*d*$_6$) δ 13.10 (bs, 1H), 10.12 (s, 1H), 9.40 (s, 1H), 8.80 (s, 1H), 8.04 (d, *J* = 8.1 Hz, 1H), 7.95-7.87 (m, 3H), 7.82-7.75 (m, 3H), 7.43 (t, *J* = 7.4 Hz, 2H), 7.35 (t, *J* = 7.4 Hz, 2H), 4.51 (d, *J* = 7.0 Hz, 2H), 4.36 (t, *J* = 7.0 Hz, 1H). **^{13}C NMR** (126 MHz, DMSO-*d*$_6$) δ 166.7, 154.6,

152.6, 143.8, 141.9, 140.8, 134.4, 129.9, 129.7, 129.6, 127.8, 127.2, 125.3, 124.3, 120.3, 118.6, 66.3, 48.6, 46.6. **HRMS** (ESI+) calcd. for $C_{25}H_{18}N_2O_4$ $[M+H]^+$ 411.1339, found 411.1337.

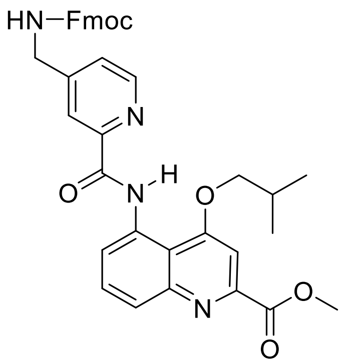

Compound 21. Compound **10** (1 g, 2.35 mmol) was dissolved in dry MeOH (7 mL) under N_2 atmosphere. Then Pd/C (38 mg) was added and N_2 was replaced by H_2. The reaction was stirred at r.t. overnight under H_2 atmosphere. After completion of the reaction, which was confirmed by TLC, the reaction mixture was filtered and concentrated. Compound **11** was obtained quantitatively and used directly in the next step. Compound **11** (506 mg, 1.85 mmol), compound **14** (574 mg, 1.53 mmol) and PyBOP (1.6 g, 3.07 mmol) were dissolved in dry $CHCl_3$ under N_2. DIPEA (1.1 mL, 6.34 mmol) was added and the reaction mixture was stirred at r.t. for 48 h. The solution was diluted with $CHCl_3$, and washed with citric acid solution (5%, w/w), then with $NaHCO_3$ solution (5%, w/w). The organic phases were combined and concentrated. The product was precipitated from diethyl ether as a pale-yellow solid (830 mg, 86%). **^{1}H NMR** (500 MHz, DMSO-d_6) δ 12.36 (s, 1H), 8.97 (dd, *J* = 5.9, 3.1 Hz, 1H), 8.67 (d, *J* = 4.9 Hz, 1H), 8.16 (s, 1H), 8.11 (t, *J* = 6.1 Hz, 1H), 7.93–7.83 (m, 4H), 7.71 (d, *J* = 7.5 Hz, 2H), 7.63 (d, *J* = 3.1 Hz, 1H), 7.52 (d, *J* = 4.6 Hz, 1H), 7.42 (t, *J* = 7.4 Hz, 2H), 7.34 (t, *J* = 7.4 Hz, 2H), 4.40 (d, *J* = 6.8 Hz, 2H), 4.36 (d, *J* = 6.0 Hz, 2H), 4.33–4.29 (m, 2H), 4.26 (t, *J* = 6.8 Hz, 1H), 3.96 (s, 3H), 2.58 (nonet, *J* = 6.8 Hz, 1H), 1.00 (d, *J* = 6.6 Hz, 6H). (Mixture of two conformers with a ratio of 1:0.15, only the data of the major species is listed here). **^{13}C NMR** (126 MHz, DMSO-d_6) δ 165.3, 163.4, 162.3, 156.5, 151.5, 149.6, 149.5, 148.7, 148.3, 143.9, 140.8, 134.5, 130.8, 127.7, 127.1, 125.7, 125.4, 125.2, 120.5, 120.2, 118.2, 113.1, 102.1, 76.6, 65.6, 52.8, 46.8, 42.9, 26.9, 19.1. **HRMS** (ESI+) calcd. for $C_{37}H_{34}N_4O_6$ $[M+H]^+$ 631.2557, found 631.2542.

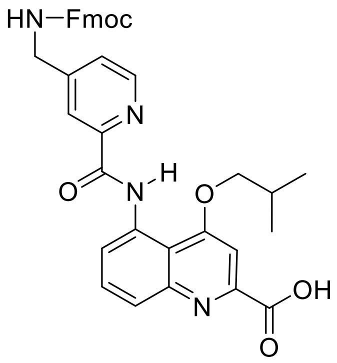

Compound 22. Compound **21** (1.08 g, 1.71 mmol) was suspended in degassed EtOAc under N_2. Then LiI (1.15 g, 8.60 mmol) was added and the mixture was refluxed overnight. The solvent was removed in vacuo and the remaining residue was washed with 5% aqueous citric acid solution, then 5% aqueous $Na_2S_2O_3$ solution and water. The product was obtained as a pale-yellow solid after drying in vacuo (800 mg, 76%). **^{1}H NMR** (500 MHz, DMSO-d_6) δ 12.42 (s, 1H), 11.57 (s, 1H), 8.70–8.65 (m, 2H), 8.10–8.07 (m, 2H), 7.90 (d, *J* = 7.5 Hz, 2H), 7.76–7.69 (m, 4H), 7.57 (t, *J* = 8.2 Hz, 1H), 7.45–7.38 (m, 2H), 7.34 (td, *J* = 7.4, 1.3 Hz, 2H), 6.52 (s, 1H), 4.41–4.24 (m, 5H), 4.20–4.14 (m, 2H), 2.58 (nonet, *J* = 6.8, 1H), 1.03–1.01 (m, 6H). **^{13}C NMR** (126 MHz, DMSO-d_6) δ 164.1, 162.3, 156.5, 151.5, 149.5, 148.2, 148.1, 143.9, 140.8, 139.5, 137.5, 134.4, 129.0, 127.7, 127.3, 127.1, 125.2, 121.4, 120.2, 120.1, 112.6, 109.9, 101.5, 76.6, 65.6, 46.8, 42.9, 26.8, 19.0. **HRMS** (ESI+) calcd. for $C_{36}H_{32}N_4O_6$ $[M+H]^+$ 617.2395, found 617.2386.

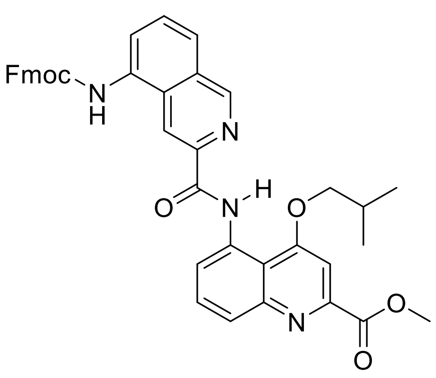

Compound 23. Compound **10** (1 g, 2.35 mmol) was dissolved in dry MeOH (7 mL) under N_2 atmosphere. Then Pd/C (38 mg) was added and N_2 was replaced by H_2. The reaction was stirred at r.t. overnight under H_2 atmosphere. After completion of the reaction, which was confirmed by TLC, the reaction mixture was filtered and concentrated. Compound **11** was obtained quantitatively and used directly in the next step. Compound **20** (1.3 g, 3.2 mmol) was dissolved in thionyl chloride (13 mL) under N_2. DMF (0.1 mL) was added and the reaction mixture was stirred at r.t. for 2 h. Thionyl chloride and DMF were removed in vacuo overnight to afford the desired acyl chloride. To compound **11** (690 mg, 2.5 mmol) and DIPEA (1.3 mL, 13.6 mmol) in dry $CHCl_3$ (20 mL) was added dropwise a solution of previously prepared acyl chloride of **20** in dry $CHCl_3$ (20 mL) under N_2. The reaction mixture was stirred overnight at r.t. and the solvent was then removed in vacuo. The resulting crude was purified by silica gel chromatography (cyclohexane/EtOAc 1:1). The product was recrystallized from acetonitrile/DCM to obtain the product as a pale yellow solid (800 mg, 48%). **^{1}H NMR** (500 MHz, $CDCl_3$) δ 12.56 (s, 1H), 9.20 (s, 1H), 9.11 (d, *J* = 7.7 Hz, 1H), 8.88 (s, 1H), 8.33 (bs, 1H), 8.04 (d, *J* = 8.8 Hz, 1H), 7.88 (d, *J* = 8.1 Hz, 1H), 7.84–7.78 (m, 3H), 7.75 (t, *J* = 7.9 Hz, 1H), 7.69-7.63 (m, 3H), 7.53 (bs, 1H), 7.42 (t, *J* = 7.4 Hz, 2H), 7.34 (t, *J* = 7.4 Hz, 2H), 4.63 (d, *J* = 6.5 Hz, 2H), 4.33 (t, *J* = 6.5 Hz, 1H), 4.27 (d, *J* = 7.1 Hz, 2H), 4.10 (s, 3H), 2.76 (nonet, *J* = 7.0 Hz, 1H), 1.12 (d, *J* = 6.7 Hz, 6H). **^{13}C NMR** (126 MHz, $CDCl_3$) δ 166.1, 164.0, 163.5, 154.0, 151.3, 150.4, 148.6, 144.3, 143.7, 141.5, 134.9, 133.9, 130.8, 130.3, 129.7, 128.0, 127.3, 126.7, 125.1, 123.7, 120.2, 119.5, 114.6, 114.2, 102.0, 67.8, 53.5, 47.2, 27.5, 19.7. **HRMS** (ESI+) calcd. for $C_{40}H_{34}N_4O_6$ $[M+H]^+$ 667.2551, found 667.2539.

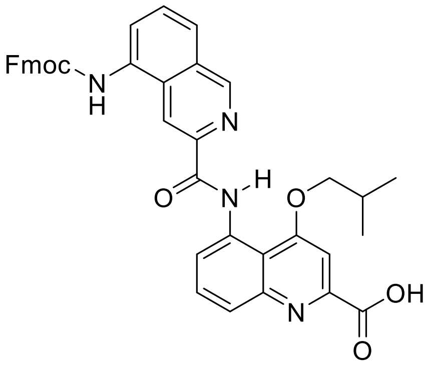

Compound 24. Compound **23** (600 mg, 0.9 mmol) and LiI (1.2 g, 9.0 mmol) were suspended in degassed EtOAc (50 mL) under N_2 and the reaction mixture was refluxed overnight. The solvent was removed in vacuo. The remaining residue was washed with citric acid solution (5%, w/w), $Na_2S_2O_3$ solution (5%, w/w) and water. The product was obtained as a pale yellow solid after drying in vacuo (453 mg, 77%). **^{1}H NMR** (500 MHz, DMSO-*d*$_6$) δ 12.57 (s, 1H), 10.22 (s, 1H), 9.47 (s, 1H), 9.04 (dd, *J* = 6.1, 3.0 Hz, 1H), 8.95 (s, 1H), 8.16 (d, *J* = 8.3 Hz, 1H), 7.95–7.78 (m, 8H), 7.65 (s, 1H), 7.44 (t, *J* = 7.4 Hz, 2H), 7.37 (t, *J* = 7.4 Hz, 2H), 4.54 (d, *J* = 7.0 Hz, 2H), 4.37 (dd, *J* = 12.4, 7.0 Hz, 3H), 2.65 (nonet, *J* = 6.8 Hz, 1H), 1.04 (d, *J* = 6.6 Hz, 6H). **^{13}C NMR** (126 MHz, DMSO-*d*$_6$) δ 166.1, 163.5, 162.7, 154.7, 151.6, 149.9, 149.3, 143.8, 143.0, 140.8, 134.7, 134.5, 130.7, 130.2, 130.1, 129.5, 127.8, 127.2, 125.3, 125.3, 124.8, 120.2, 118.1, 116.3, 113.0, 102.0, 76.5, 66.3, 46.6, 26.8, 19.1. **HRMS** (ESI+) calcd. for $C_{39}H_{32}N_4O_6$ $[M+H]^+$ 653.2395, found 653.2385.

5.3 Solid phase synthesis general methods

5.3.1 Loading of the resin via HBTU activation

SASRIN resin (500 mg, 0.4–0.6 mmol, 1 eq.) was swollen in 5 mL DCM for 1 h, transferred to the microwave vessel and washed 3 times with extra dry DMF. DIPEA (170 µL, 1.0 mmol, 2 eq.) was added to a mixture of Fmoc-$Q^{B/D/M}$-OH (0.45 mmol, 0.9 eq.) and HBTU (228 mg, 1.2 eq.) in extra dry DMF (5 mL). The resulting solution was shaken for 30 s before to be poured to the resin-containing reaction vessel. The reaction mixture was subjected to treatment in a microwave oven (50 °C, 20 min, 25 W). The resin was filtered and washed with DMF (5 x 2 mL) and DCM (10 x 2 mL). Capping was performed by adding a mixture of DCM/pyridine/benzoyl chloride (3:1:1, v/v/v, 5 mL) to the resin followed by shaking for 30 min at r.t., and subsequent washing with DCM (20 x 2 mL). To monitor the efficiency of the 1st loading, a small amount of resin (around 2 mg) was taken and dried in vacuo. The loading was estimated at this scale.

HMBA-AM resin (500 mg, 0.8–1.2 mmol, 1 eq.) was swollen in 5 mL DCM for 1 h, transferred to the microwave vessel and washed 3 times with extra dry DMF. DIPEA (170 µL, 1.0 mmol, 2 eq.) was added to a mixture of Fmoc-Gly-OH (134 mg, 0.45 mmol, 0.9 eq.) and HBTU (228 mg, 1.2 eq.) in extra dry DMF (5 mL) and the resulting solution was shaken for 30 s before being poured into the resin-containing reaction vessel. The reaction mixture was subjected to a treatment in a microwave oven (50 °C, 20 min, 25 W). The resin was filtered and washed with DMF (5 x 2 mL) and DCM (10 x 2 mL). Capping was performed by adding a mixture of DCM/pyridine/benzoyl chloride (3:1:1, v/v/v, 5 mL) to the resin followed by shaking for 30 min at r.t., and subsequent washing with DCM (20 x 2 mL). To monitor the efficiency of the 1st loading, a small amount of resin (around 2 mg) was taken and dried in vacuo. The loading was estimated at this scale.

5.3.2 Estimation of the loading

To a small amount of Fmoc-Gly-HMBA AM resin or Fmoc- $Q^{B/D/M}$-SASRIN resin (1–2 mg), a freshly prepared solution of DMF/piperidine (8:2 (v/v), 3.0 mL) was added. The mixture was shaken and incubated for 5 min. Then the absorption was measured at 301 nm using a NanoDrop One Microvolume UV-Vis Spectrophotometer and a Hellma quartz glass cuvette 104 (path length 10 mm). Three replicates were measured, then the loading was calculated with the following equation:

$$\text{loading}\ \left(\text{in}\ \frac{\text{mmol}}{\text{g}}\right) = \frac{\text{Abs}_{301\ \text{nm}} \times \text{V}}{\varepsilon_{301\text{nm}} \times l \times \text{m}}$$

$$\varepsilon_{301\text{nm}} = 7800\ \text{L/mol/cm}$$ [13]

5.3.3 Solid phase synthesis of monomer and turn units via *in-situ* activation

For manual solid phase synthesis under MW-irradiation: The Fmoc- $Q^{B/D/M}$-SASRIN resin was swallowed in DCM (5 mL) for 2 h, and then transferred into the reaction vessel followed by washing with NMP (3 x 3 mL). The deprotection of the Fmoc group was performed by using a solution of 2% DBU in NMP (3 mL, 3 min + 7 min). The

resin was next filtered off and washed with DCM (3 x 2 mL) and then with anhydrous THF (5 x 2 mL). The Fmoc deprotection step was performed before each aromatic monomer coupling. The resin was then suspended in anhydrous THF (1 mL) and 2,3,5-collidine (5 eq. with respect to the resin-loading) was added to the resin supernatant. The Fmoc-protected monomer or turn unit (2 eq. with respect to the resin-loading) and PPh_3 (4 eq. with respects to the resin-loading) were successively added in a vial to be solubilized in freshly distilled $CHCl_3$ (1 mL). Trichloroacetonitrile (4.5 eq. with respect to the resin loading) was then added to the vial and the resulting acid chloride solution was shaken for 30 s before to be poured to the resin-containing reaction vessel. The reaction vessel was then placed in the microwave oven and subjected to MW irradiation for 15 min (50 °C, 50 W). The resin was then washed 3 times with anhydrous THF. This entire coupling step was then repeated once more. For the final coupling of (1*S*)-camphanic acid, the resin was suspended in anhydrous THF (1 mL) and 2,3,5-collidine (5 eq. with respect to the resin-loading) was added to the resin suspensions. A solution of (1*S*)-camphanyl chloride (2 eq. with respect to the resin-loading, purchased from Sigma-Aldrich, 98%, ee: 99%) in freshly distilled $CHCl_3$ (1 mL) was added to the supernatant and the resin was shaken at r.t. for 2 h. The resin was filtered off, and washed 3 times with dry THF, and the same process was repeated once. After coupling, the resin was vigorously washed 3 times with DMF and 3 times with DCM.

The fully automatic solid phase synthesis on Chorus PurePep ® synthesizer followed previously reported procedures.[8]

5.3.4 Fragment condensation via BOP activation

The corresponding resin (cal. 100 mg) was swallowed in DCM (5 mL) for 2 h. The deprotection of the Fmoc group was performed by using a solution of 2% DBU/NMP (3 mL, 3 min + 7 min). The resin was next filtered off and washed with DCM (3 x 2 mL) and then with anhydrous THF (5 x 2 mL). DIPEA (2 eq. with respects to the resin-loading) was added to a mixture of Fmoc-protected fragment sequences (1 eq. with respects to the resin-loading) and BOP (1.5 eq. with respects to the resin-loading) in anhydrous chloroform (5 mL) under N_2. After activating for 1 min, the solution was added to the resin. The supernatant was shaken at r.t. overnight. The resin was filtered and washed with anhydrous chloroform (3 x 3 mL). To perform a capping step, the resin was suspended in anhydrous chloroform (3 mL) and DIPEA (4 eq. with respects to the resin-loading) was added. Acetyl chloride (2 eq. with respects to the resin-loading) was added to the supernatant and the resin was shaken at r.t. for 30 min. The resin was filtered and washed with DCM (3 x 2 mL), 20% DIPEA/NMP (3 x 2 mL) and DCM (10 x 2 mL).

5.3.5 Mini-cleavage

For SASRIN resin: To perform a mini cleavage, the resin (1–2 mg) was swollen in 1 mL TFA/DCM (1:99, v/v) solution and incubated at r.t. for 10 min. The DCM solution was washed with aqueous $NaHCO_3$ solution and the solvent was removed under reduced pressure.

For HMBA AM resin: To perform a mini cleavage, the resin (1–2 mg) was swollen in 1 mL MeOH/DCM (1:1, v/v) solution followed by the addition of 10 µL NaOMe (25% (m/m)) and incubated at r.t. for 10 min. The cleavage

solution was diluted with DCM, washed with aqueous citric acid solution (5%), dried over $MgSO_4$, filtered and the solvent was finally removed under reduced pressure.

5.3.6 Full cleavage[14,15]

For SASRIN resin: A mixture of 1% TFA/DCM (2 mL) was added to the resin and stirred at r.t. for 1 min (x 10). The solution was directly quenched by sat. $NaHCO_3$ solution. The organic phase was collected, dried over $MgSO_4$, filtered and concentrated to afford the crude product as a yellow solid.

For HMBA AM resin: The resin (around 100 mg) was dried in vacuo and slowly added to 400 mL cleavage solution (preparation see below) under N_2 atmosphere. The mixture was stirred under N_2 atmosphere for 2 h before it was added to 100 mL of aqueous citric acid solution (5%). The aqueous layer was extracted with DCM (3 x 50 mL). The combined organic phases were washed with brine, dried over $MgSO_4$, filtered and the solvent was evaporated under reduced pressure. The crude product was obtained as a solid.

Preparation of the cleavage solution: 200 mL dry MeOH was added to 200 mL dry DCM under N_2 atmosphere. 2 mL NaOMe (25% (m/m)) in methanol were added and the mixture was mixed well by magnetic stirring. A sufficient amount of cleavage solution (at least 400 mL cleavage solution per 100 mg resin) was important to avoid the formation of oligomeric acid as the by-product.

5.4 Synthesis of oligomers

O_2N-Q^B<u>X</u>Q^B-T6f-$Q^BQ^MQ^B$-OMe (**2a**) Compound **2a** was synthesized on SASRIN resin (scale: 15 µmol) using SPFS procedure reported in paragraph 5.3. The crude product obtained after full-cleavage was dried in vacuo and dissolved in dry chloroform/MeOH 3:2 (3 mL) under N_2. To this solution $TMSCHN_2$ (2 M in *n*-hexane, 17.7 µL, 60 µmol) was added dropwise. The reaction mixture was stirred at r.t. for 2 h. A few drops of acetic acid were then added and stirring continued for 30 min at r.t. to quench the excess amounts of $TMSCHN_2$. Then the solution was diluted with DCM, washed with aqueous $NaHCO_3$ solution, dried over $MgSO_4$, filtered and concentrated. The crude product was purified by RP-HPLC. Compound **2a** was obtained as a yellow solid (7.2 mg, 26%). **^{1}H NMR** (500 MHz, $CDCl_3$) δ 12.58 (s, 1H), 12.33 (s, 1H), 12.15 (s, 1H), 12.03 (s, 1H), 12.01 (s, 1H), 12.00 (s, 1H), 9.10 (dd, *J* = 7.6, 1.4 Hz, 1H), 8.96 (dd, *J* = 7.6, 1.3 Hz, 1H), 8.93 (dd, *J* = 7.7, 1.3 Hz, 1H), 8.69 (dd, *J* = 7.9, 1.2 Hz, 1H), 8.50 (dd, *J* = 8.3, 1.5 Hz, 1H), 8.31 (dd, *J* = 7.5, 1.3 Hz, 1H), 8.17–8.16 (m, 1H), 8.08 (dd, *J* = 7.8, 1.3 Hz, 2H), 8.03–7.96 (m, 7H), 7.87 (dd, *J* = 8.3, 1.3 Hz, 1H), 7.79 (s, 1H), 7.78–7.71 (m, 2H), 7.66–7.61 (m, 2H), 7.59–7.55 (m, 1H), 7.44 (s, 1H), 7.39 (dd, *J* = 8.4, 7.5 Hz, 1H), 7.29–7.25 (m, 1H), 7.15–7.13 (m, 1H), 6.94 (s, 1H), 6.90 (s, 1H), 6.68 (s, 1H), 6.59 (t, *J* = 8.1 Hz, 1H), 6.35 (dd, *J* = 8.3, 1.2 Hz, 1H), 4.37–4.19 (m, 8H), 3.98 (s, 3H), 3.89 (d, *J* = 6.5 Hz, 2H), 3.86 (d, *J* = 6.1 Hz, 2H), 3.52 (s, 3H), 2.69 (nonet, *J* = 6.9 Hz, 1H), 2.43 (m, 2H), 2.30 (tt, *J* = 13.4, 6.6 Hz, 2H), 1.70 (s, 9H), 1.35–1.10 (m, 30H). **HRMS** (ESI+) calcd. for $C_{103}H_{100}N_{16}O_{18}$ $[M+Na]^+$, 1871.7294, found 1871.7320.

O_2N-Q^BXQ^B-T6f-$Q^BQ^MQ^B$-OMe (**2b**) Compound **2a** (7.2 mg, 3.8 µmol) was treated with TFA/DCM 1:1 (3 mL) at r.t. for 2 h. The solvent was removed in vacuo. The residue was washed with diethyl ether, yielding the product as a yellow solid (7.0 mg, quant.). **^{1}H NMR** (500 MHz, $CDCl_3$) δ 12.62 (s, 1H), 12.59 (s, 1H), 11.96 (s, 1H), 11.90 (s, 2H), 11.79 (s, 1H), 10.24 (s, 1H), 9.19 (d, *J* = 7.5 Hz, 1H), 8.80 (d, *J* = 7.5 Hz, 1H), 8.68 (d, *J* = 6.9 Hz, 1H), 8.64 (d, *J* = 7.9 Hz, 1H), 8.50

(dd, *J* = 8.4, 1.5 Hz, 1H), 8.30 (d, *J* = 7.5 Hz, 2H), 8.19–8.04 (m, 5H), 7.99–7.92 (m, 4H), 7.85–7.80 (m, 4H), 7.75 (q, *J* = 7.6 Hz, 2H), 7.65–7.60 (m, 3H), 7.47 (d, *J* = 1.5 Hz, 1H), 7.38 (t, *J* = 7.9 Hz, 1H), 7.33 (t, *J* = 7.9 Hz, 1H), 6.90 (s, 1H), 6.69 (s, 1H), 6.61 (t, *J* = 8.1 Hz, 1H), 6.37 (d, *J* = 7.0 Hz, 1H), 4.77–4.62 (m, 2H), 4.44 (s, 3H), 4.38–4.33 (m, 1H), 4.23 (m, 4H), 4.00–3.85 (m, 5H), 3.58 (s, 3H), 2.50–2.29 (m, 5H), 1.36–1.13 (m, 30H). **HRMS** (ESI+) calcd. for $C_{99}H_{92}N_{16}O_{18}$ $[M+H]^+$ 1793.6848, found 1793.6892.

O_2N-Q^{B}X̲Q^B-T6r-Q^{B}Q^{M}Q^B-OMe (**3a**) Compound **3a** was synthesized on SASRIN resin (scale: 15 µmol) using SPFS procedure reported in paragraph 5.3. The crude after full-cleavage was dried in vacuo and dissolved in dry chloroform/MeOH 3:2 (3 mL) under N_2. $TMSCHN_2$ (2 M in *n*-hexane, 17.7 µL, 60 µmol) was added dropwise. The solution was stirred at r.t. for 2 h. A few drops of acetic acid were added and stirred at r.t. for 30 min to quench the excess $TMSCHN_2$. Then the solution was diluted with DCM, washed with aqueous $NaHCO_3$ solution. dried over $MgSO_4$, filtered and concentrated. The crude was purified by RP-HPLC. The compound **3a** was obtained as a yellow solid (13.2 mg, 47%). **^{1}H NMR** (500 MHz, $CDCl_3$) δ 12.61 (s, 1H), 12.12 (s, 1H), 12.09 (s, 1H), 12.04 (s, 1H), 12.03 (s, 1H), 11.75 (s, 1H), 10.73 (s, 1H), 9.24 (dd, *J* = 7.6, 1.3 Hz, 1H), 9.14–9.12 (m, 2H), 8.56 (dd, *J* = 8.3, 1.5 Hz, 1H), 8.40 (dd, *J* = 7.9, 1.1 Hz, 2H), 8.37 (s, 1H), 8.33 (dd, *J* = 7.5, 1.4 Hz, 1H), 8.15 (dd, *J* = 7.6, 1.3 Hz, 1H), 8.11–8.06 (m, 2H), 8.05–8.00 (m, 3H), 7.93 (s, 1H), 7.91 (dd, *J* = 8.3, 1.3 Hz, 1H), 7.83–7.77 (m, 3H), 7.74 (t, *J* = 7.8 Hz, 1H), 7.67 (dd, *J* = 7.4, 1.5 Hz, 1H), 7.62–7.59 (m, 1H), 7.55 (s, 1H), 7.47–7.43 (m, 1H), 7.35–7.29 (m, 3H), 7.16–7.12 (m, 2H), 6.80 (s, 1H), 6.70 (s, 1H), 6.59 (t, *J* = 8.0 Hz, 1H), 6.38 (dd, *J* = 8.3, 1.1 Hz, 1H), 4.49 (d, *J* = 6.0 Hz, 2H), 4.27–4.14 (m, 4H), 3.99 (d, *J* = 6.1 Hz, 2H), 3.87 (d, *J* = 6.0 Hz, 2H), 3.80 (s, 3H), 3.53 (s, 3H), 3.03–2.94 (m, 1H), 2.51–2.41 (m, 2H), 2.39–2.31 (m, 2H), 1.45 (d, *J* = 6.6 Hz, 6H), 1.29 (d, *J* = 6.5 Hz, 6H), 1.26 (d, *J* = 6.8 Hz, 12H), 1.21 (d, *J* = 6.8 Hz, 6H), 1.10 (s, 9H). **HRMS** (ESI+) calcd. for $C_{106}H_{100}N_{16}O_{18}$ $[M+Na]^+$ 1907.7294, found (HR-ESI) 1907.7255.

O_2N-Q^{B}XQ^B-T6r-Q^{B}Q^{M}Q^B-OMe (**3b**) Compound **3a** (13.2 mg, 7.0 µmol) was treated with a TFA/DCM 1:1 (3 mL) at r.t. for 2 h. The solvent was removed in vacuo. The residue was washed with diethyl ether, yielding the products as a yellow solid (12.8 mg, quant.). **^{1}H NMR** (500 MHz, $CDCl_3$) δ 12.60 (s, 1H), 12.57 (s, 1H), 12.30 (s, 1H), 11.83 (s, 1H), 11.75 (s, 1H), 11.66 (s, 1H), 11.37 (s, 1H), 10.15 (s, 1H), 9.22 (d, *J* = 7.4 Hz, 1H), 9.01 (d, *J* = 7.4 Hz, 1H), 8.75 (s, 1H), 8.58 (dd, *J* = 8.3, 1.5 Hz, 1H), 8.47 (d, *J* = 7.9 Hz, 2H), 8.40–8.35 (m, 2H), 8.22–8.15 (m, 2H), 8.12–8.07 (m, 2H), 8.02–7.98 (m, 2H), 7.95 (dd, *J* = 8.3, 1.4 Hz, 1H), 7.90 (s, 1H), 7.86 (s, 1H), 7.86–7.77 (m, 4H), 7.70 (t, *J* = 7.9 Hz, 1H), 7.66 (s, 1H), 7.64–7.61 (m, 3H), 7.43–7.39 (m, 1H), 7.35 (t, *J* = 7.9 Hz, 2H), 6.84 (s, 1H), 6.70 (s, 1H), 6.65 (t, *J* = 7.9 Hz, 1H), 6.40 (d, *J* = 7.9 Hz, 1H), 4.63–4.59 (m, 1H), 4.57 (s, 3H), 4.42–4.38 (m, 1H), 4.27–4.19 (m, 4H), 3.98–3.85 (m, 4H), 3.57 (s, 3H), 2.91–2.82 (m, 1H), 2.57–2.48 (m, 1H), 2.48–2.41 (m, 1H), 2.40–2.31 (m, 2H), 1.38 (d, *J* = 6.8 Hz, 3H), 1.33 (d, *J* = 6.8 Hz, 6H), 1.31 (d, *J* = 6.6 Hz, 3H), 1.28 (d, *J* = 6.8 Hz, 3H), 1.26 (d, *J* = 2.4 Hz, 3H), 1.25 (d, *J* = 2.3 Hz, 6H), 1.23 (d, *J* = 1.8 Hz, 3H), 1.22 (d, *J* = 6.8 Hz, 3H). **HRMS** (ESI+) calcd. for $C_{102}H_{92}N_{16}O_{18}$ $[M+H]^+$ 1829.6848, found 1829.6872.

C*-Q^{B}X̲Q^B-T6r-Q^{B}Q^{M}Q^B-OMe (**4a**) Compound **4a** was synthesized on SASRIN resin (scale: 15 µmol) using SPFS procedures reported in paragraph 5.3. The crude after full-cleavage was dried in vacuo and dissolved in dry chloroform/MeOH 3:2 (3 mL) under N_2. $TMSCHN_2$ (2 M in *n*-hexane, 17.7 µL, 60 µmol) was added dropwise. The solution was stirred at r.t. for 2 h. A few drops of acetic acid were added and stirring continued at r.t. for 30 min to

quench the excess amounts of $TMSCHN_2$. Then the solution was diluted with DCM, washed with aqueous $NaHCO_3$ solution, dried over $MgSO_4$, filtered and concentrated. The crude product was purified by RP-HPLC. The product was obtained as a yellow solid (13.2 mg, 43%). **^{1}H NMR** (500 MHz, $CDCl_3$) δ 12.61 (s, 1H), 12.41 (s, 1H), 12.08 (s, 1H), 12.05 (s, 1H), 12.03 (s, 1H), 11.95 (s, 1H), 10.77 (s, 1H), 9.99 (s, 1H), 9.12 (t, *J* = 7.5 Hz, 2H), 9.05 (s, 1H), 8.76 (d, *J* = 7.5 Hz, 1H), 8.49 (d, *J* = 7.8 Hz, 1H), 8.38 (s, 1H), 8.34 (d, *J* = 7.6 Hz, 1H), 8.16 (d, *J* = 6.5 Hz, 1H), 8.04–7.98 (m, 4H), 7.96 (d, *J* = 8.4 Hz, 1H), 7.92–7.89 (m, 2H), 7.86 (t, *J* = 7.8 Hz, 1H), 7.83–7.81 (m, 2H), 7.78 (t, *J* = 7.9 Hz, 1H), 7.70 (t, *J* = 7.9 Hz, 1H), 7.66 (s, 1H), 7.61 (t, *J* = 7.9 Hz, 1H), 7.56 (s, 1H), 7.32–7.11 (m, 4H), 6.93 (s, 1H), 6.81 (s, 1H), 6.70 (s, 1H), 6.61 (t, *J* = 8.0 Hz, 1H), 6.39 (d, *J* = 7.4 Hz, 1H), 4.56 (s, 1H), 4.47 (t, *J* = 8.5 Hz, 1H), 4.39 (t, *J* = 7.1 Hz, 1H), 4.34–4.27 (m, 1H), 4.26–4.19 (m, 1H), 4.18–4.11 (m, 1H), 3.99–3.93 (m, 2H), 3.90–3.84 (m, 2H), 3.80 (s, 2H), 3.73 (s, 1H), 3.69 (t, *J* = 4.8 Hz, 1H), 3.65 (s, 3H), 3.53 (s, 3H), 2.57–1.98 (m, 5H), 1.37–1.21 (m, 42H), 0.94 (s, 6H). **HRMS** (ESI+) calcd. for $C_{116}H_{114}N_{16}O_{19}$ $[M+H]^+$ 2035.8519, found 2035.8703.

C*-Q^BXQ^B-T6r-$Q^BQ^MQ^B$-OMe (**4b**) Compound **4a** (13.2 mg, 6.5 µmol) was treated with TFA/DCM 1:1 (3 mL) at r.t. for 2 h. The solvent was removed in vacuo. The residue was washed with diethyl ether, yielding the product as a yellow solid (12.8 mg, quant.). **^{1}H NMR** (500 MHz, $CDCl_3$) δ 12.66 (s, 1H), 12.60 (s, 1H), 12.51 (s, 1H), 11.95 (s, 1H), 11.88 (s, 1H), 11.76 (s, 1H), 11.33 (s, 1H), 10.15 (s, 1H), 10.01 (s, 1H), 9.19 (d, *J* = 6.9 Hz, 1H), 8.93–8.89 (m, 1H), 8.74 (s, 1H), 8.54 (dd, *J* = 5.9, 2.7 Hz, 1H), 8.44 (s, 1H), 8.31 (ddd, *J* = 9.8, 7.8, 1.3 Hz, 2H), 8.20–8.16 (m, 2H), 8.12 (dd, *J* = 8.3, 1.3 Hz, 1H), 8.05–7.94 (m, 6H), 7.87–7.81 (m, 4H), 7.72 (dt, *J* = 13.3, 8.1 Hz, 2H), 7.66 (s, 1H), 7.59 (s, 1H), 7.56 (s, 1H), 7.47 (s, 1H), 7.35 (t, *J* = 8.0 Hz, 2H), 7.31–7.27 (m, 1H), 6.78 (s, 1H), 6.71 (s, 1H), 6.59 (t, *J* = 8.1 Hz, 1H), 6.33 (dd, *J* = 8.2, 1.2 Hz, 1H), 4.69 (dd, *J* = 8.7, 4.2 Hz, 1H), 4.48 (s, 3H), 4.45 (dd, *J* = 8.7, 6.2 Hz, 1H), 4.27–4.19 (m, 4H), 3.98–3.88 (m, 4H), 3.60 (s, 3H), 2.61–2.05 (m, 9H), 1.44–1.22 (m, 30H), 0.79 (s, 3H), 0.73 (s, 3H), 0.40 (s, 3H). **HRMS** (ESI+) calcd. for $C_{112}H_{106}N_{16}O_{19}$ $[M+H]^+$ 1979.7893, found 1979.7950.

O_2N-$Q^B\underline{X}Q^BQ^MPQ^B\underline{X}Q^B$-T6f-$Q^BQ^MQ^BQ^B\underline{X}Q^BQ^MQ^B$-Gly-OMe (**5a**) Compound **5a** was synthesized on Fmoc-Gly-HMBA-AM resin (scale: 15 µmol) using SPFS procedures reported in paragraph 5.3. The crude product was purified by RP-HPLC after full-cleavage. The product was obtained as a yellow solid (12 mg, 19%). **^{1}H NMR** (500 MHz, $CDCl_3$, mixture of three conformers in a ratio of 1:0.8:0.2, only the aromatic amide signals are reported.) δ 12.27 (s, 0.2H), 11.89 (s, 0.8H), 11.77 (s, 1H), 11.53 (s, 0.8H), 11.50 (s, 0.2H), 11.49 (s, 0.2H), 11.47 (s, 0.8H), 11.46 (s, 2H), 11.44 (s, 1.8H), 11.42 (s, 1H), 11.40 (s, 1H), 11.32 (s, 0.8H), 11.31 (s, 0.2H), 11.28 (s, 1H), 11.25 (s, 0.8H), 11.24 (s, 1H), 11.21 (s, 0.2H), 11.18 (s, 0.8H), 11.17 (s, 1H), 11.16 (s, 1H), 11.14 (s, 1H), 11.07 (s, 0.2H), 11.01 (s, 0.8H), 10.98 (s, 1H), 10.97 (s, 0.8H), 10.96 (s, 1H), 10.87 (s, 0.2H), 10.86 (s, 0.2H), 10.82 (s, 0.8H), 10.82 (s, 1H), 10.80 (s, 0.8H), 10.79 (s, 1H), 10.77 (s, 0.2H), 10.70 (s, 0.8H), 10.68 (s, 1H), 10.66 (s, 0.8H), 10.65 (s, 1H). **HRMS** (ESI+) calcd. for $C_{232}H_{223}N_{37}O_{38}$ $[2M+2Na]^{2+}$ 2090.3220, found 2090.2690.

O_2N-$Q^BXQ^BQ^MPQ^BXQ^B$-T6f-$Q^BQ^MQ^BQ^BXQ^BQ^MQ^B$-Gly-OMe (**5b**) Compound **5a** (12 mg, 2.9 µmol) was treated with a TFA/DCM 1:1 (3 mL) at r.t. for 2 h. The solvent was removed in vacuo. The residue was washed with diethyl ether, yielding the product as a yellow solid (11.5 mg, quant.). **^{1}H NMR** (500 MHz, CD_2Cl_2) δ 12.09 (s, 1H), 11.53 (s, 1H), 11.52 (s, 1H), 11.41 (s, 2H), 11.22 (s, 2H), 11.05 (s, 1H), 10.99 (s, 2H), 10.90 (s, 1H), 10.79 (s, 2H), 10.58 (s, 1H), 10.31

(s, 1H), 10.27 (s, 1H), 10.14 (s, 1H), 9.76 (s, 1H), 8.71 (t, *J* = 3.3 Hz, 1H), 8.64 (d, *J* = 7.3 Hz, 1H), 8.34 (dd, *J* = 7.9, 1.5 Hz, 1H), 8.29 (d, *J* = 6.9 Hz, 1H), 8.26 (d, *J* = 7.6 Hz, 1H), 8.25–8.20 (m, 3H), 8.19–8.18 (m, 1H), 8.17 (s, 1H), 8.10–8.03 (m, 4H), 7.99 (d, *J* = 8.0 Hz, 1H), 7.98–7.94 (m, 3H), 7.91–7.89 (m, 2H), 7.88–7.83 (m, 3H), 7.81 (dd, *J* = 8.0, 1.3 Hz, 1H), 7.78 (dd, *J* = 7.8, 1.4 Hz, 1H), 7.73 (d, *J* = 8.1 Hz, 1H), 7.63 (s, 1H), 7.61 (d, *J* = 7.3 Hz, 1H), 7.59–7.54 (m, 2H), 7.53–7.41 (m, 7H), 7.41–7.34 (m, 5H), 7.32–7.24 (m, 3H), 7.23 (s, 1H), 7.22–7.16 (m, 6H), 7.14 (s, 1H), 7.11 (d, *J* = 5.3 Hz, 3H), 7.10–7.03 (m, 5H), 6.83 (s, 1H), 6.71 (d, *J* = 7.1 Hz, 1H), 6.59 (t, *J* = 7.8 Hz, 1H), 6.57 (s, 1H), 6.55 (d, *J* = 2.5 Hz, 2H), 6.51 (s, 1H), 6.41 (d, *J* = 2.8 Hz, 2H), 6.14 (d, *J* = 8.3 Hz, 1H), 6.06 (s, 1H), 5.86 (s, 1H), 4.48 (s, 3H), 4.42 (s, 3H), 4.32 (s, 3H), 4.20–4.16 (m, 2H), 4.15–4.05 (m, 2H), 4.03–3.96 (m, 5H), 3.93–3.88 (m, 2H), 3.83 (q, *J* = 7.0, 6.5 Hz, 2H), 3.78 (t, *J* = 7.0 Hz, 1H), 3.72 (t, *J* = 5.8 Hz, 2H), 3.64–3.60 (m, 2H), 3.50 (dd, *J* = 17.6, 6.3 Hz, 1H), 3.09 (s, 3H), 2.89 (dd, *J* = 17.5, 3.1 Hz, 1H), 2.62 (d, *J* = 9.3 Hz, 2H), 2.60–2.21 (m, 10H), 2.19–2.16 (m, 2H), 2.06–1.99 (m, 3H), 1.44–1.11 (m, 60H). **HRMS** (ESI+) calcd. for $C_{220}H_{199}N_{37}O_{38}$ $[2M+2H]^{2+}$ 1984.2461, found 1984.2511.

O_2N-$Q^B\underline{X}Q^BQ^MPQ^B\underline{X}Q^B$-T6r-$Q^BQ^MQ^BQ^B\underline{X}Q^BQ^MQ^B$-Gly-OMe (**6a**) Compound **6a** was synthesized on Fmoc-Gly-HMBA-AM resin (scale: 15 µmol) using SPFS procedures reported in paragraph 5.3. The crude product was purified by RP-HPLC after full-cleavage. The product was obtained as a yellow solid (14 mg, 22%). **^{1}H NMR** (500 MHz, $CDCl_3$) δ 11.58 (s, 1H), 11.44 (s, 2H), 11.30 (d, *J* = 1.6 Hz, 2H), 11.25 (s, 1H), 11.14 (s, 1H), 10.97 (s, 1H), 10.96 (s, 2H), 10.82 (s, 1H), 10.77 (s, 2H), 10.64 (s, 1H), 10.63 (s, 1H), 10.06 (s, 1H), 8.69 (s, 1H), 8.28 (dd, *J* = 8.1, 1.4 Hz, 1H), 8.21 (dd, *J* = 7.4, 1.3 Hz, 1H), 8.17 (dd, *J* = 8.0, 1.3 Hz, 1H), 8.11–8.05 (m, 5H), 8.04 (s, 1H), 8.02 (s, 1H), 7.97–7.94 (m, 2H), 7.92–7.89 (m, 3H), 7.88–7.85 (m, 2H), 7.84 (d, *J* = 6.8 Hz, 1H), 7.81–7.79 (m, 2H), 7.78–7.76 (m, 3H), 7.74 (t, *J* = 1.4 Hz, 2H), 7.73 (t, *J* = 1.5 Hz, 1H), 7.72–7.62 (m, 3H), 7.57–7.54 (m, 2H), 7.49 (dd, *J* = 7.5, 2.3 Hz, 2H), 7.46–7.43 (m, 2H), 7.41–7.35 (m, 3H), 7.30–7.27 (m, 2H), 7.25–7.22 (m, 2H), 7.22–7.20 (m, 2H), 7.19–7.15 (m, 2H), 7.14–7.07 (m, 4H), 7.05–6.99 (m, 5H), 6.96 (s, 1H), 6.96–6.86 (m, 3H), 6.77–6.73 (m, 2H), 6.65 (s, 1H), 6.60 (s, 1H), 6.47 (s, 1H), 6.46 (s, 1H), 6.37 (s, 1H), 6.23 (s, 1H), 6.10 (t, *J* = 7.9 Hz, 1H), 6.05 (s, 1H), 5.94 (s, 1H), 5.85 (s, 1H), 5.79 (s, 1H), 5.62 (dd, *J* = 7.9, 1.2 Hz, 1H), 4.69 (t, *J* = 2.9 Hz, 2H), 4.39–4.36 (m, 1H), 4.18–4.05 (m, 5H), 4.01–3.92 (m, 3H), 3.91–3.80 (m, 7H), 3.76 (s, 3H), 3.70 (t, *J* = 7.9 Hz, 1H), 3.67–3.62 (m, 3H), 3.54 (d, *J* = 7.1 Hz, 6H), 3.26–3.21 (m, 1H), 3.00 (s, 3H), 2.96 (dd, *J* = 17.8, 3.1 Hz, 1H), 2.53–2.12 (m, 10H), 1.69 (s, 9H), 1.52 (s, 9H), 1.43–1.07 (m, 60H), 0.78 (s, 9H). **HRMS** (ESI+) calcd. for $C_{235}H_{223}N_{37}O_{38}$ $[2M+2H]^{2+}$ 2086.3400, found (HR-ESI) 2086.3175.

O_2N-$Q^BXQ^BQ^MPQ^BXQ^B$-T6r-$Q^BQ^MQ^BQ^BXQ^BQ^MQ^B$-Gly-OMe (**6b**) Compound **6a** (14 mg, 3.3 µmol) was treated with a TFA/DCM 1/1 (3 mL) at r.t. for 2 h. The solvent was removed in vacuo. The residue was washed with diethyl ether, yielding the product as a yellow solid (13.4 mg, quant.). **^{1}H NMR** (500 MHz, $CDCl_3$) δ 12.23 (s, 1H), 11.68 (s, 1H), 11.45 (s, 1H), 11.44 (s, 1H), 11.39 (s, 1H), 11.12 (s, 1H), 11.08 (s, 1H), 11.05 (s, 1H), 11.00 (s, 1H), 10.96 (s, 1H), 10.92 (s, 1H), 10.87 (s, 1H), 10.59 (s, 1H), 10.47 (s, 1H), 10.16 (s, 1H), 10.11 (s, 1H), 10.06 (s, 1H), 10.03 (s, 1H), 9.67 (s, 1H), 8.78 (s, 1H), 8.65 (d, *J* = 7.5 Hz, 2H), 8.38 (d, *J* = 7.3 Hz, 1H), 8.31–8.24 (m, 5H), 8.23–8.14 (m, 4H), 8.11–8.07 (m, 5H), 8.06–8.03 (m, 2H), 8.01 (d, *J* = 7.3 Hz, 1H), 7.99–7.95 (m, 3H), 7.92 (d, *J* = 6.5 Hz, 1H), 7.90–7.84 (m, 4H), 7.77 (s, 1H), 7.73 (s, 1H), 7.64–7.58 (m, 6H), 7.53–7.45 (m, 3H), 7.44–7.40 (m, 2H), 7.39–7.34 (m, 3H), 7.34–7.29 (m, 3H), 7.25–7.18 (m, 3H), 7.17–7.12 (m, 3H), 7.12–7.07 (m, 4H), 7.06–7.01 (m, 2H), 6.95–6.88 (m, 2H), 6.79 (s, 1H), 6.76 (s, 1H),

6.68 (d, *J* = 5.4 Hz, 3H), 6.63 (t, *J* = 7.5 Hz, 1H), 6.41 (s, 1H), 6.31 (s, 1H), 6.20 (s, 1H), 6.12 (d, *J* = 7.6 Hz, 1H), 5.95 (d, *J* = 4.3 Hz, 2H), 4.69 (t, *J* = 3.1 Hz, 2H), 4.47 (s, 3H), 4.40–4.37 (m, 2H), 4.32 (s, 3H), 4.27–4.23 (m, 1H), 4.18 (d, *J* = 12.1 Hz, 5H), 4.12 (t, *J* = 6.8 Hz, 1H), 4.04–3.83 (m, 8H), 3.83–3.79 (m, 1H), 3.77–3.71 (m, 2H), 3.67 (dd, *J* = 7.8, 6.0 Hz, 1H), 3.62 (t, *J* = 7.4 Hz, 1H), 3.53 (dd, *J* = 17.4, 6.1 Hz, 1H), 3.50–3.46 (m, 1H), 3.10 (s, 3H), 2.96 (dd, *J* = 17.3, 3.2 Hz, 1H), 2.73–2.16 (m, 10H), 1.43–1.11 (m, 60H). **HRMS** (ESI+) calcd. for $C_{223}H_{199}N_{37}O_{38}$ $[2M+2H]^{2+}$ 2002.2461, found 2202.2471.

Fmoc-$Q^DXQ^DQ^MYQ^DXQ^D$-OH (**25**) Compound **25** was synthesized on SASRIN resin (scale: 100 µmol) using SPFS procedures reported in paragraph 5.3. The crude product was purified by RP-HPLC after full-cleavage. The product was obtained as a yellow solid (196.4 mg, 82%). **^{1}H NMR** (500 MHz, $CDCl_3$) δ 11.54 (s, 1H), 11.29 (s, 1H), 11.27 (s, 1H), 11.11 (s, 1H), 10.92 (s, 1H), 10.65 (s, 1H), 8.22 (dd, *J* = 7.6, 1.3 Hz, 1H), 8.15 (t, *J* = 3.7 Hz, 1H), 8.07 (dd, *J* = 7.5, 1.3 Hz, 1H), 8.03 (s, 1H), 7.98 (dd, *J* = 8.3, 1.3 Hz, 1H), 7.84–7.79 (m, 2H), 7.75–7.72 (m, 2H), 7.69 (dd, *J* = 8.3, 1.4 Hz, 1H), 7.64 (dd, *J* = 8.3, 1.2 Hz, 2H), 7.58 (dd, *J* = 8.3, 1.3 Hz, 1H), 7.51 (t, *J* = 7.9 Hz, 1H), 7.48–7.39 (m, 5H), 7.35–7.31 (m, 2H), 7.25 (t, *J* = 7.9 Hz, 1H), 7.22–7.17 (m, 4H), 7.13–7.11 (m, 2H), 7.08–7.00 (m, 2H), 7.00–6.94 (m, 2H), 6.84 (s, 1H), 6.79 (d, *J* = 7.6 Hz, 1H), 6.63 (d, *J* = 6.1 Hz, 2H), 6.58 (d, *J* = 2.4 Hz, 1H), 6.52–6.50 (m, 1H), 6.46 (t, *J* = 7.5 Hz, 1H), 6.05 (s, 1H), 4.19 (m, 2H), 4.06–3.97 (m, 3H), 3.92–3.77 (m, 10H), 3.73–3.65 (m, 10H), 3.61–3.58 (m, 3H), 3.53–3.50 (m, 2H), 3.49 (s, 3H), 3.48 (s, 3H), 3.41 (s, 3H), 3.39–3.36 (m, 3H), 3.33 (s, 3H), 3.32–3.24 (m, 2H), 3.23 (s, 2H), 3.10 (td, *J* = 6.6, 2.8 Hz, 2H), 3.01 (dt, *J* = 13.1, 6.6 Hz, 1H), 2.92 (dt, *J* = 12.9, 6.7 Hz, 1H), 2.23 (m, 1H), 1.62 (s, 9H), 1.53 (s, 9H), 1.32–1.28 (m, 2H), 0.19 (s, 9H). **HRMS** (ESI+) calcd. for $C_{126}H_{130}N_{16}O_{23}S_4Si$ $[M+H]^+$ 2391.8220, found 2391.7467.

Fmoc-YQ^DXQ^D-OH (**26**) Compound **26** was synthesized on SASRIN resin (scale: 25 µmol) using SPFS procedure reported in paragraph 5.3. The crude product was purified by RP-HPLC after full-cleavage. The product was obtained as a yellow solid (22 mg, 66%). **^{1}H NMR** (500 MHz, $CDCl_3$) δ 12.24 (s, 1H), 11.75 (s, 1H), 11.70 (s, 1H), 8.99 (s, 1H), 8.45 (d, *J* = 7.1 Hz, 1H), 8.25 (s, 1H), 8.00 (d, *J* = 7.8 Hz, 2H), 7.91 (d, *J* = 8.1 Hz, 1H), 7.77 (d, *J* = 8.1 Hz, 1H), 7.73 (s, 1H), 7.70–7.60 (m, 5H), 7.36–7.29 (m, 4H), 7.24 (s, 2H), 7.12 (s, 1H), 6.98 (s, 2H), 6.57 (s, 1H), 5.40 (s, 2H), 4.25 (t, *J* = 7.9 Hz, 2H), 4.10 (s, 2H), 3.90 (t, *J* = 6.2 Hz, 5H), 3.77–3.66 (m, 4H), 3.65–3.55 (m, 5H), 3.44 (s, 4H), 3.31 (d, *J* = 10.0 Hz, 6H), 1.73 (s, 9H), 1.29–1.25 (m, 2H), 0.18 (s, 9H). **HRMS** (ESI+) calcd. for $C_{71}H_{76}N_8O_{13}S_2Si$ $[M+H]^+$ 1341.4815, found 1341.4820.

Fmoc-YQ^DXQ^D-T6r-$Q^DXQ^DQ^M$-OH (**27**) Compound **27** was synthesized on SASRIN resin (scale: 50 µmol) using SPFS procedure reported in paragraph 5.3. The crude product was purified by RP-HPLC after full-cleavage. The product was obtained as a yellow solid (72.8 mg, 52%). **^{1}H NMR** (500 MHz, $CDCl_3$) δ 12.02 (s, 1H), 12.01 (s, 1H), 11.99 (s, 1H), 11.93 (s, 2H), 11.85 (s, 1H), 11.70 (s, 1H), 11.24 (s, 1H), 10.39 (s, 1H), 9.07 (s, 1H), 8.67 (d, *J* = 7.5 Hz, 1H), 8.62 (d, *J* = 7.4 Hz, 1H), 8.56 (d, *J* = 7.5 Hz, 1H), 8.49 (d, *J* = 7.5 Hz, 1H), 8.29 (d, *J* = 7.8 Hz, 1H), 8.23–8.20 (m, 2H), 8.14 (d, *J* = 7.4 Hz, 1H), 8.11–8.07 (m, 2H), 8.06–8.05 (m, 2H), 8.04–8.03 (m, 2H), 8.02 (s, 1H), 7.99 (d, *J* = 7.9 Hz, 1H), 7.94 (d, *J* = 8.3 Hz, 1H), 7.91–7.88 (m, 1H), 7.87 (s, 1H), 7.86 (d, *J* = 8.3 Hz, 1H), 7.81 (t, *J* = 7.8 Hz, 1H), 7.75 (d, *J* = 8.0 Hz, 1H), 7.73–7.68 (m, 3H), 7.56 (d, *J* = 6.0 Hz, 2H), 7.55–7.53 (m, 2H), 7.52–7.45 (m, 4H), 7.36–7.28 (m, 4H), 7.23–7.10 (m, 4H), 7.01 (s, 1H), 6.99 (d, *J* = 7.5 Hz, 1H), 6.86 (t, *J* = 7.3 Hz, 1H), 6.74 (t, *J* = 7.4 Hz, 1H), 6.71 (s, 1H), 6.46 (t, *J* = 8.0 Hz, 1H),

6.39 (s, 1H), 6.26 (d, *J* = 8.1 Hz, 1H), 5.22–5.19 (m, 1H), 4.60–4.47 (m, 2H), 4.28–4.19 (m, 3H), 4.11 (t, *J* = 6.6 Hz, 2H), 4.01 (s, 3H), 4.00–3.93 (m, 5H), 3.91–3.78 (m, 13H), 3.71–3.66 (m, 14H), 3.61–3.58 (m, 2H), 3.51–3.48 (m, 4H), 3.48 (s, 3H), 3.47 (s, 3H), 3.44 (s, 3H), 3.43–3.38 (m, 3H), 1.46 (s, 9H), 0.91 (s, 9H), 0.88 (t, *J* = 6.9 Hz, 2H), 0.14 (s, 9H). **HRMS** (ESI+) calcd. for $C_{150}H_{150}N_{20}O_{26}S_4Si$ [2M+2H]$^{2+}$ 1402.4914, found 1402.4937.

Fmoc-$\mathrm{Q^{D}\underline{X}Q^{D}Q^{M}}$-OH (**28**) Compound **28** was synthesized on SASRIN resin (scale: 25 µmol) using SPFS procedure reported in paragraph 5.3. The crude product was purified by RP-HPLC after full-cleavage. The product was obtained as a yellow solid (26.5 mg, 82%). **^{1}H NMR** (500 MHz, $CDCl_3$) δ 12.27 (s, 1H), 11.85 (s, 1H), 11.29 (s, 1H), 9.12 (d, *J* = 7.6 Hz, 1H), 8.58 (d, *J* = 8.1 Hz, 2H), 8.05 (d, *J* = 7.5 Hz, 1H), 7.99 (d, *J* = 7.4 Hz, 1H), 7.94–7.90 (m, 2H), 7.82–7.76 (m, 4H), 7.72 (t, *J* = 8.0 Hz, 1H), 7.63 (d, *J* = 7.4 Hz, 1H), 7.59 (d, *J* = 7.4 Hz, 1H), 7.57–7.54 (m, 1H), 7.44 (d, *J* = 7.1 Hz, 1H), 7.36–7.29 (m, 2H), 7.24–7.19 (m, 2H), 7.14 (t, *J* = 7.2 Hz, 1H), 6.86 (t, *J* = 7.2 Hz, 2H), 6.80 (s, 1H), 6.76 (d, *J* = 7.2 Hz, 1H), 4.18–4.14 (m, 2H), 4.08–4.04 (m, 5H), 3.90 (q, *J* = 6.6 Hz, 2H), 3.83–3.80 (m, 2H), 3.77–3.75 (m, 2H), 3.69–3.63 (m, 5H), 3.61 (t, *J* = 6.6 Hz, 2H), 3.46 (s, 3H), 3.45 (s, 3H), 3.34–3.26 (m, 2H), 1.74 (s, 9H). **HRMS** (ESI+) calcd. for $C_{70}H_{66}N_8O_{13}S_2$ [M+Na]$^+$ 1313.4083, found 1313.4009.

C*-$\mathrm{Q^{D}\underline{X}Q^{D}Q^{M}\underline{Y}Q^{D}\underline{X}Q^{D}}$-T6f-$\mathrm{Q^{D}\underline{X}Q^{D}Q^{M}\underline{Y}Q^{D}\underline{X}Q^{D}}$-T6f-$\mathrm{Q^{D}\underline{X}Q^{D}Q^{M}\underline{Y}Q^{D}\underline{X}Q^{D}}$-Gly-OMe (**7a**) Compound **7a** was synthesized on Fmoc-Gly-HMBA-AM resin (100 mg, 25 µmol) using SPFS procedure reported in paragraph 5.3. Compounds **25**, **22**, **25**, **22**, **25** and (1*S*)-camphanic chloride were coupled accordingly. The coupling of compound **25** was carried out according to the procedure reported in 5.3.4. The coupling of compound **22** and (1*S*)-camphanic chloride was carried out according to the procedures reported in 5.3.3. The sequence was purified by GPC after full-cleavage. The product was obtained as a yellow solid (18.7 mg, 10%). **^{1}H NMR** (500 MHz, $CDCl_3$, see section 7) showed four sets of signals with a ratio of 1:0.9:0.85:0.8 and is therefore not reported due its complexity. **HRMS** (ESI+) calcd. for $C_{388}H_{413}N_{57}O_{71}S_{12}Si_3$ [3M+3H]$^{3+}$ 2492.2211, found 2942.1987.

C*-$\mathrm{Q^{D}XQ^{D}Q^{M}YQ^{D}XQ^{D}}$-T6f-$\mathrm{Q^{D}XQ^{D}Q^{M}YQ^{D}XQ^{D}}$-T6f-$\mathrm{Q^{D}XQ^{D}Q^{M}YQ^{D}XQ^{D}}$-Gly-OMe (**7b**) Compound **7a** (15 mg, 2.0 µmol) was treated with a TFA/DCM 1/1 (3 mL) at r.t. overnight. The solvent was removed in vacuo. The residue was precipitated from diethyl ether to afford the product as a yellow solid (13.7 mg, quant.). **^{1}H NMR** (500 MHz, pyridine-d_5, see section 7) showed four sets of signals with a ratio of around 1:0.9:0.85:0.8 and is therefore not reported due to its complexity. **HRMS** (ESI+) calcd. for $C_{349}H_{329}N_{57}O_{71}S_{12}$ [3M+3H]$^{3+}$ 2280.0251, found 2280.0125.

C*-$\mathrm{Q^{D}\underline{X}Q^{D}Q^{M}\underline{Y}Q^{D}\underline{X}Q^{D}}$-T6r-$\mathrm{Q^{D}\underline{X}Q^{D}Q^{M}\underline{Y}Q^{D}\underline{X}Q^{D}}$-T6r-$\mathrm{Q^{D}\underline{X}Q^{D}Q^{M}\underline{Y}Q^{D}\underline{X}Q^{D}}$-Gly-OMe (**8a**) Compound **8a** was synthesized on Fmoc-Gly-HMBA-AM resin (100 mg, 25 µmol) using SPFS procedure reported in paragraph 5.3. Compounds **26**, **27**, **27**, **28** and (1*S*)-camphanic chloride were coupled accordingly. The sequence was cleaved and purified by GPC. The product was obtained as a yellow solid (17 mg, 9%).**^{1}H NMR** (500 MHz, $CDCl_3$) δ 11.50 (s, 1H), 11.49 (s, 1H), 11.40 (s, 1H), 11.34 (s, 1H), 11.33 (s, 1H), 11.28 (s, 1H), 11.27 (s, 1H), 11.23 (s, 1H), 11.21 (s, 1H), 11.19 (s, 2H), 11.05 (s, 1H), 11.04 (s, 1H), 10.94 (s, 1H), 10.82 (s, 1H), 10.77 (s, 1H), 10.68 (s, 1H), 10.64 (s, 1H), 10.57 (s, 2H), 10.55 (s, 1H), 10.53 (s, 1H), 9.84 (s, 1H), 9.82 (s, 1H), 9.33 (s, 1H), 9.33 (s, 1H), 8.66 (s, 1H), 8.63 (s, 1H), 8.20 (d, *J* = 7.1 Hz, 1H), 8.15 (s, 1H), 8.12–8.09 (m, 2H), 8.07 (s, 2H), 8.05–7.98 (m, 3H), 7.97–7.91 (m, 5H), 7.90–7.83 (m, 3H), 7.78 (s, 1H), 7.77 (s, 1H), 7.75–7.68 (m, 6H), 7.67–7.61 (m, 8H), 7.60 (s, 1H), 7.59–7.56 (m, 5H), 7.55–7.52 (m, 2H), 7.51–7.46 (m, 7H),

7.46–7.40 (m, 3H), 7.38–7.33 (m, 4H), 7.32–7.31 (m, 1H), 7.31–7.30 (m, 1H), 7.28–7.21 (m, 3H), 7.21–7.15 (m, 5H), 7.15–7.11 (m, 5H), 7.10–7.09 (m, 4H), 7.08–7.05 (m, 2H), 7.04–7.01 (m, 2H), 6.99 (s, 1H), 6.96–6.91 (m, 3H), 6.86–6.80 (m, 4H), 6.70–6.63 (m, 4H), 6.55 (s, 1H), 6.46 (d, *J* = 10.5 Hz, 2H), 6.36–6.30 (m, 2H), 6.25 (s, 1H), 6.23 (s, 2H), 6.21 (s, 1H), 6.17–6.13 (m, 3H), 6.11–6.08 (m, 2H), 5.99 (s, 1H), 5.95 (s, 1H), 5.85 (s, 1H), 5.80 (d, *J* = 7.8 Hz, 1H), 5.74 (s, 1H), 4.28–4.25 (m, 1H), 4.24–4.20 (m, 4H), 4.15 (s, 4H), 4.07–3.85 (m, 25H), 3.84–3.68 (m, 28H), 3.67–3.62 (m, 42H), 3.61 (s, 3H), 3.60 (s, 3H), 3.57 (s, 3H), 3.57 (s, 3H), 3.55 (s, 3H), 3.50 (s, 3H), 3.49 (s, 3H), 3.45 (s, 3H), 3.44 (s, 3H), 3.43 (s, 3H), 3.39 (s, 3H), 3.39 (s, 3H), 3.37–3.29 (m, 6H), 3.24 (d, *J* = 27.4 Hz, 4H), 3.11 (s, 3H), 3.04 (d, *J* = 33.1 Hz, 2H), 2.99 (s, 3H), 2.79 (dd, *J* = 17.4, 2.7 Hz, 1H), 2.69–2.61 (m, 2H), 2.34–2.30 (m, 4H), 2.24–2.19 (m, 4H), 1.49 (s, 9H), 1.45 (s, 9H), 1.26 (d, *J* = 8.5 Hz, 35H), 1.05 (s, 9H), 0.64 (s, 9H), 0.48 (s, 9H), 0.18 (s, 9H), 0.07 (s, 9H), 0.06 (s, 9H). **HRMS** (ESI+) calcd. for $C_{394}H_{413}N_{57}O_{71}S_{12}Si_3$ $[3M+3Na]^{3+}$ 2538.2046, found 2538.2031.

C*-$Q^DXQ^DQ^MYQ^DXQ^D$-T6r-$Q^DXQ^DQ^MYQ^DXQ^D$-T6r-$Q^DXQ^DQ^MYQ^DXQ^D$-Gly-OMe (**8b**) Compound **8a** (15 mg, 2.0 µmol) was treated with a TFA/DCM 1:1 (3 mL) at r.t. overnight. The solvent was removed in vacuo. The residue was precipitated from diethyl ether to afford the desired product as a yellow solid (13.7 mg, quant.). ^{1}**H NMR** (500 MHz, CD_2Cl_2, only the aromatic amide signals and H-bonded hydroxy group signals are reported.). δ 12.12 (s, 1H), 11.60 (s, 2H), 11.46 (s, 1H), 11.33 (s, 1H), 11.17 (s, 1H), 11.06 (s, 1H), 10.96 (s, 1H), 10.95 (s, 1H), 10.93 (s, 1H), 10.81 (s, 1H), 10.75 (s, 2H), 10.56 (s, 1H), 10.53 (s, 1H), 10.51 (s, 1H), 10.42 (s, 2H), 10.40 (s, 1H), 10.30 (s, 1H), 10.26 (s, 1H), 10.23 (s, 2H), 10.14 (s, 1H), 10.11 (s, 2H), 10.00 (s, 1H), 9.84 (s, 1H), 9.74 (s, 1H), 9.39 (s, 1H), 9.31 (s, 1H), 9.25 (s, 1H), 9.00 (s, 1H), 8.80 (s, 1H). **HRMS** (ESI+) calcd. for $C_{355}H_{329}N_{57}O_{71}S_{12}$ $[3M+3H]^{3+}$ 2304.0251, found 2034.0625.

6 References

1. S. De, B. Chi, T. Granier, T. Qi, V. Maurizot and I. Huc, *Nat. Chem.*, 2018, **10**, 51-57.
2. D. Mazzier, S. De, B. Wicher, V. Maurizot and I. Huc, *Angew. Chem. Int. Ed.*, 2020, **59**, 1606-1610.
3. F. S. Menke, B. Wicher, V. Maurizot and I. Huc, *Angew. Chem. Int. Ed.*, 2023, **62**, e202217325.
4. Rigaku-Oxford-Diffraction, *CrysAlisPro Software system, version 1.171.43.130a*, 2024, Rigaku Corporation, Wroclaw, Poland.
5. Rigaku-Oxford-Diffraction, *AutoChem 6.0 software system in conjunction with Olex2 1.5-ac6*, 2024, Rigaku Corporation, Wroclaw, Poland.
6. G. M. Sheldrick, *Acta Crystallogr. Sect. A: Found. Crystallogr.*, 2008, **64**, 112-122.
7. O. V. Dolomanov, L. J. Bourhis, R. J. Gildea, J. A. Howard and H. Puschmann, *J. Appl. Crystallogr.*, 2009, **42**, 339-341.
8. V. Corvaglia, F. Sanchez, F. S. Menke, C. Douat and I. Huc, *Chem. Eur. J.*, 2023, **29**, e202300898.
9. J. Buratto, C. Colombo, M. Stupfel, S. J. Dawson, C. Dolain, B. Langlois d'Estaintot, L. Fischer, T. Granier, M. Laguerre, B. Gallois and I. Huc, *Angew. Chem. Int. Ed.*, 2014, **53**, 883-887.
10. B. Baptiste, C. Douat-Casassus, K. Laxmi-Reddy, F. Godde and I. Huc, *J. Org. Chem.*, 2010, **75**, 7175-7185.
11. M. Vallade, P. Sai Reddy, L. Fischer and I. Huc, *Eur. J. Org. Chem.*, 2018, **2018**, 5489-5498.
12. B. Teng, J. Atcher, L. Allmendinger, C. Douat, Y. Ferrand and I. Huc, *Org. Biomol. Chem.*, 2023, **21**, 3525-3530.
13. O. Al Musaimi, A. Basso, B. G. de la Torre and F. Albericio, *ACS Comb. Sci.*, 2019, **21**, 717-721.
14. J. Hansen, F. Diness and M. Meldal, *Org. Biomol. Chem.*, 2016, **14**, 3238-3245.
15. S. Wang, B. Wicher, C. Douat, V. Maurizot and I. Huc, *Angew. Chem. Int. Ed.*, 2024, e202405091.

7 NMR spectra of new compounds

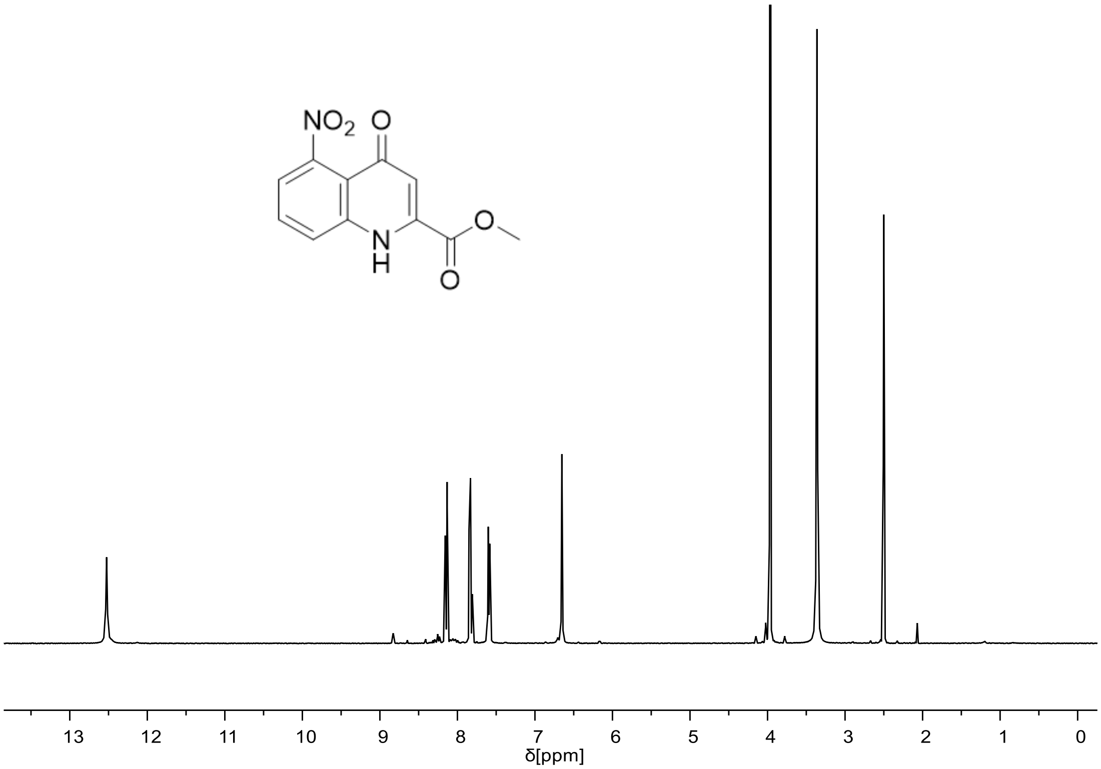

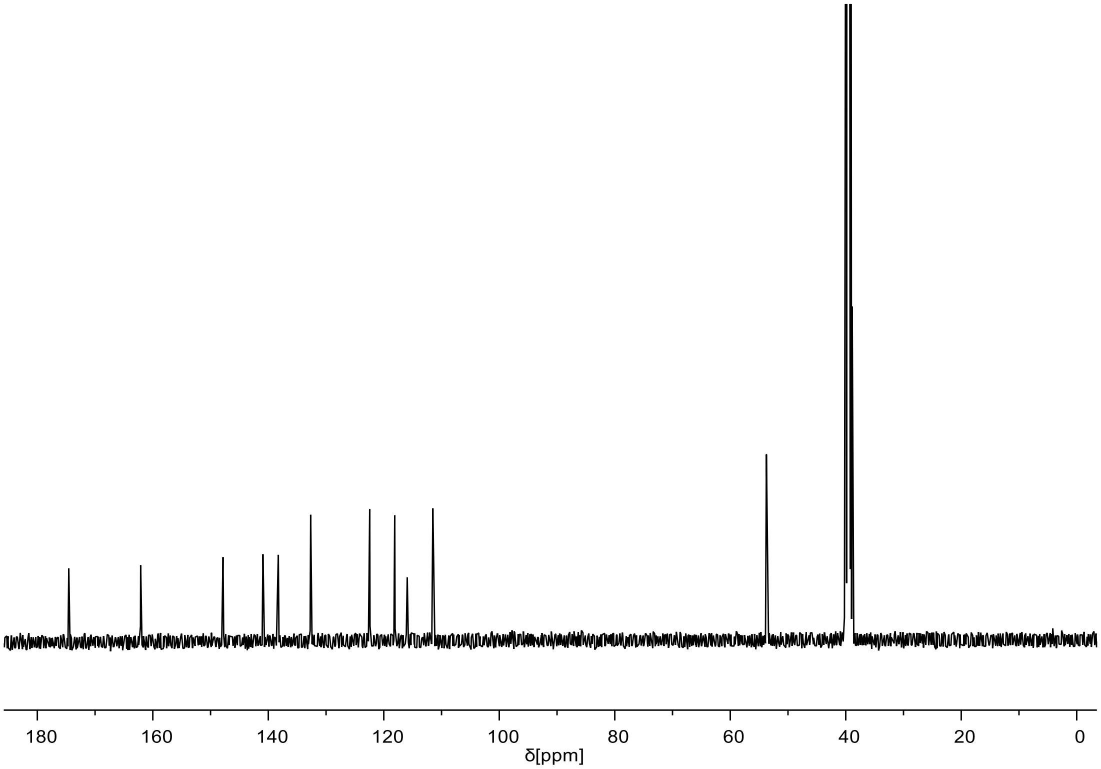

^{1}H NMR spectrum (400 MHz, DMSO-d_6) and ^{13}C NMR (101 MHz, DMSO-d_6) of compound **9**.

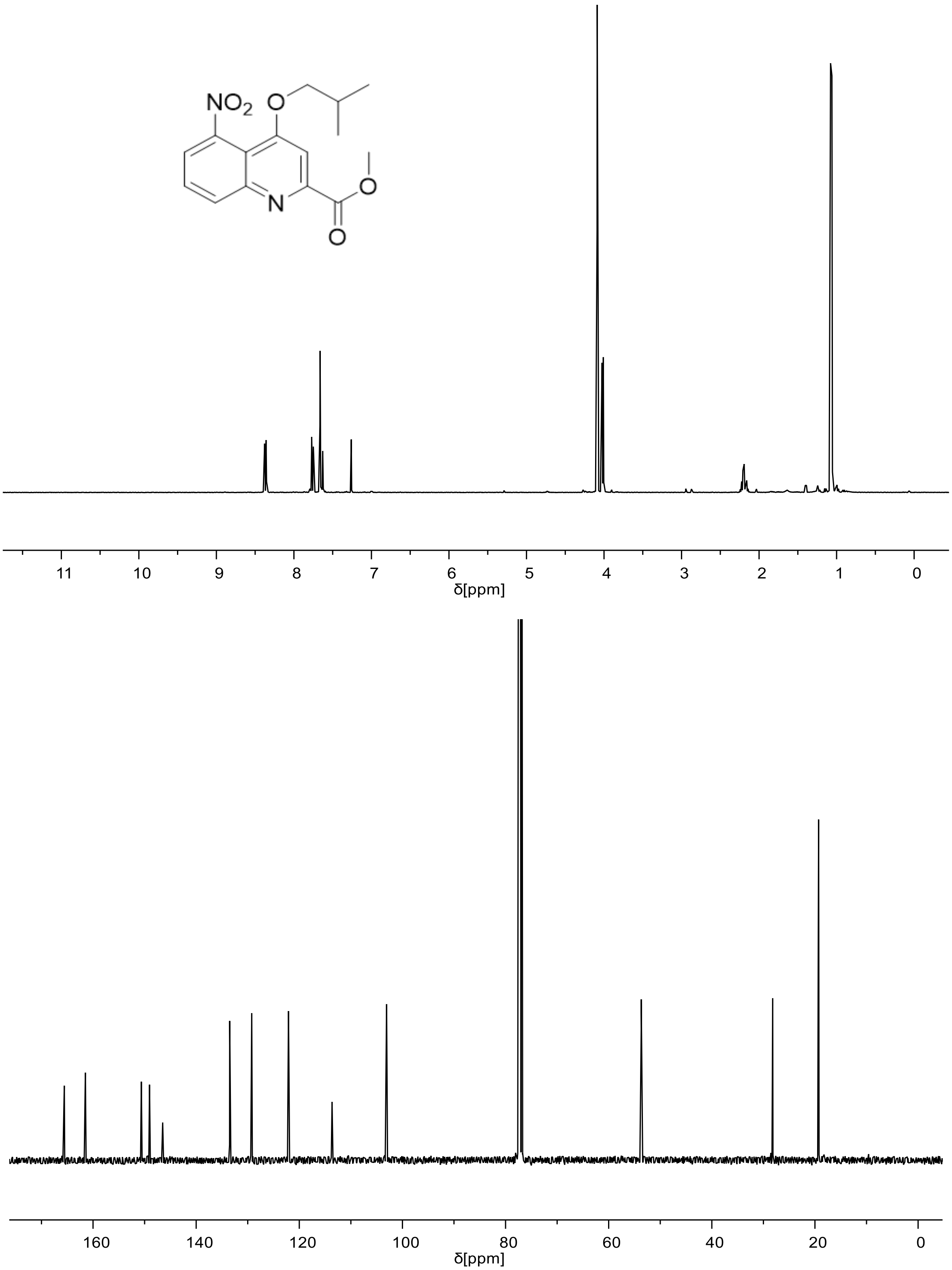

^{1}H NMR spectrum (400 MHz, $CDCl_3$) and ^{13}C NMR (101 MHz, $CDCl_3$) of compound **10**.

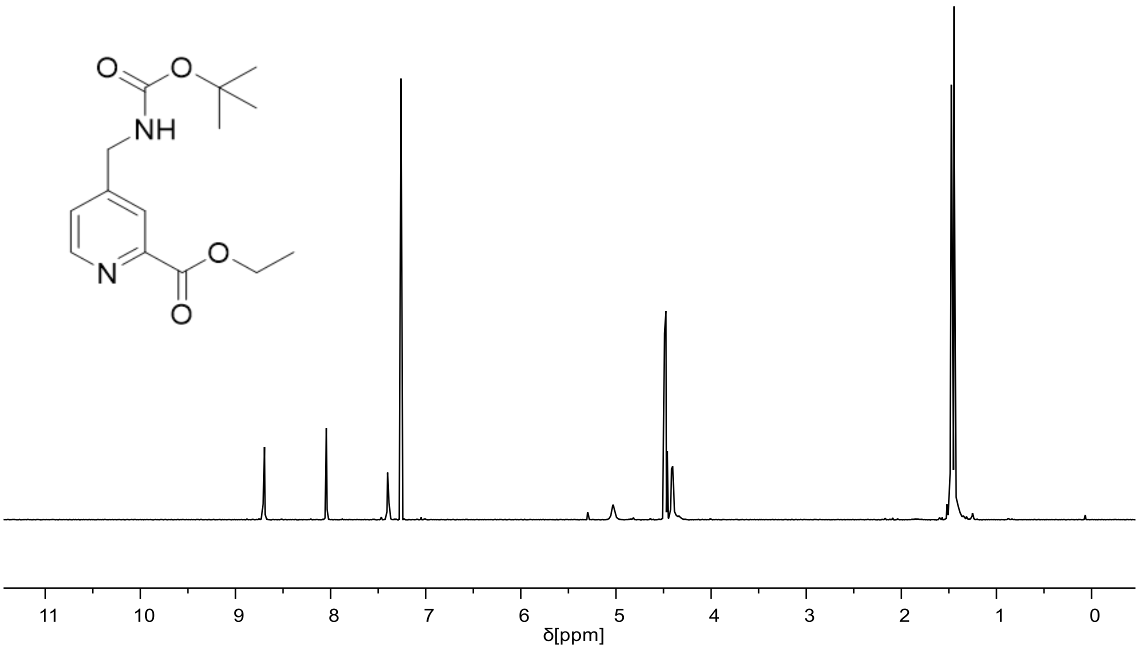

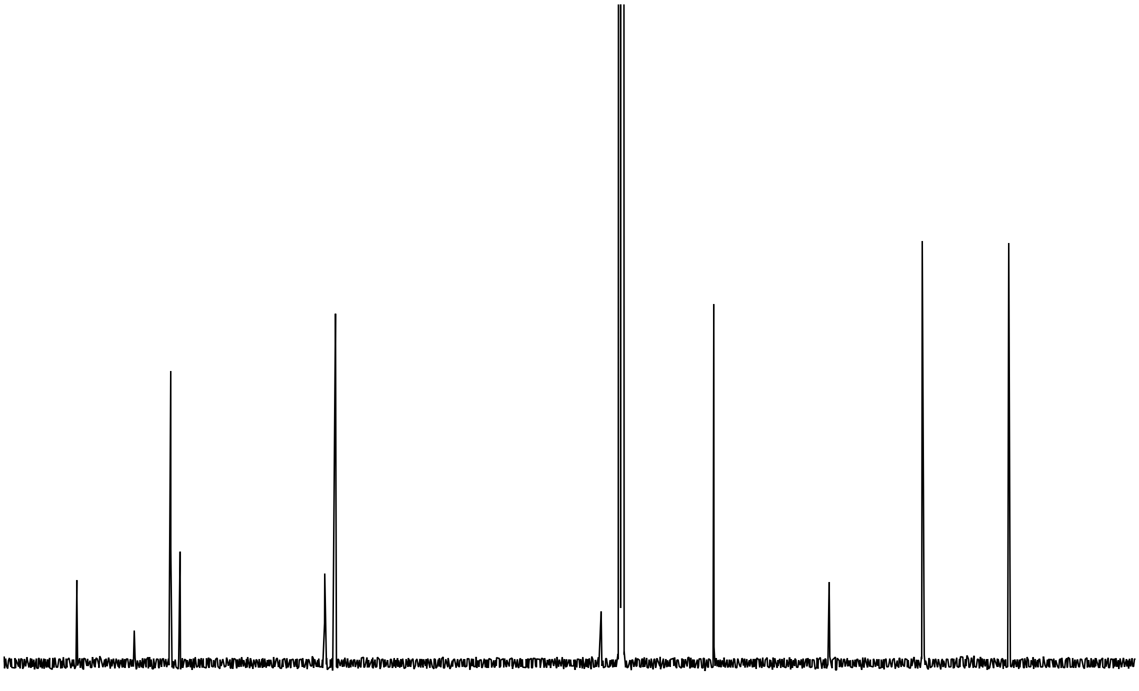

^{1}H NMR spectrum (500 MHz, $CDCl_3$) and ^{13}C NMR (126 MHz, $CDCl_3$) of compound **12**.

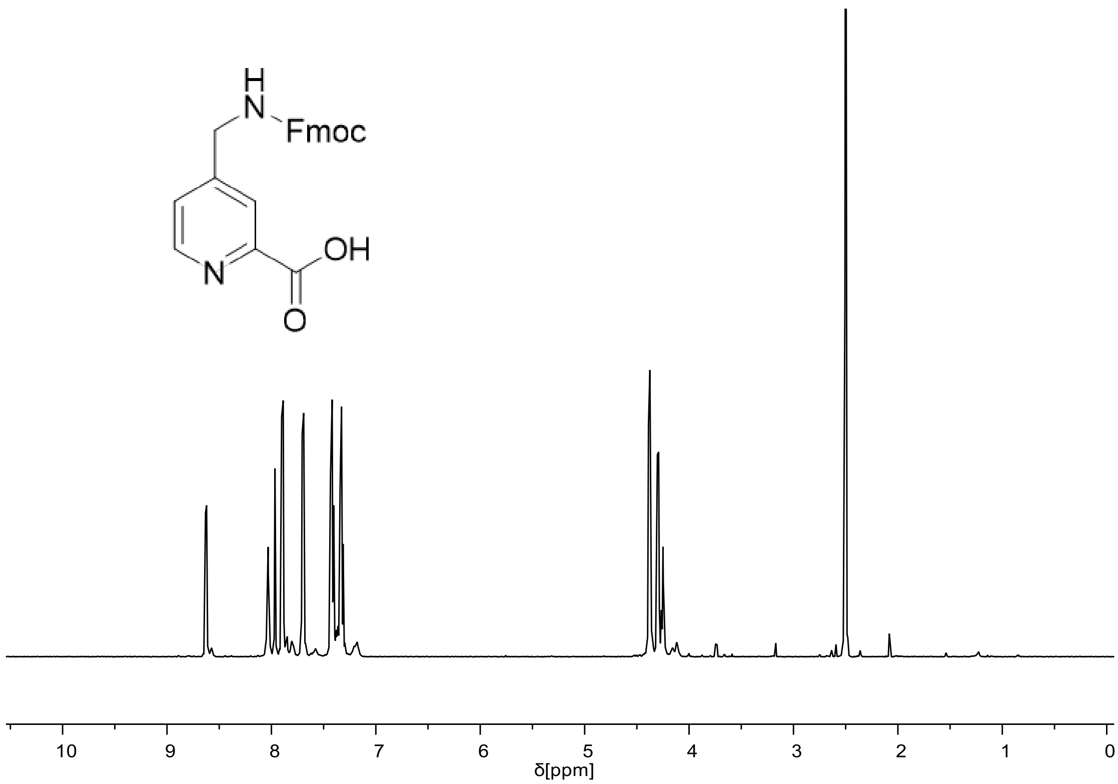

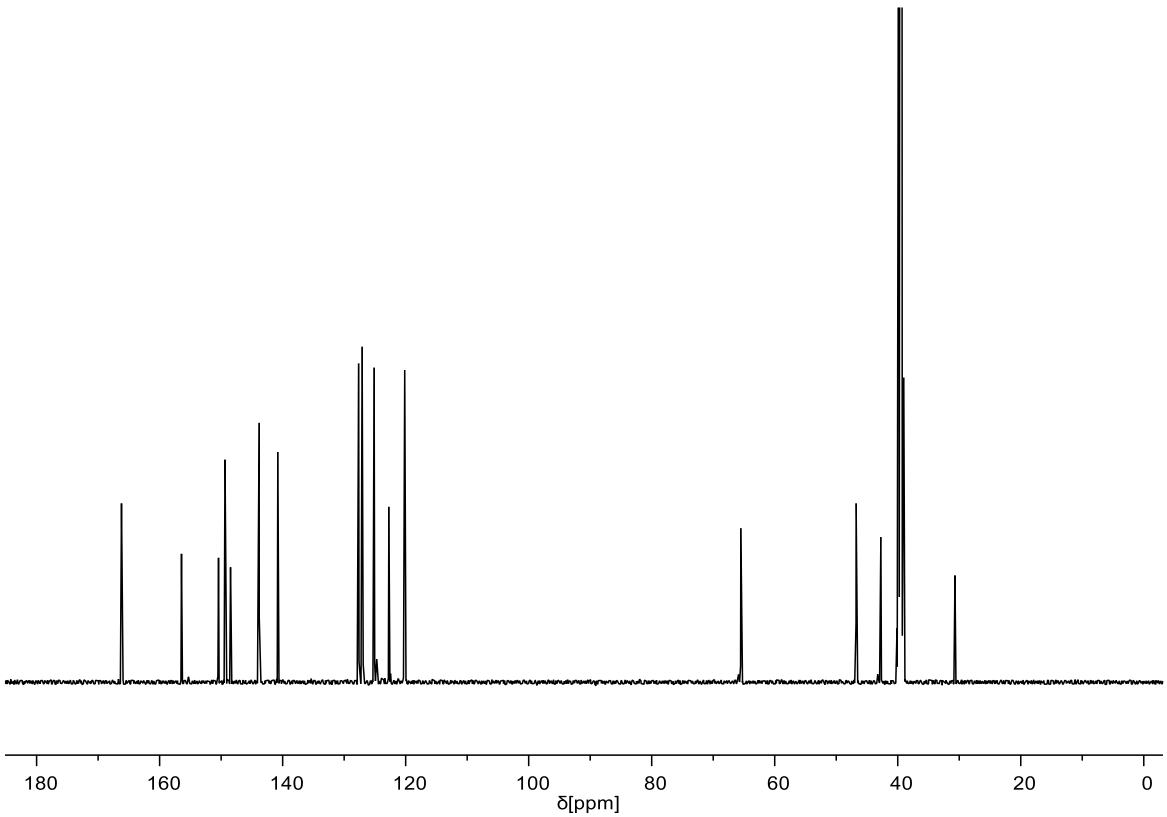

^{1}H NMR spectrum (500 MHz, DMSO-d_6) and ^{13}C NMR (126 MHz, DMSO-d_6) of compound **14**.

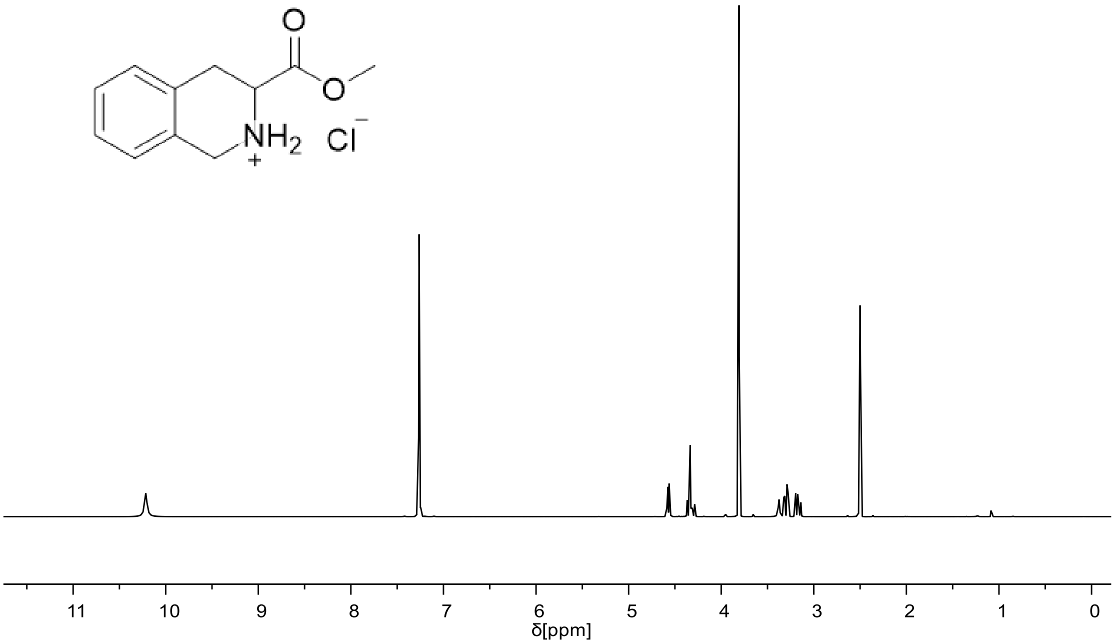

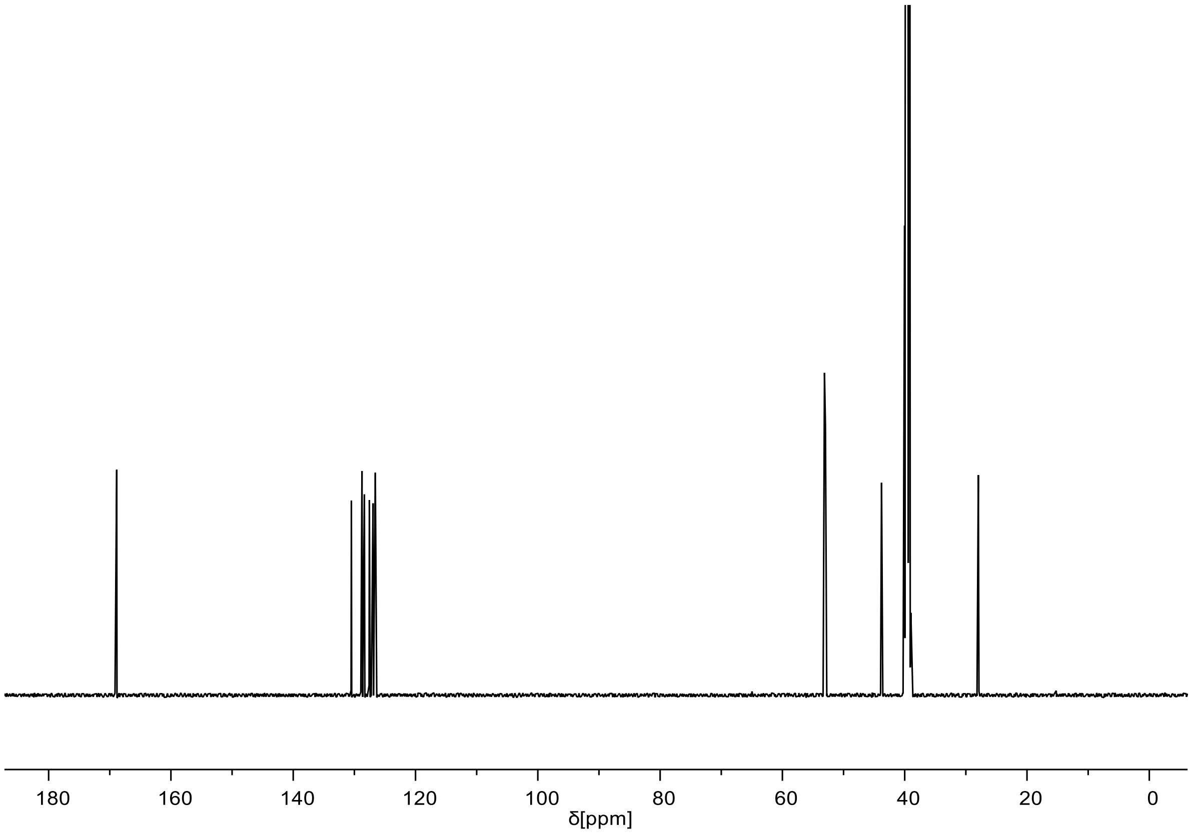

^{1}H NMR spectrum (500 MHz, DMSO-d_6) and ^{13}C NMR (126 MHz, DMSO-d_6) of compound **15**.

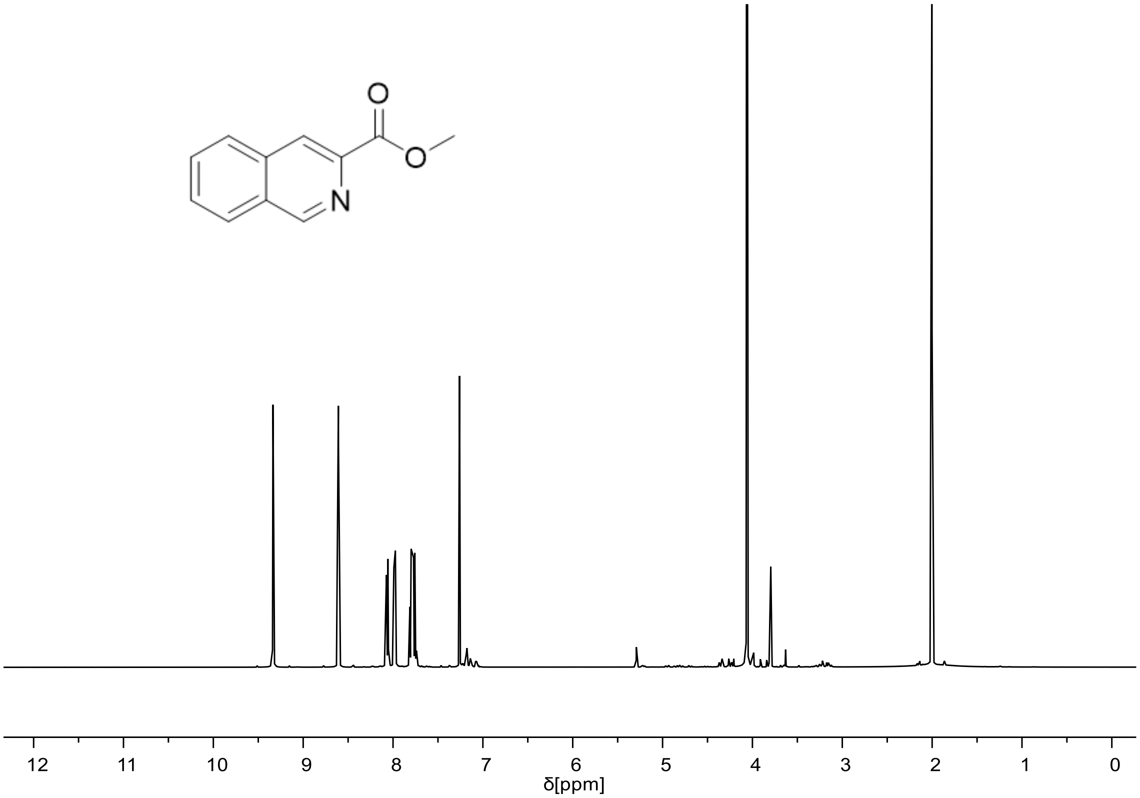

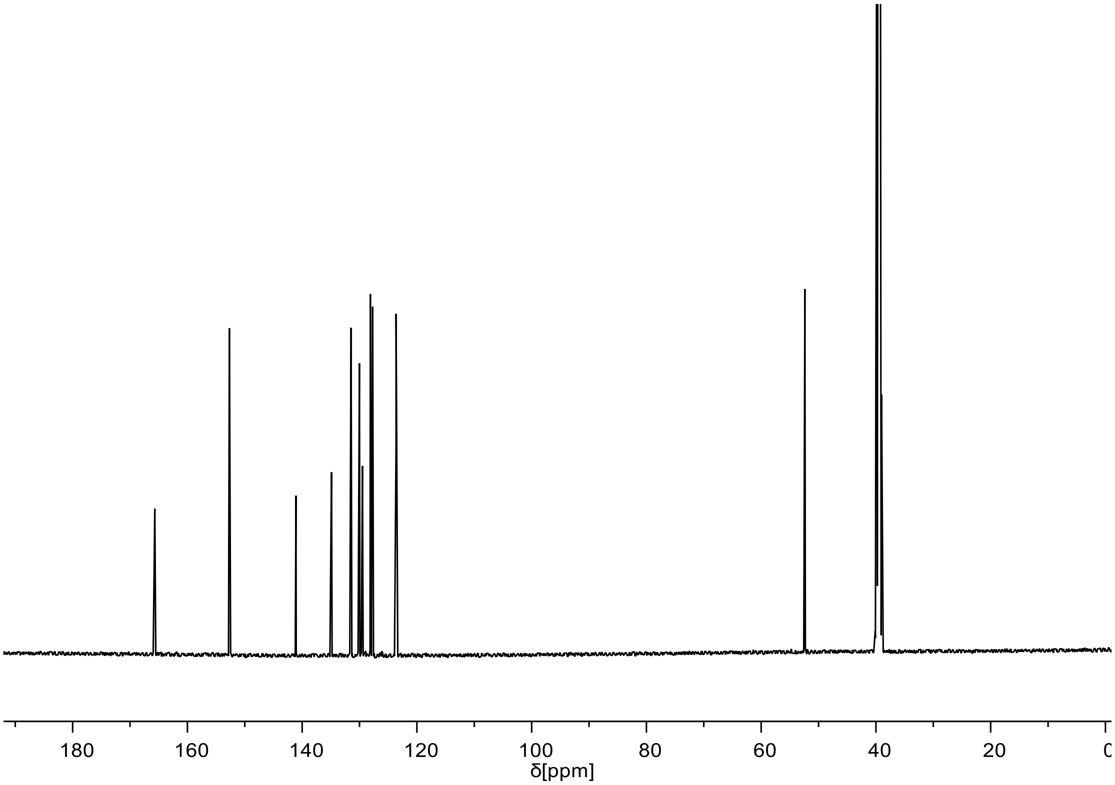

^{1}H NMR spectrum (500 MHz, $CDCl_3$) and ^{13}C NMR (126 MHz, DMSO-d_6) of compound **16**.

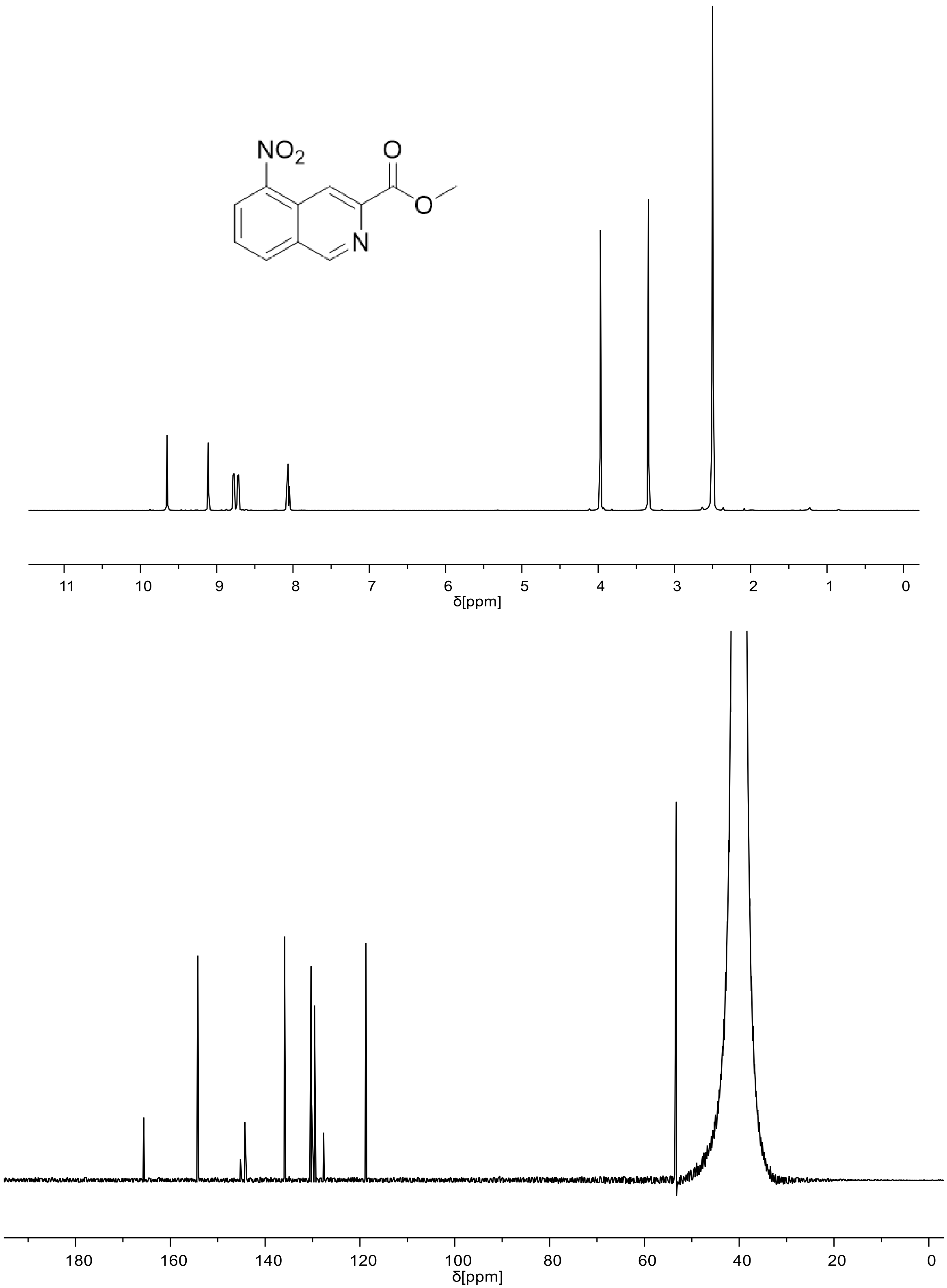

^{1}H NMR spectrum (500 MHz, DMSO-d_6) and ^{13}C NMR (126 MHz, DMSO-d_6) of compound **17**.

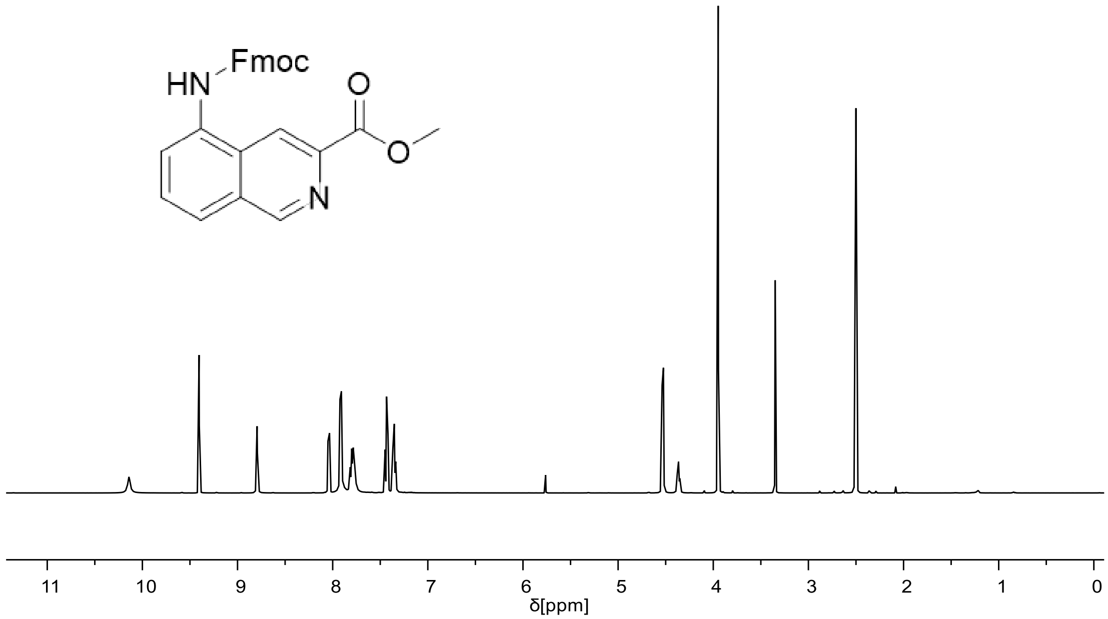

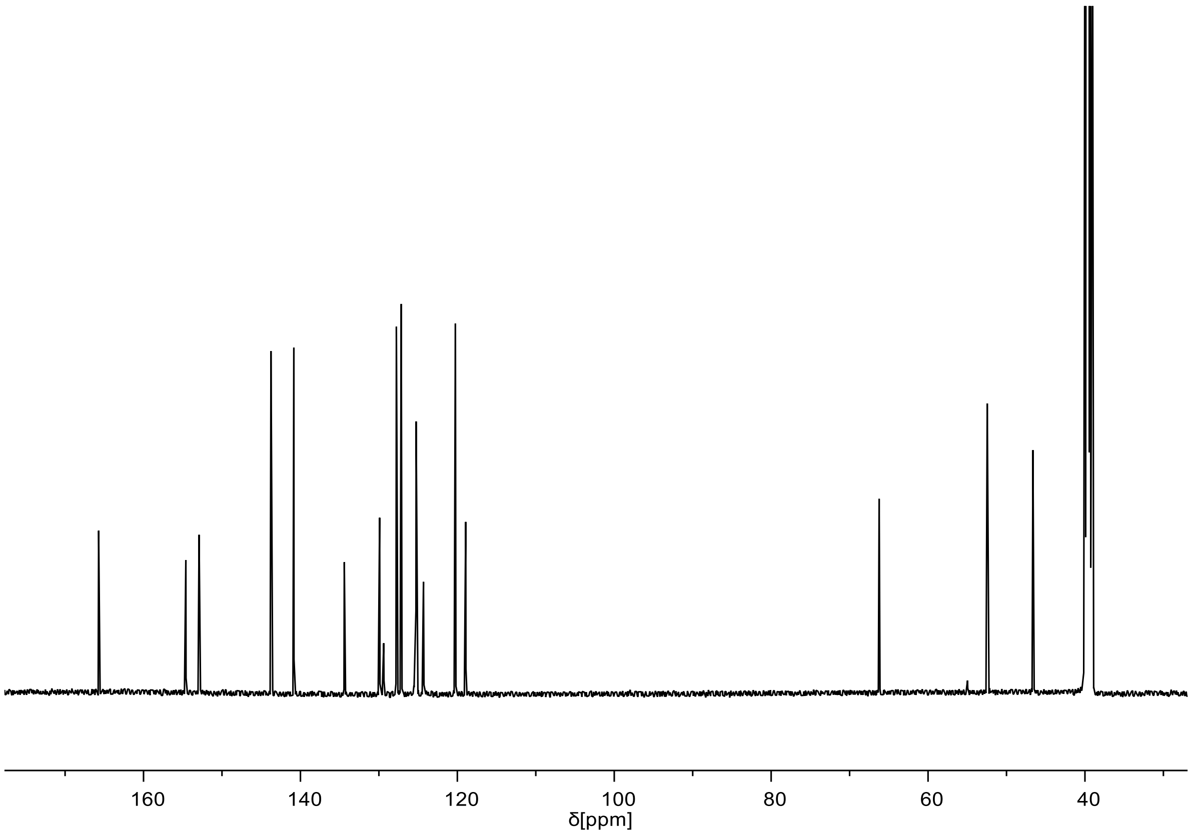

^{1}H NMR spectrum (500 MHz, DMSO-d_6) and ^{13}C NMR (126 MHz, DMSO-d_6) of compound **19**.

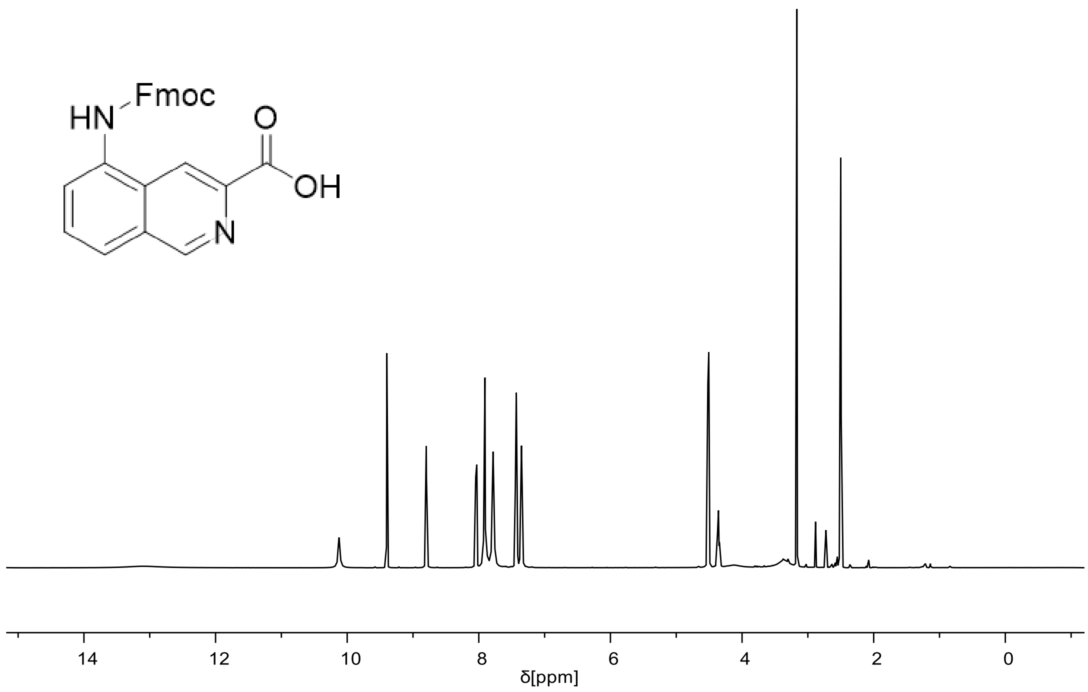

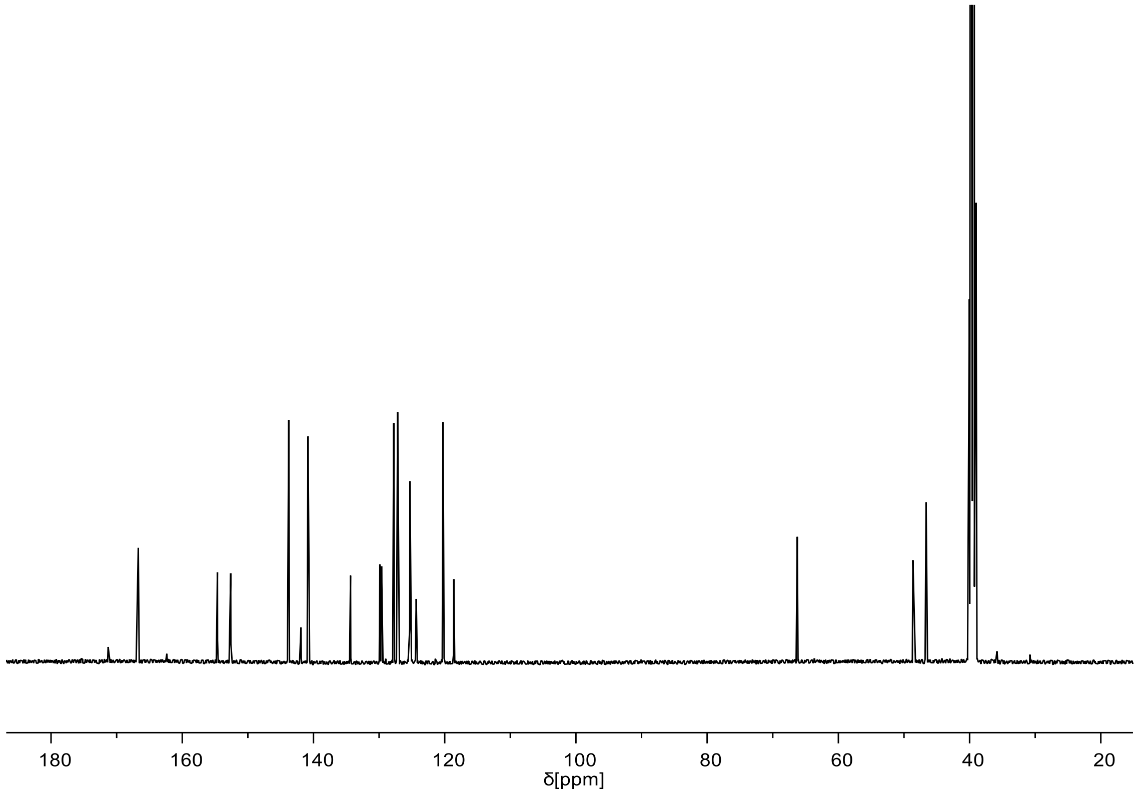

^{1}H NMR spectrum (500 MHz, DMSO-*d*6) and ^{13}C NMR (126 MHz, DMSO-*d*6) of compound **20**.

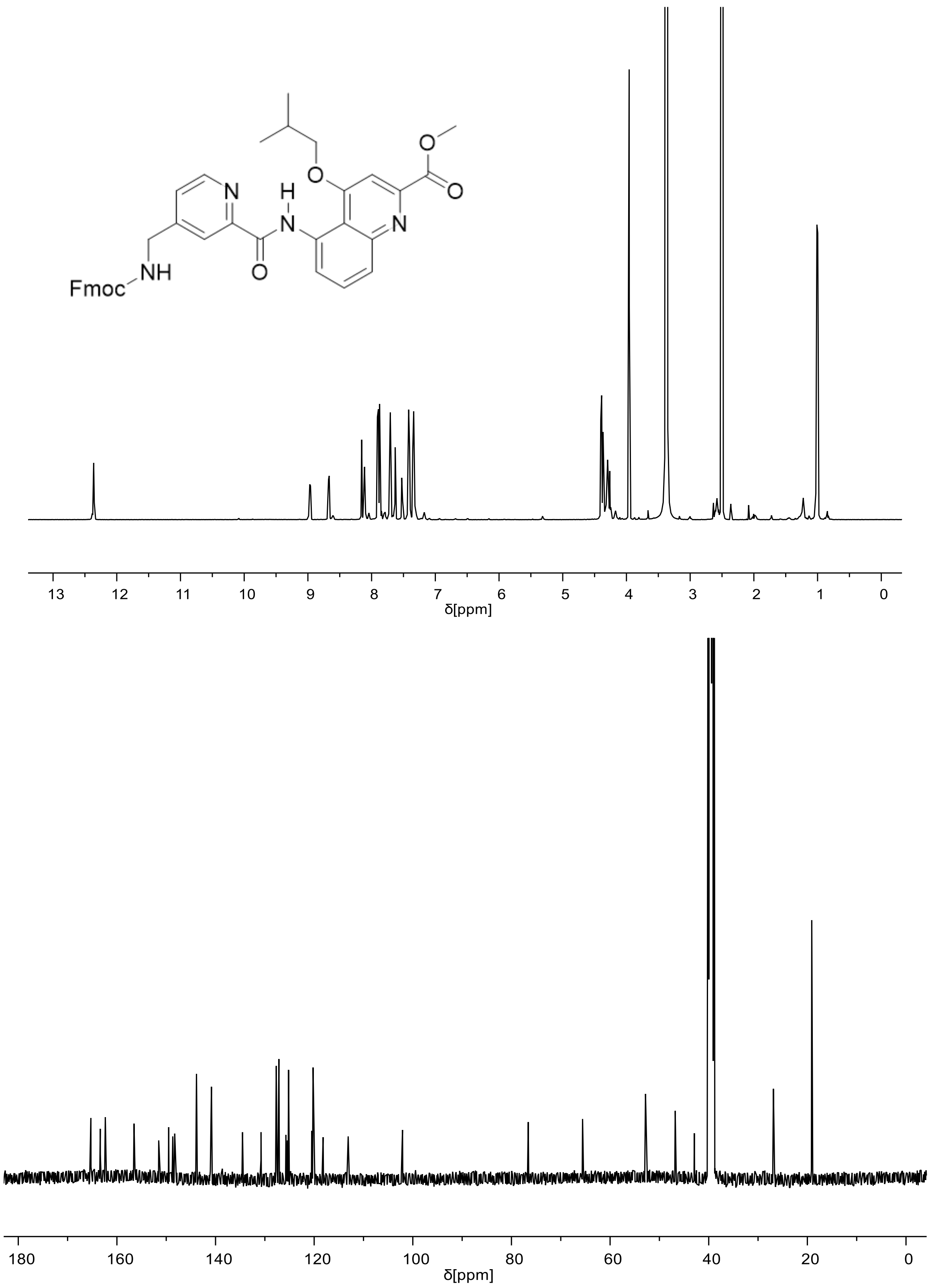

^{1}H NMR spectrum (500 MHz, DMSO-d_6) and ^{13}C NMR (126 MHz, DMSO-d_6) of compound **21**.

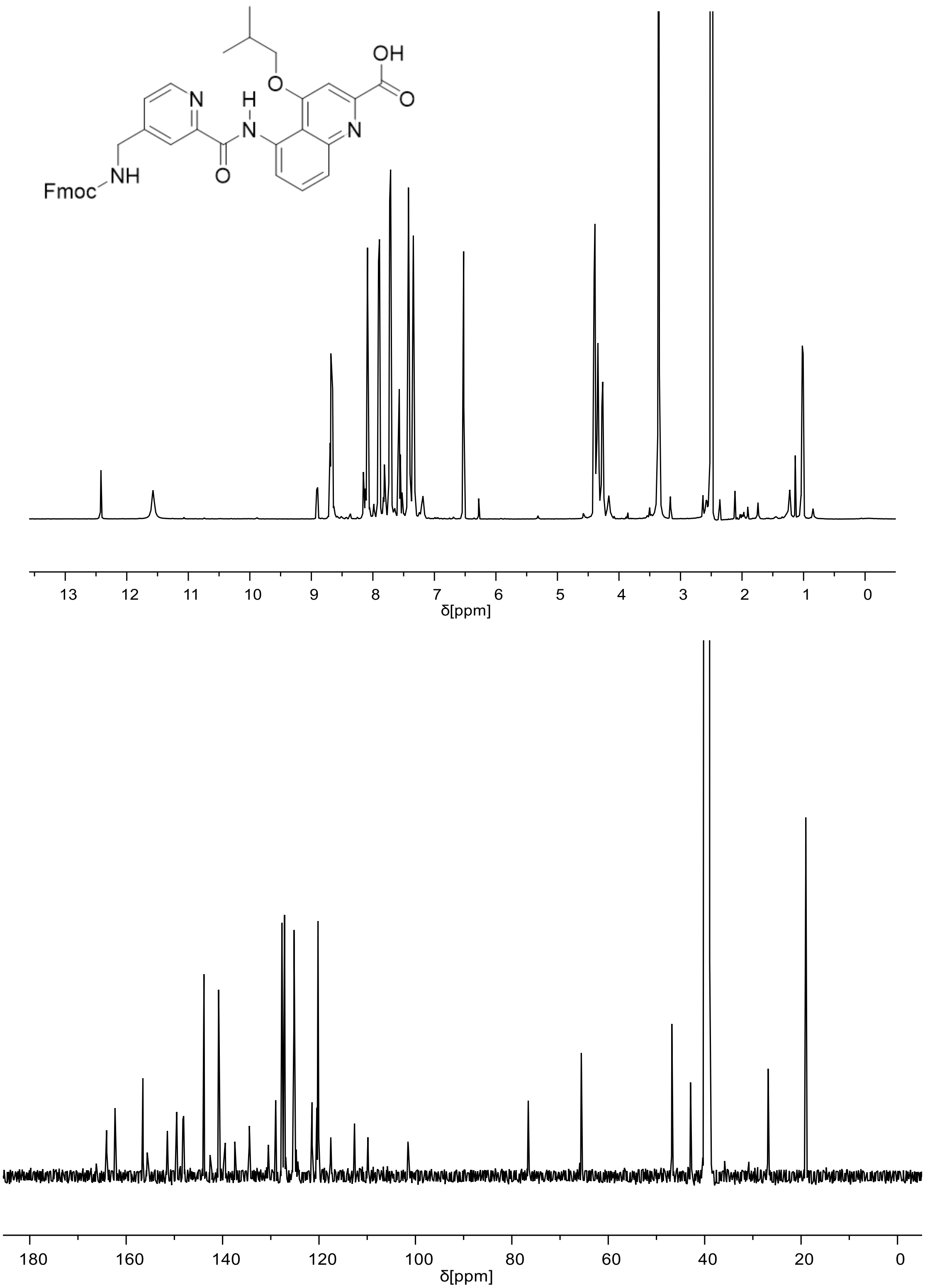

^{1}H NMR spectrum (500 MHz, DMSO-d_6) and ^{13}C NMR (126 MHz, DMSO-d_6) of compound **22**.

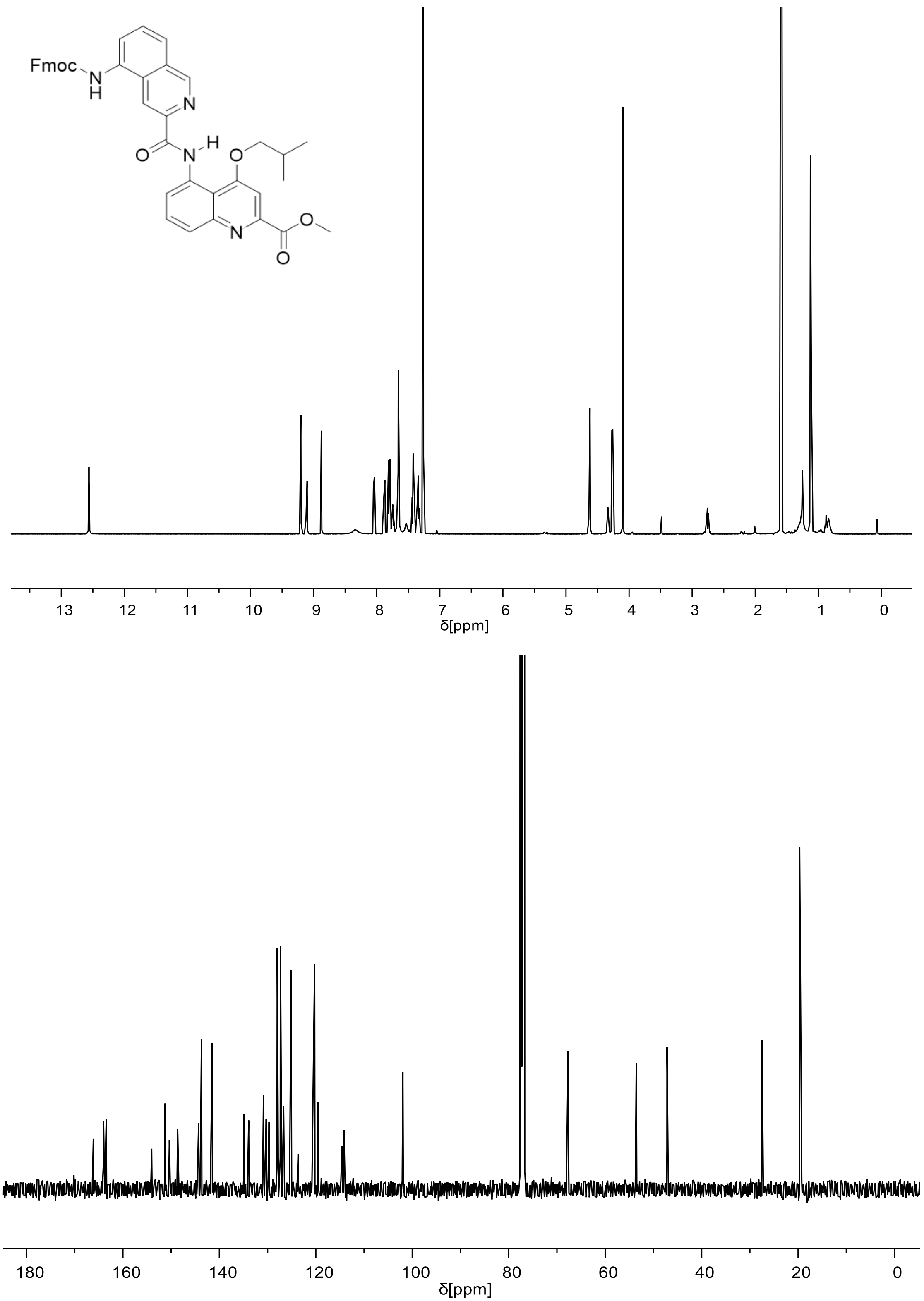

^{1}H NMR spectrum (500 MHz, $CDCl_3$) and ^{13}C NMR (126 MHz, $CDCl_3$) of compound **23**.

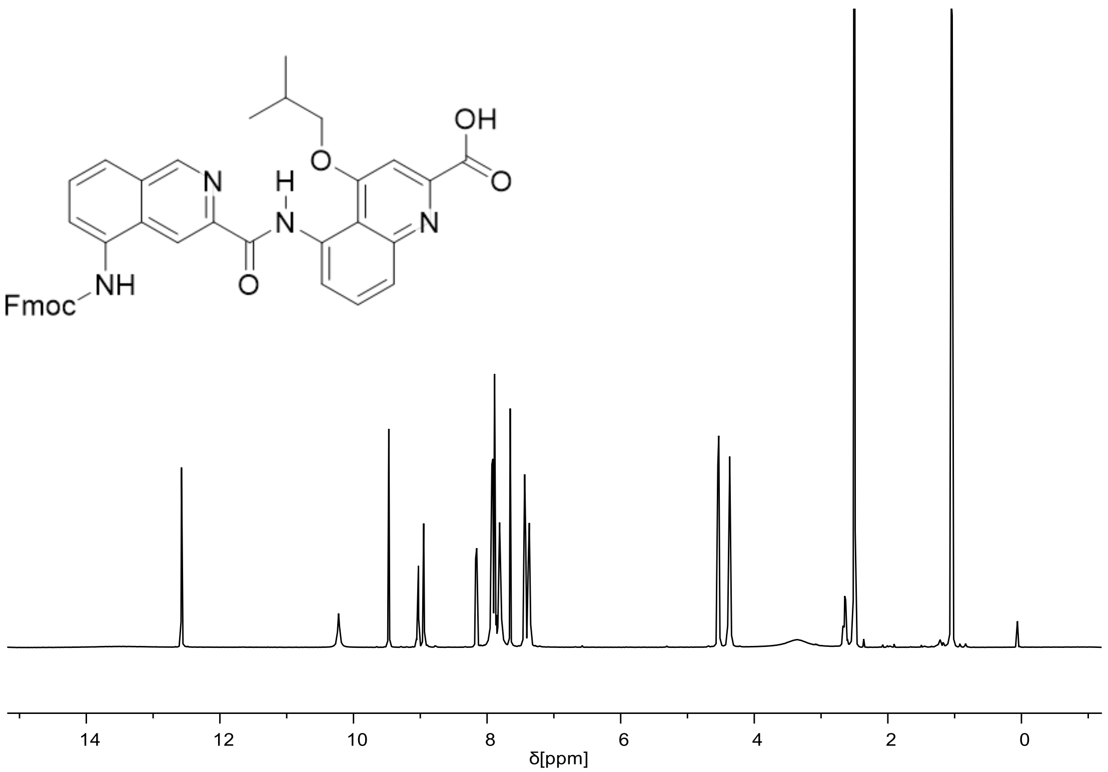

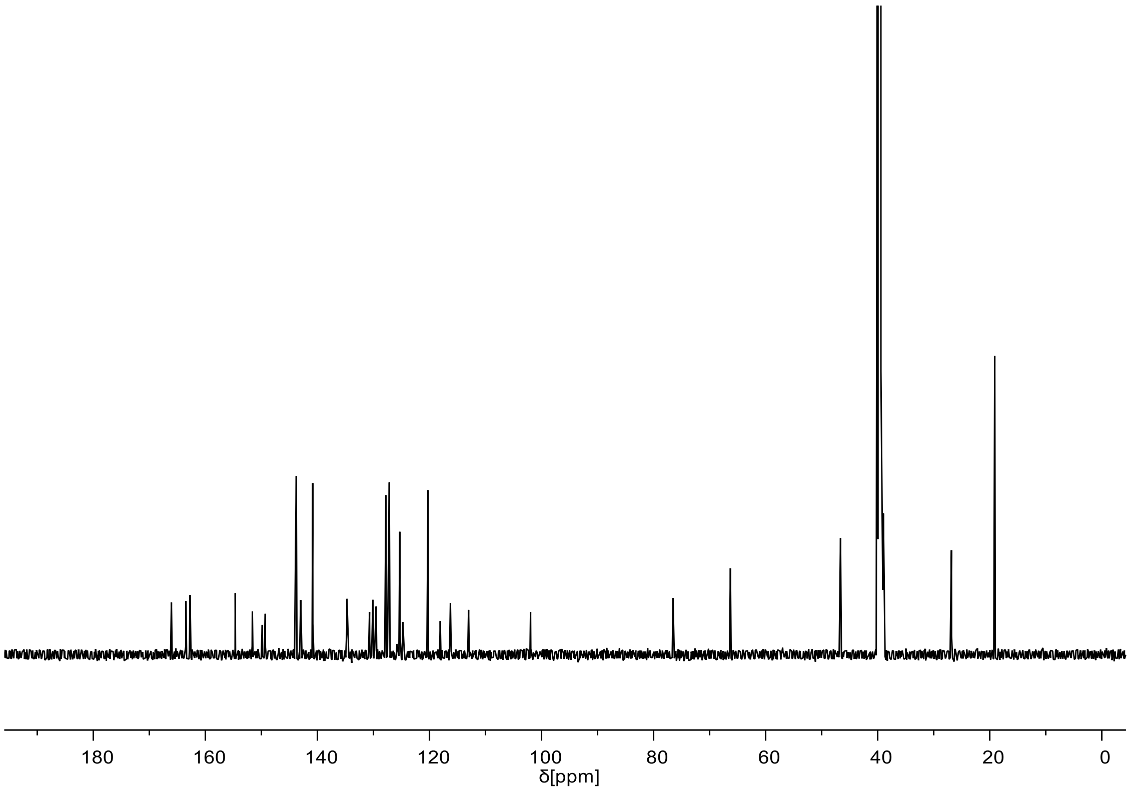

^{1}H NMR spectrum (500 MHz, DMSO-d_6) and ^{13}C NMR (126 MHz, DMSO-d_6) of compound **24**.

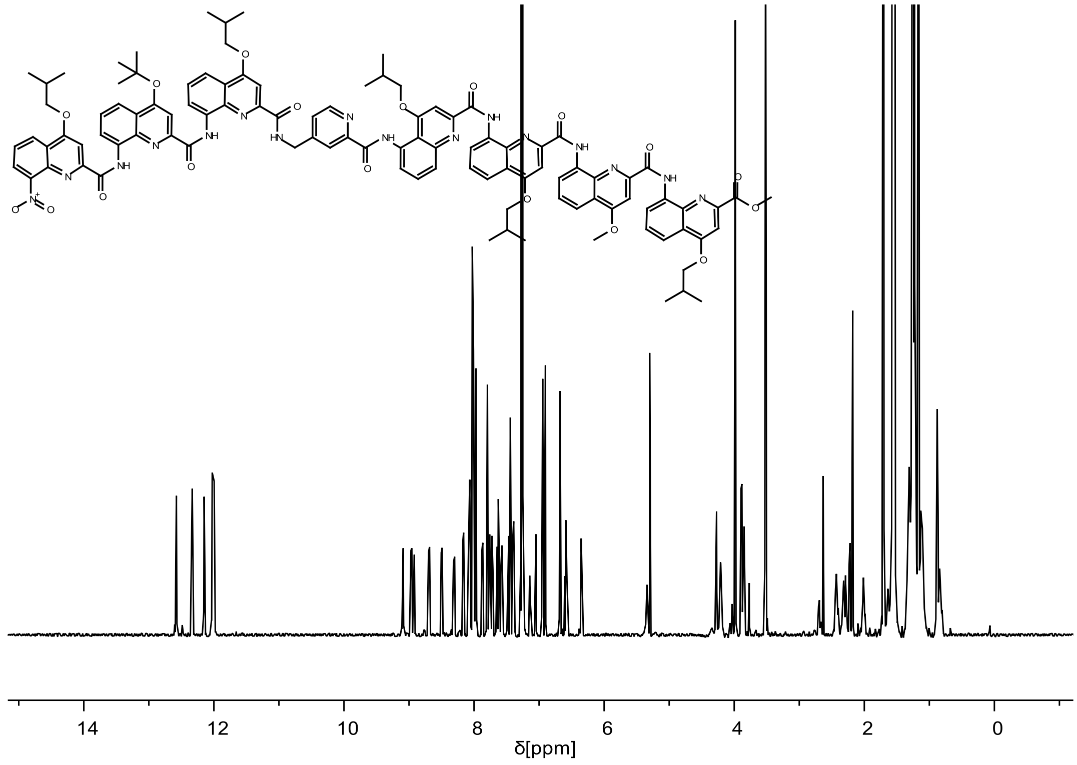

^1H NMR spectrum (500 MHz, $CDCl_3$) of compound **2a**.

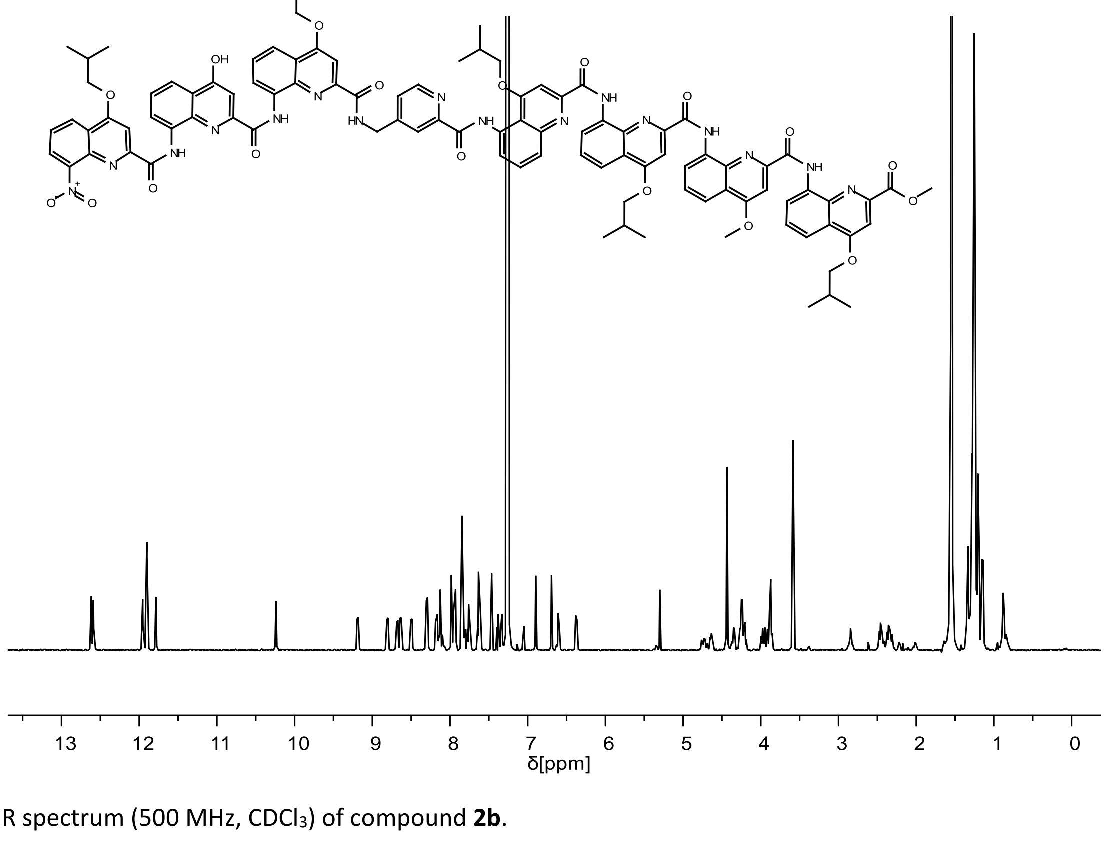

^1H NMR spectrum (500 MHz, $CDCl_3$) of compound **2b**.

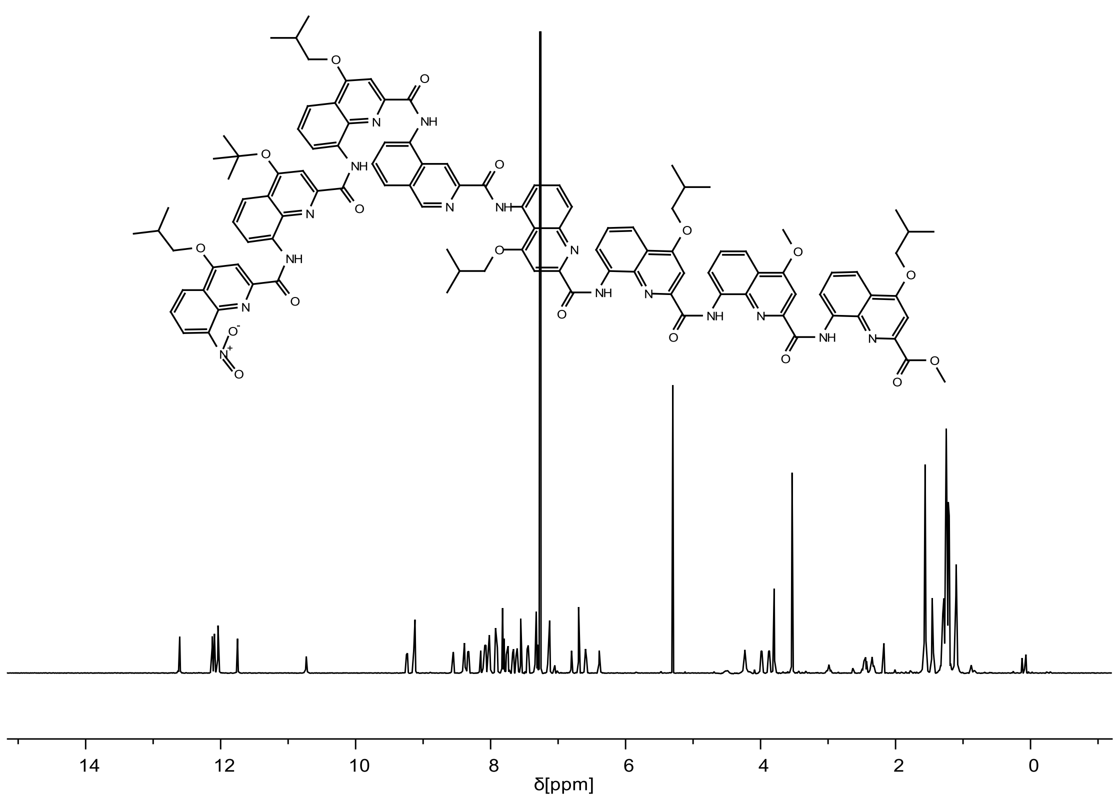

^{1}H NMR spectrum (500 MHz, $CDCl_3$) of compound **3a**.

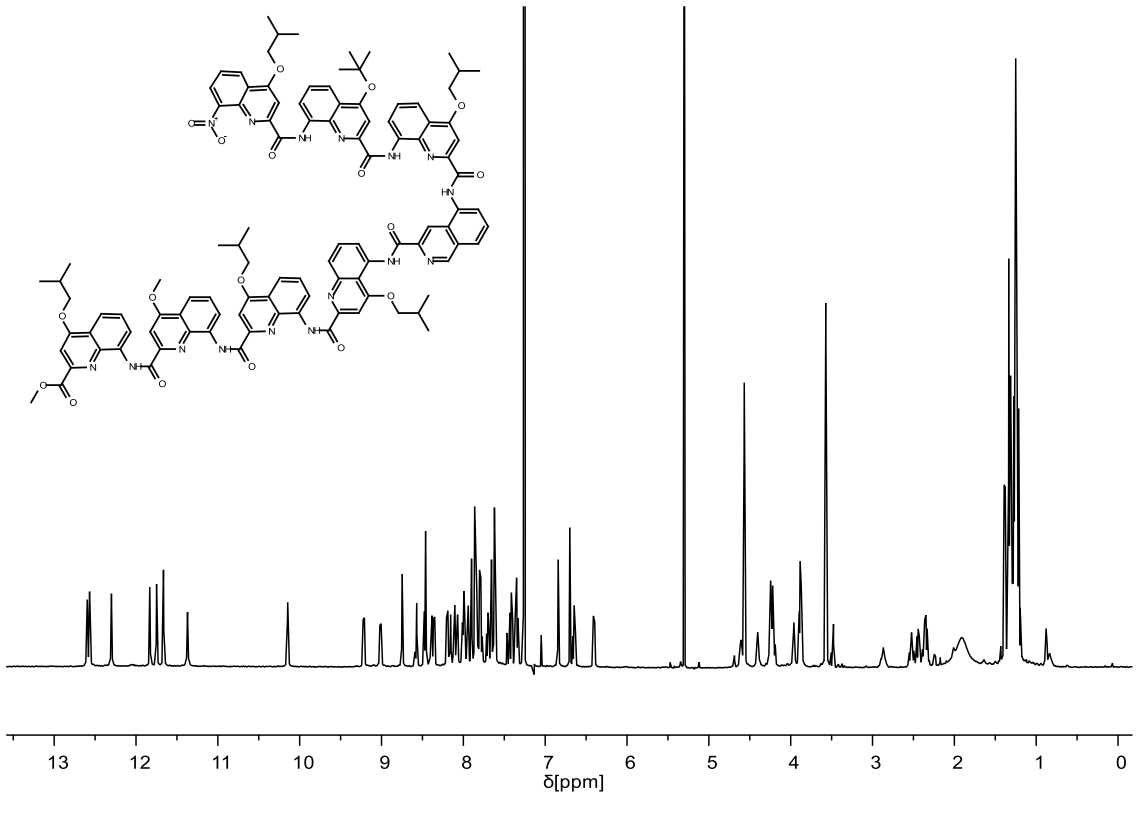

^{1}H NMR spectrum (500 MHz, $CDCl_3$) of compound **3b**.

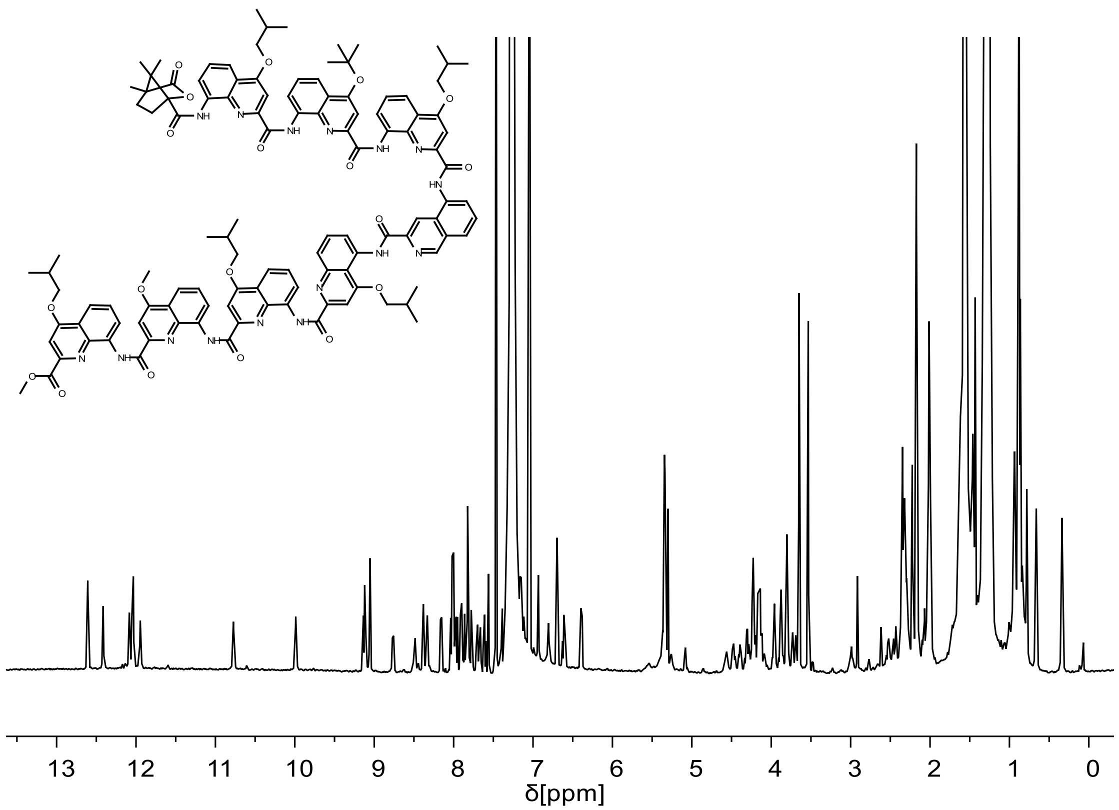

^{1}H NMR spectrum (500 MHz, $CDCl_3$) of compound **4a**.

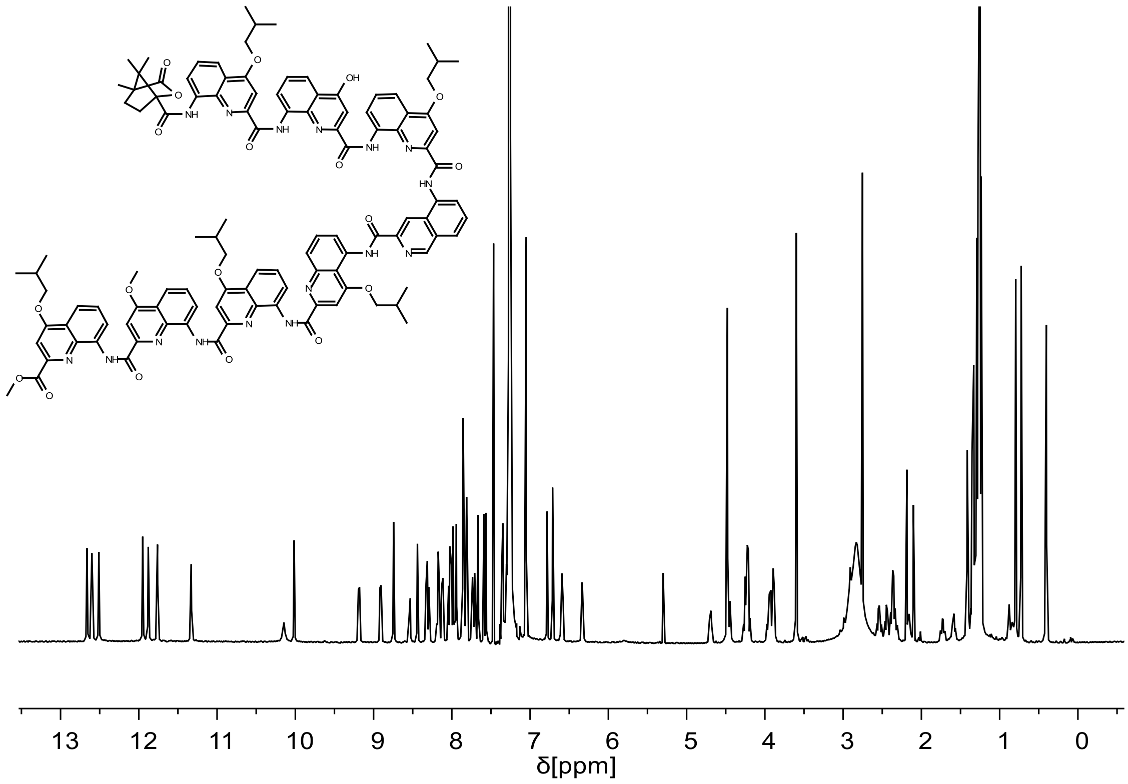

^{1}H NMR spectrum (500 MHz, $CDCl_3$) of compound **4b**.

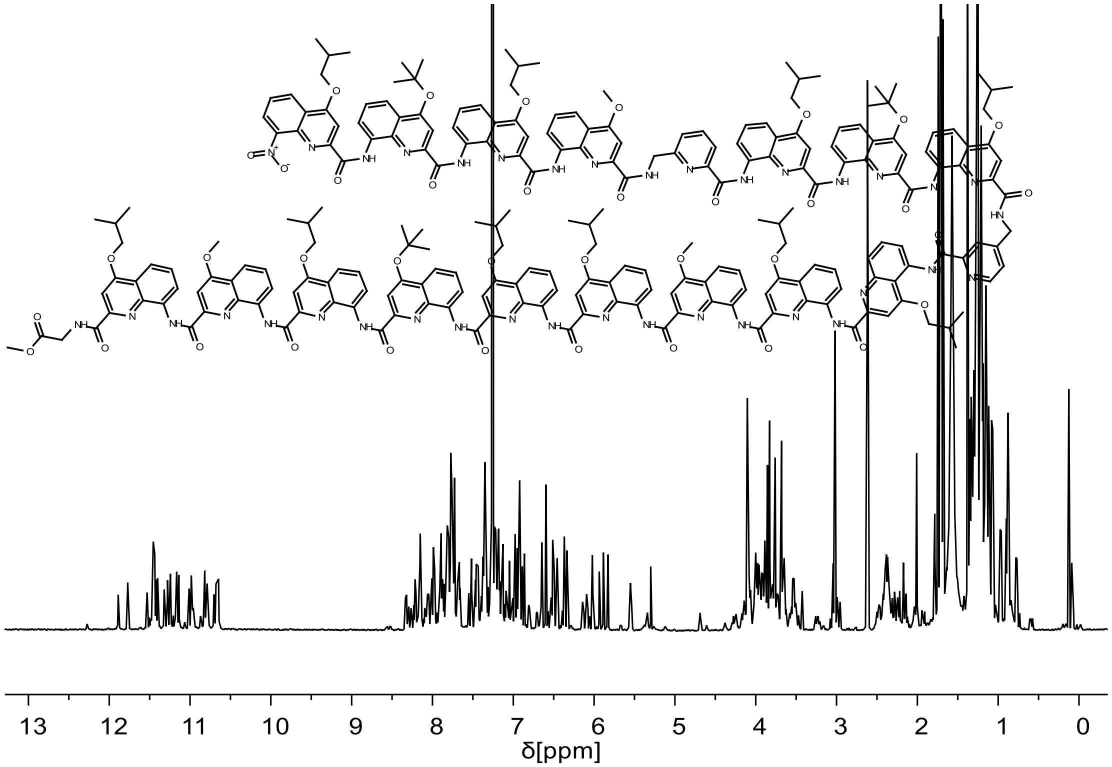

^{1}H NMR spectrum (500 MHz, $CDCl_3$) of compound **5a**.

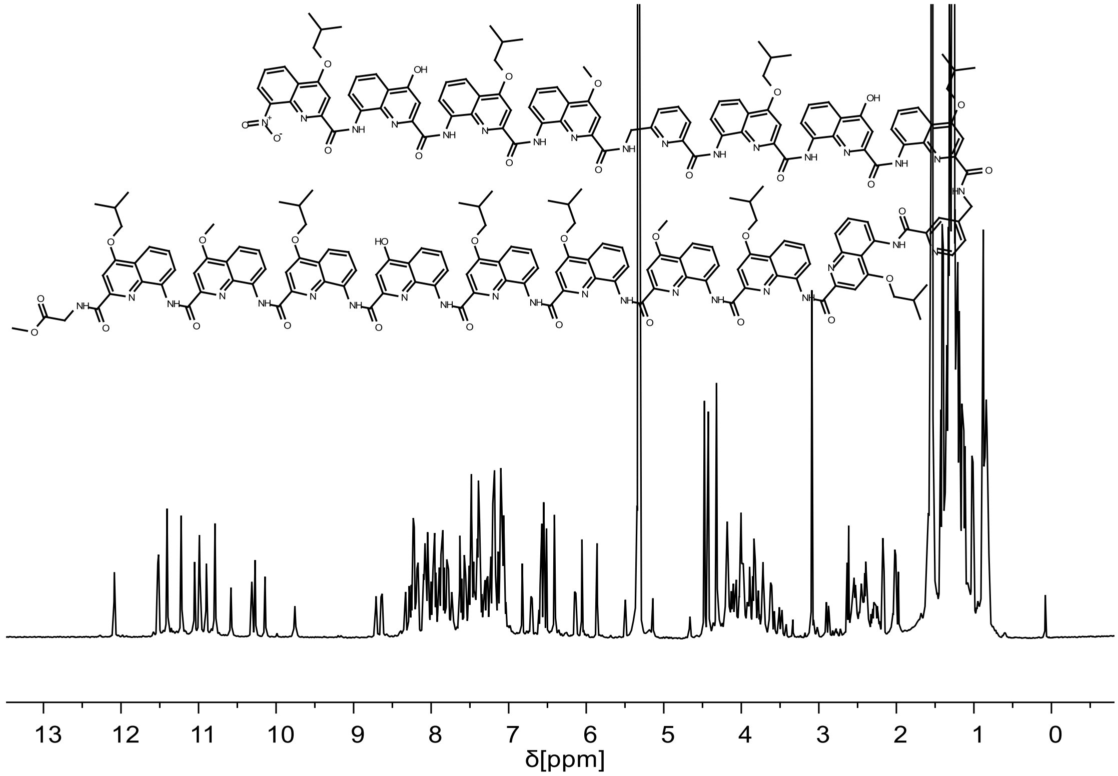

^{1}H NMR spectrum (500 MHz, CD_2Cl_2) of compound **5b**.

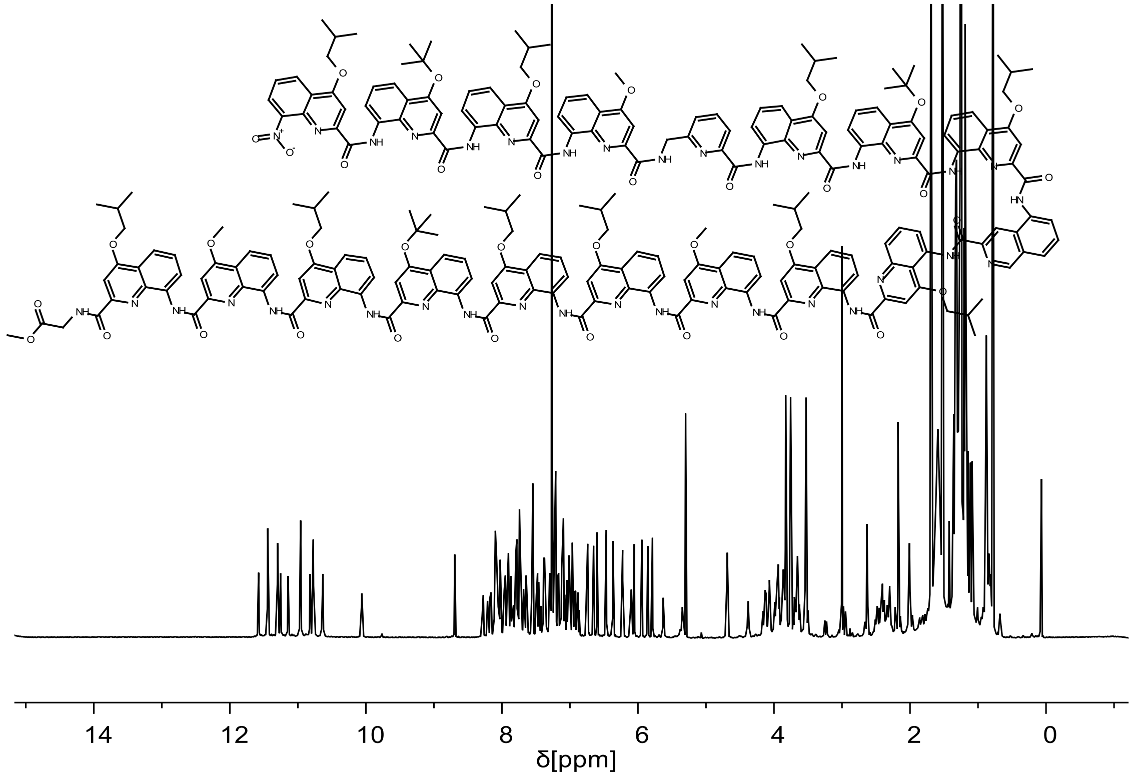

^{1}H NMR spectrum (500 MHz, $CDCl_3$) of compound **6a**.

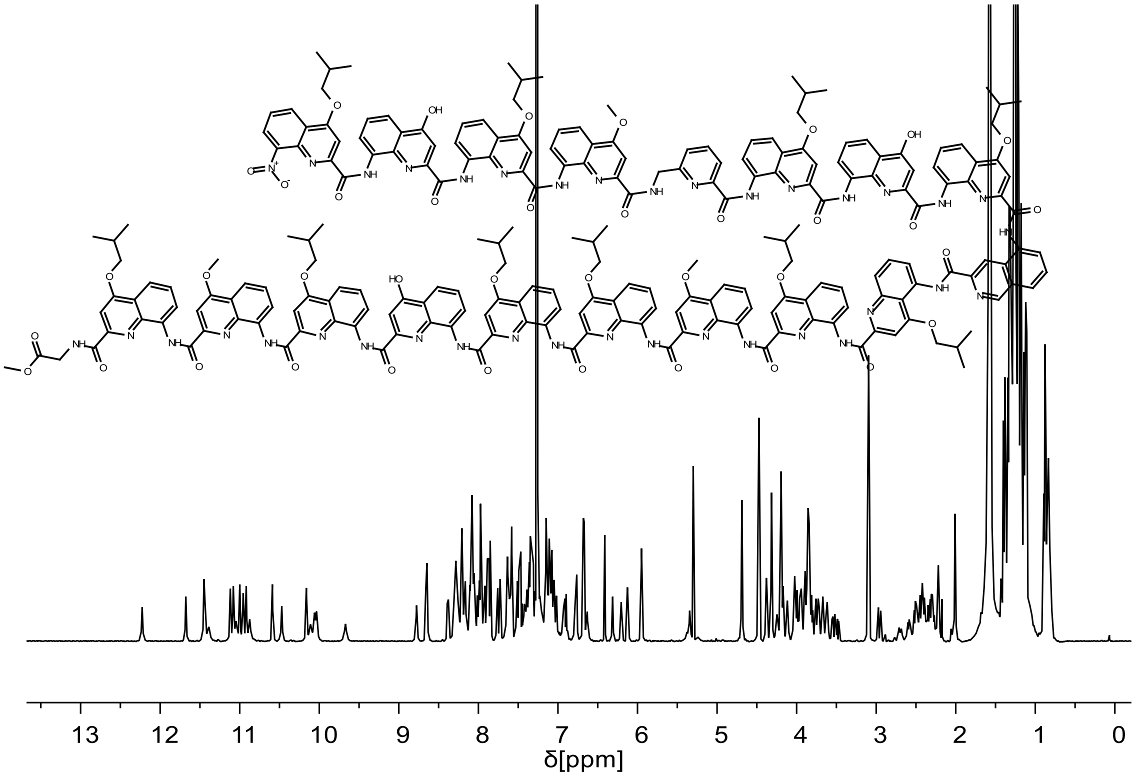

^{1}H NMR spectrum (500 MHz, $CDCl_3$) of compound **6b**.

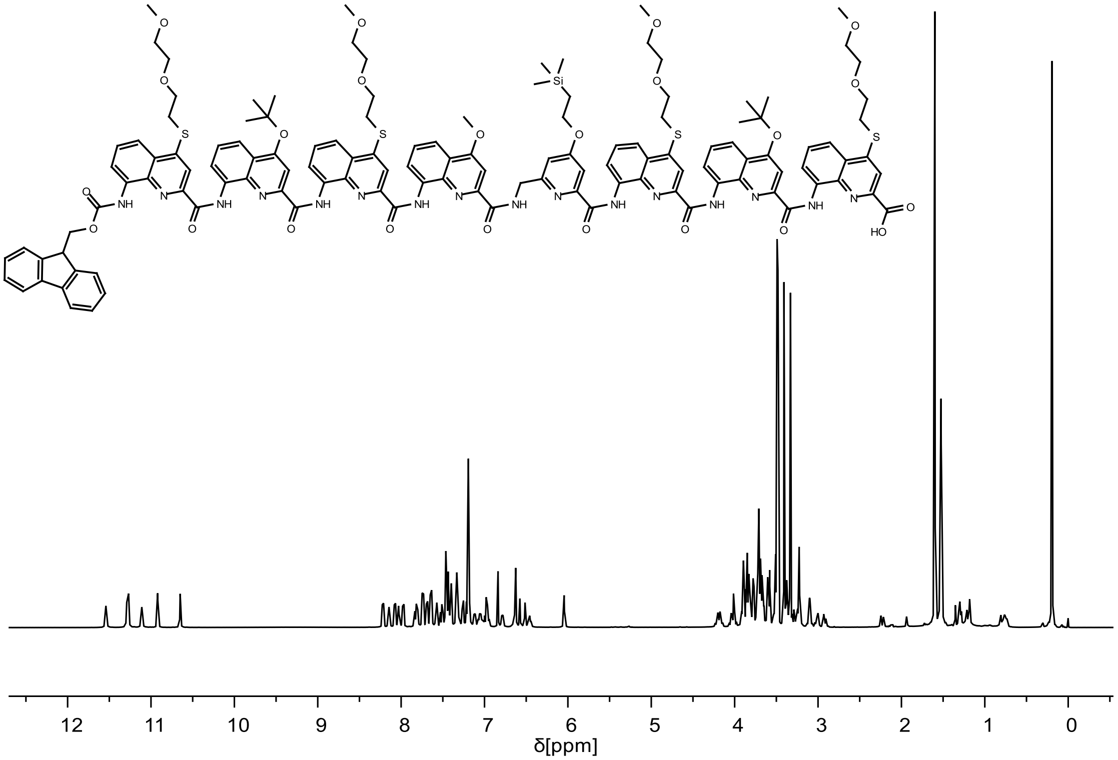

^{1}H NMR spectrum (500 MHz, $CDCl_3$) of compound **25**.

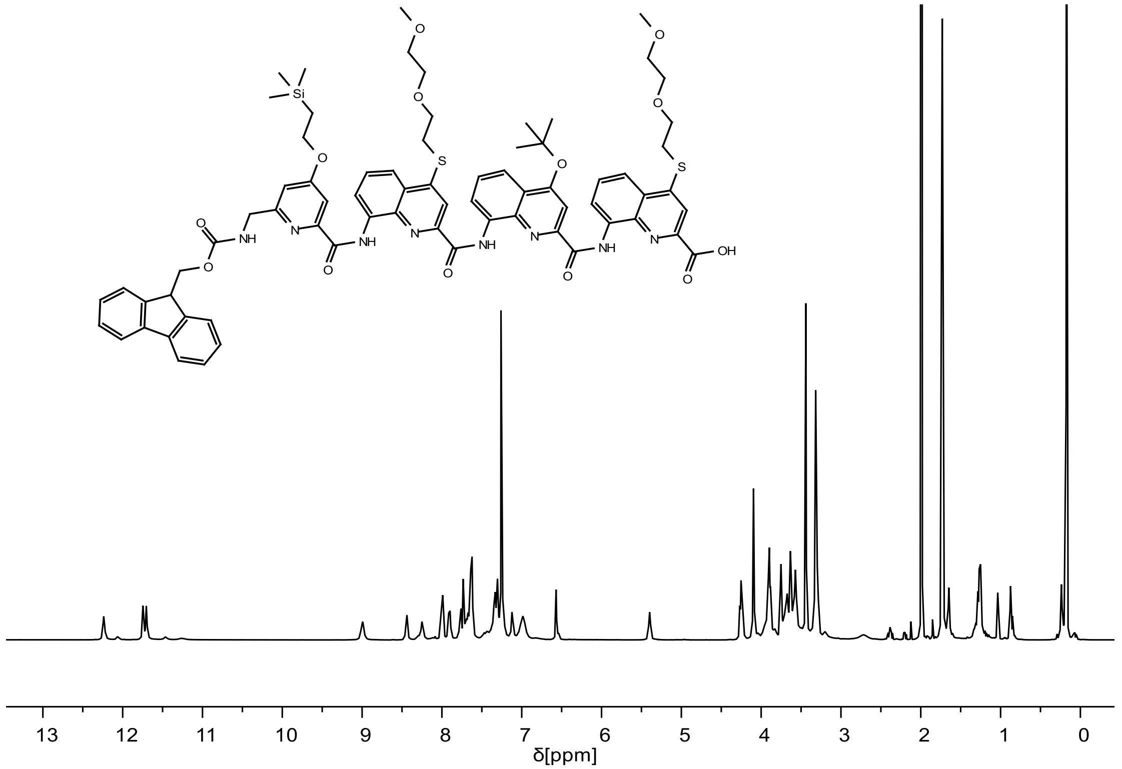

^{1}H NMR spectrum (500 MHz, $CDCl_3$) of compound **26**.

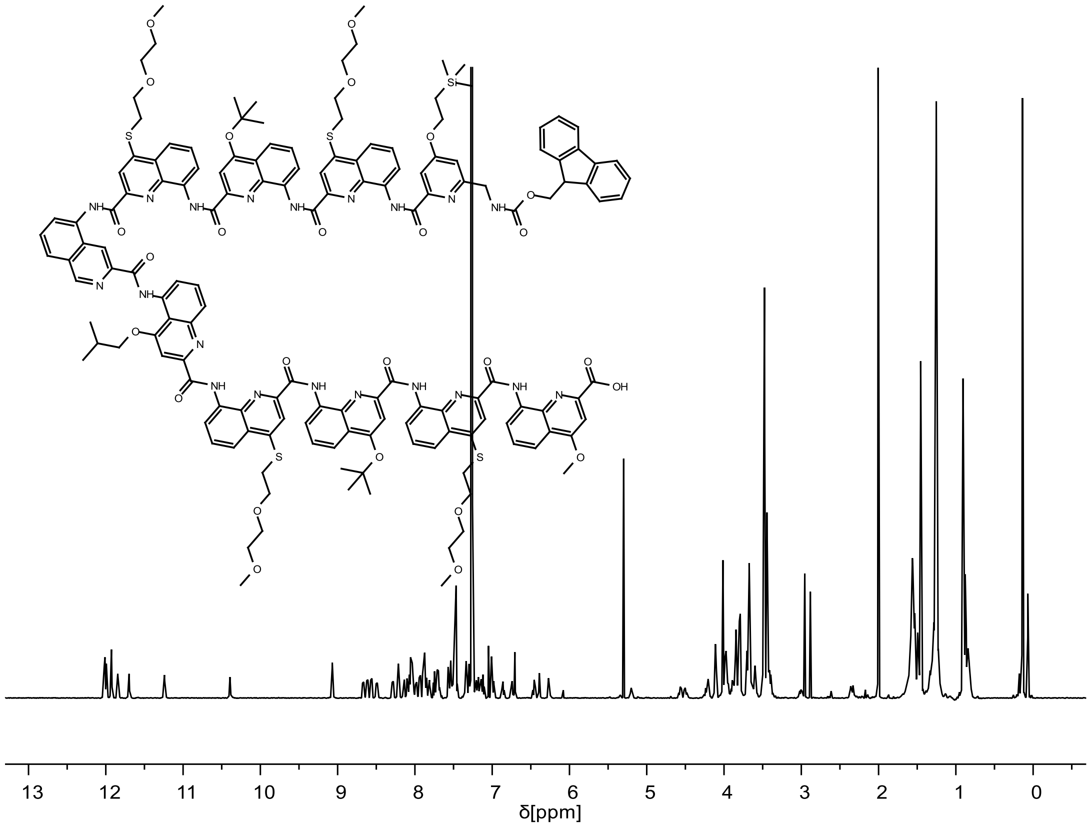

^{1}H NMR spectrum (500 MHz, $CDCl_3$) of compound **27**.

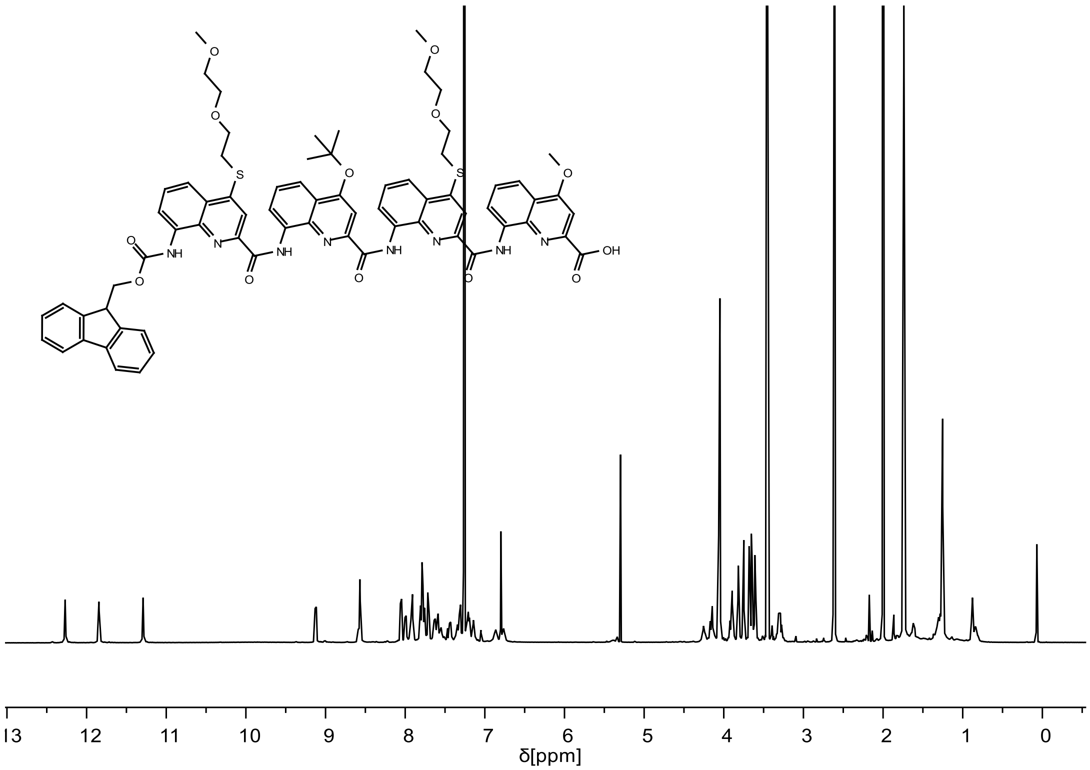

^{1}H NMR spectrum (500 MHz, $CDCl_3$) of compound **28**.

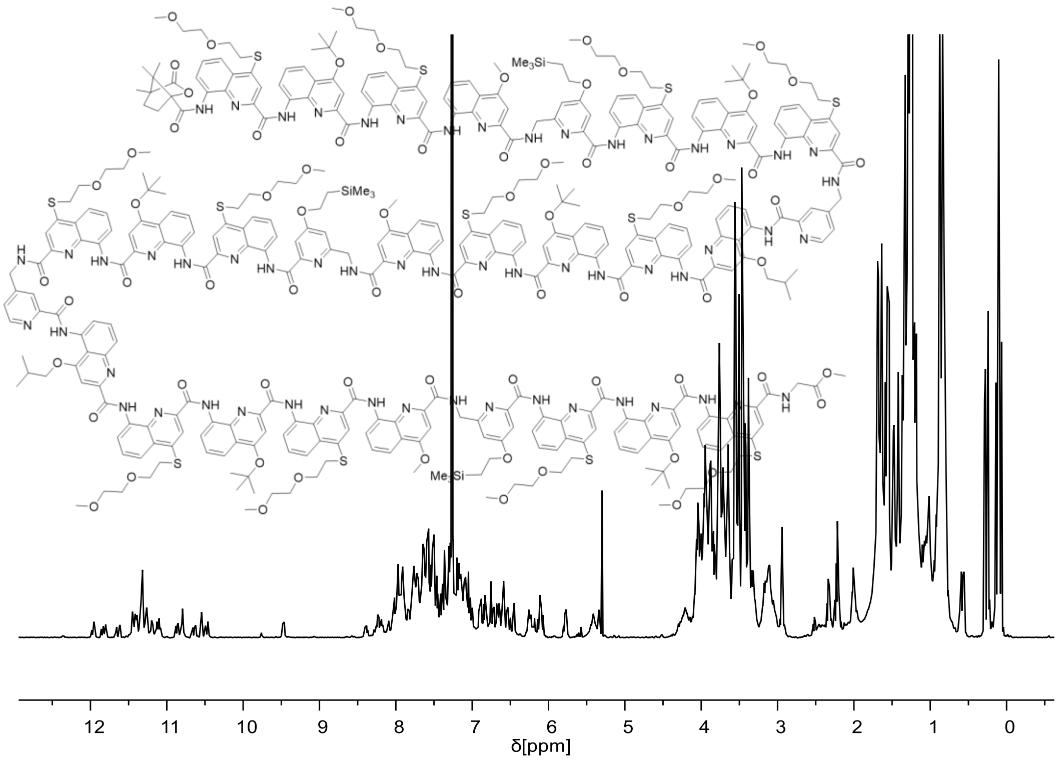

^{1}H NMR spectrum (500 MHz, $CDCl_3$) of compound **7a**.

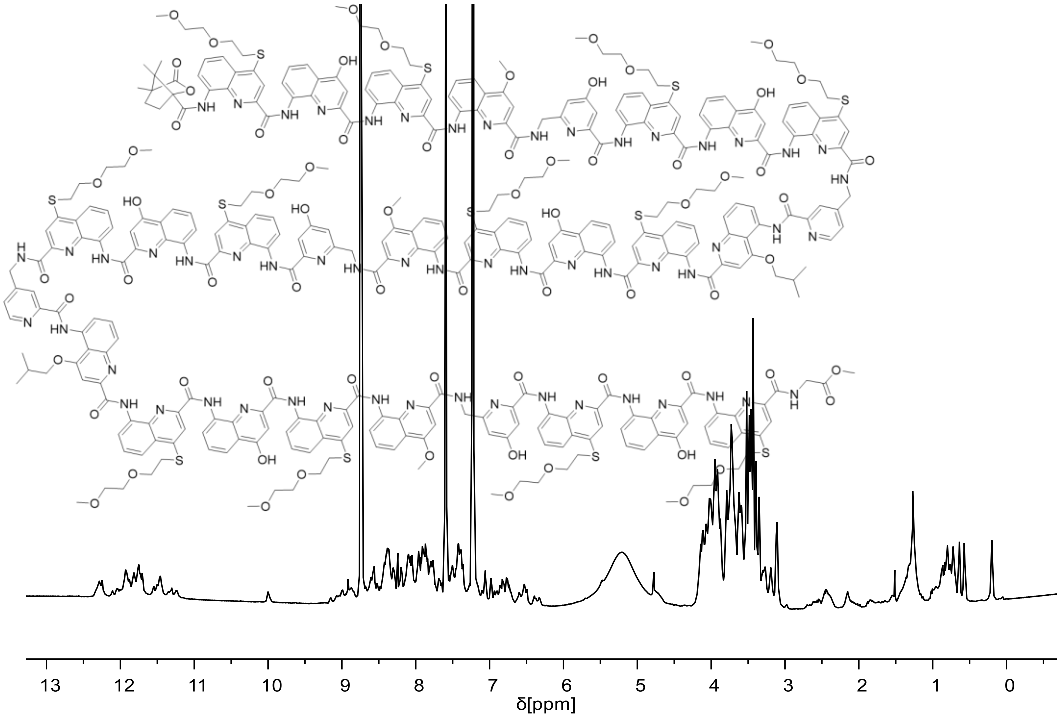

^{1}H NMR spectrum (500 MHz, pyridine-d_5) of compound **7b**.

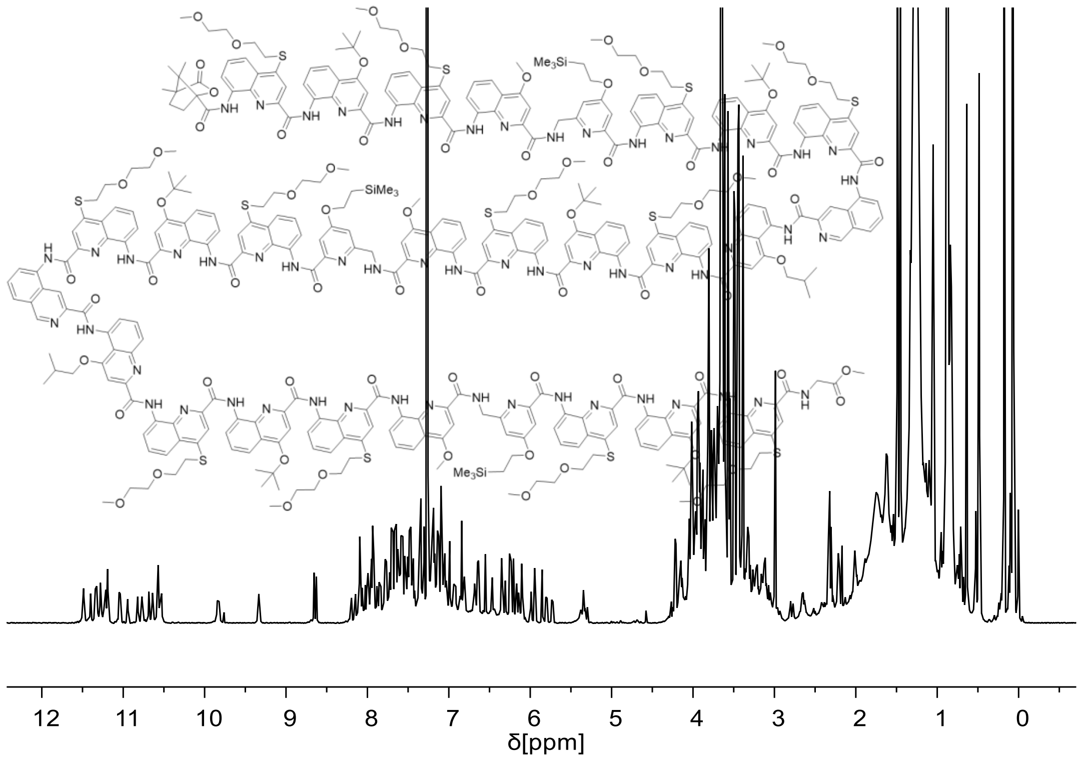

^{1}H NMR spectrum (500 MHz, $CDCl_3$) of compound **8a**.

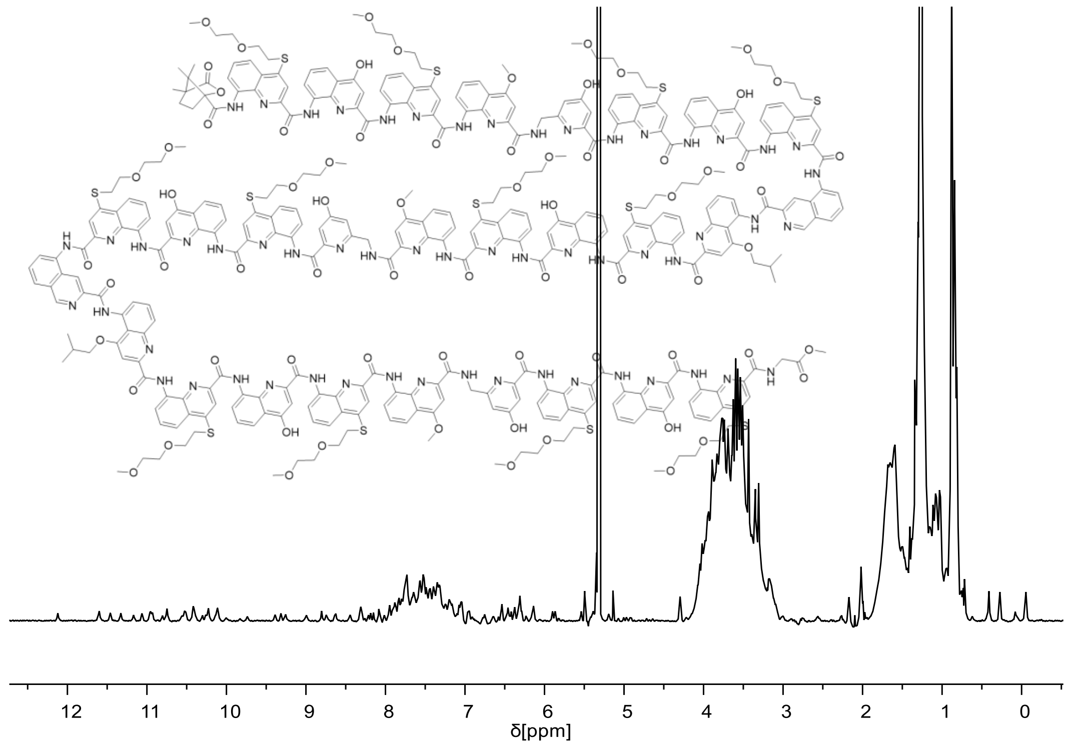

^{1}H NMR spectrum (500 MHz, CD_2Cl_2) of compound **8b**.

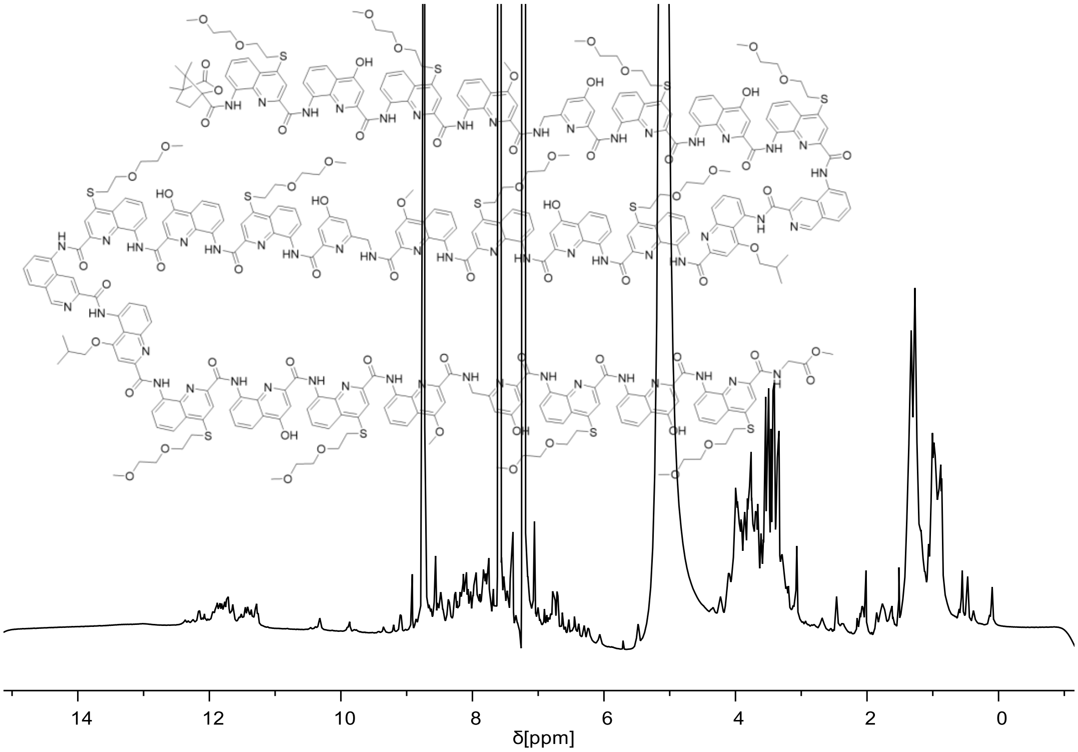

^{1}H NMR spectrum (500 MHz, pyridine-d_5) of compound **8b**.

8 MS spectra of 7a, 7b, 8a and 8b

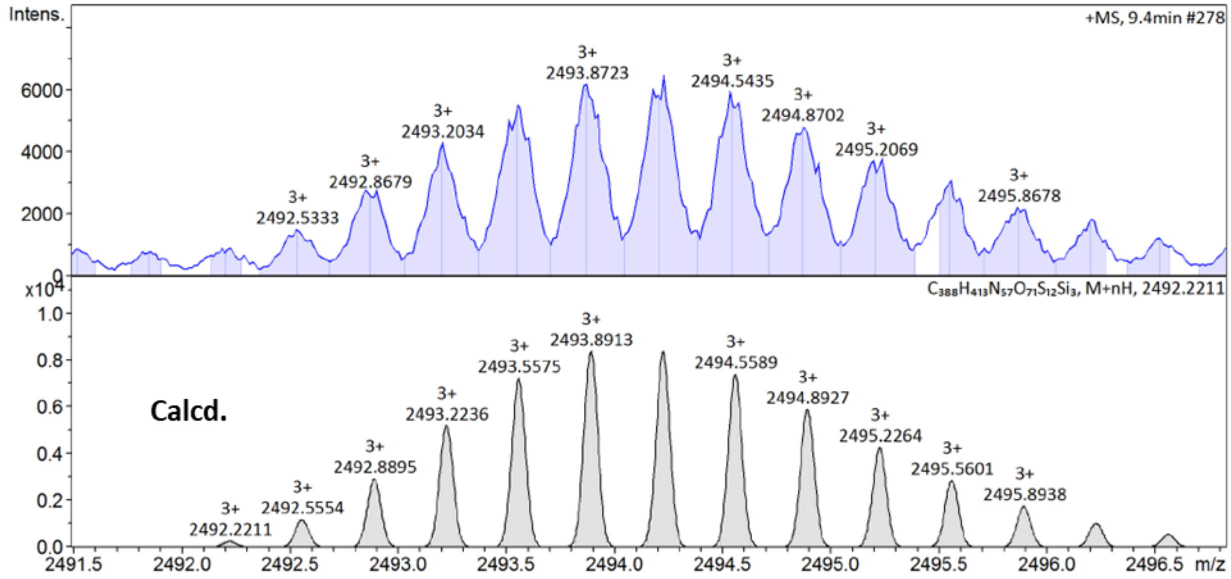

HRMS (ESI+) of compound **7a**.

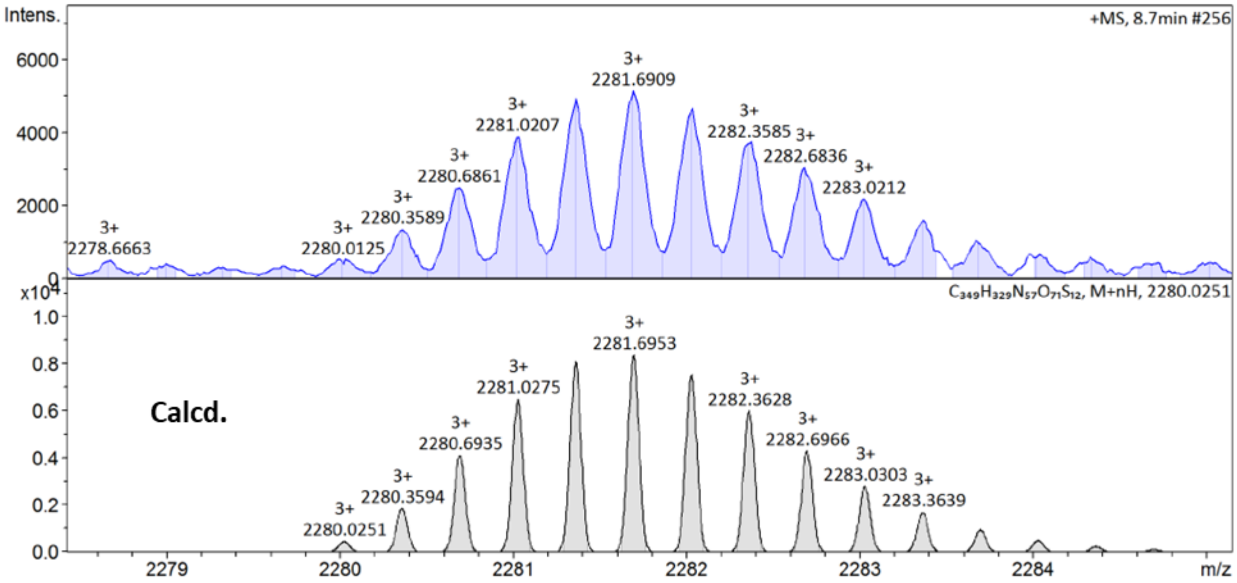

HRMS (ESI+) of compound **7b**.

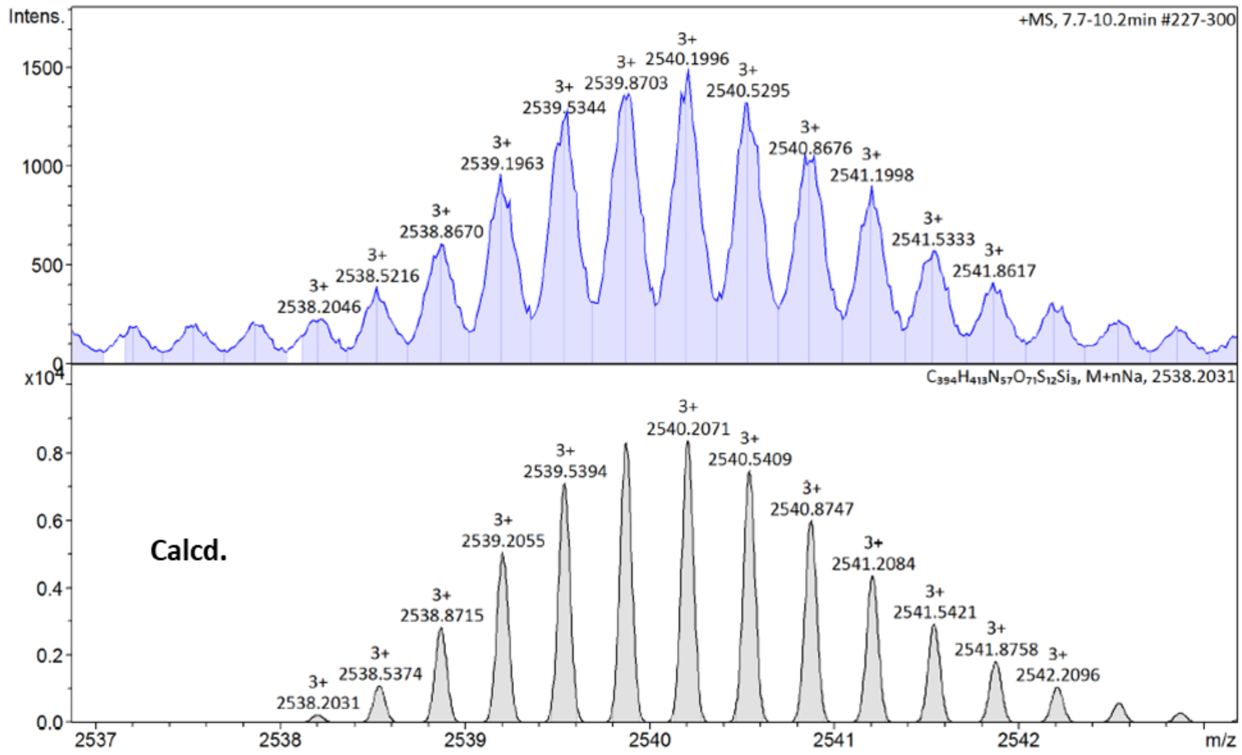

HRMS (ESI+) of compound **8a**.

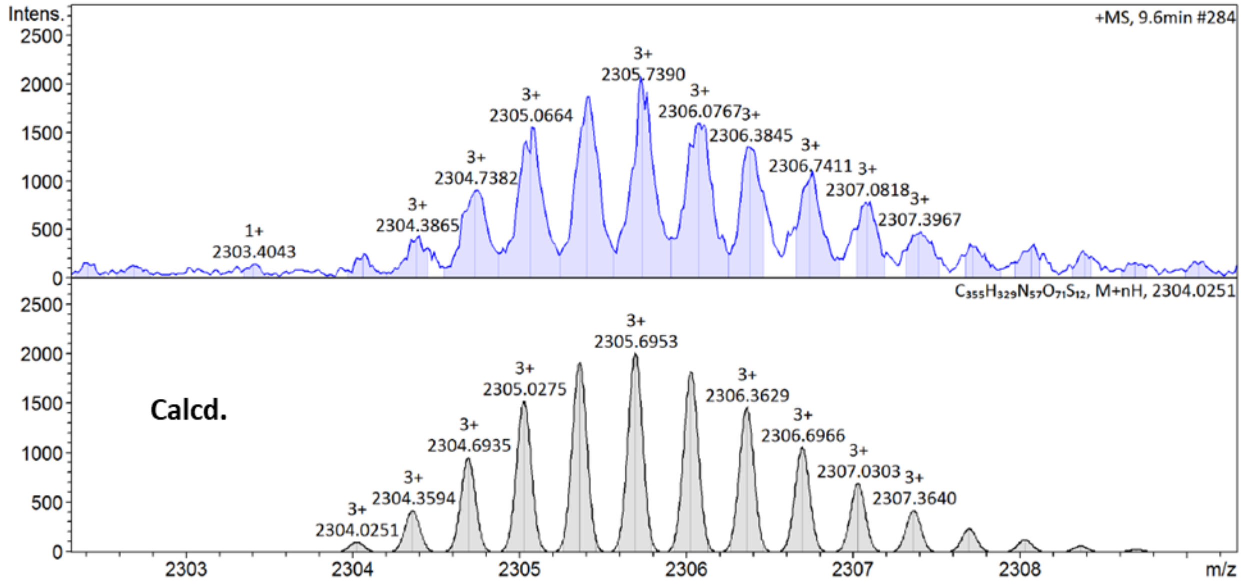

HRMS (ESI+) of compound **8b**.
